# A genome-wide cross-phenotype meta-analysis of the association of blood pressure with migraine

**DOI:** 10.1038/s41467-020-17002-0

**Published:** 2020-07-06

**Authors:** Yanjun Guo, Pamela M. Rist, Iyas Daghlas, Franco Giulianini, The International Headache Genetics Consortium, Padhraig Gormley, Verneri Anttila, Bendik S. Winsvold, Priit Palta, Tonu Esko, Tune H. Pers, Kai-How Farh, Ester Cuenca-Leon, Mikko Muona, Nicholas A. Furlotte, Tobias Kurth, Andres Ingason, George McMahon, Lannie Ligthart, Gisela M. Terwindt, Mikko Kallela, Tobias M. Freilinger, Caroline Ran, Scott G. Gordon, Anine H. Stam, Stacy Steinberg, Guntram Borck, Markku Koiranen, Lydia Quaye, Hieab H. H. Adams, Terho Lehtimäki, Antti-Pekka Sarin, Juho Wedenoja, David A. Hinds, Julie E. Buring, Markus Schürks, Paul M. Ridker, Maria Gudlaug Hrafnsdottir, Hreinn Stefansson, Susan M. Ring, Jouke-Jan Hottenga, Brenda W. J. H. Penninx, Markus Färkkilä, Ville Artto, Mari Kaunisto, Salli Vepsäläinen, Rainer Malik, Andrew C. Heath, Pamela A. F. Madden, Nicholas G. Martin, Grant W. Montgomery, Mitja Kurki, Mart Kals, Reedik Mägi, Kalle Pärn, Eija Hämäläinen, Hailiang Huang, Andrea E. Byrnes, Lude Franke, Jie Huang, Evie Stergiakouli, Phil H. Lee, Cynthia Sandor, Caleb Webber, Zameel Cader, Bertram Muller-Myhsok, Stefan Schreiber, Thomas Meitinger, Johan G. Eriksson, Veikko Salomaa, Kauko Heikkilä, Elizabeth Loehrer, Andre G. Uitterlinden, Albert Hofman, Cornelia M. van Duijn, Lynn Cherkas, Linda M. Pedersen, Audun Stubhaug, Christopher S. Nielsen, Minna Männikkö, Evelin Mihailov, Lili Milani, Hartmut Göbel, Ann-Louise Esserlind, Anne Francke Christensen, Thomas Folkmann Hansen, Thomas Werge, Jaakko Kaprio, Arpo J. Aromaa, Olli Raitakari, M. Arfan Ikram, Tim Spector, Marjo-Riitta Järvelin, Andres Metspalu, Christian Kubisch, David P. Strachan, Michel D. Ferrari, Andrea C. Belin, Martin Dichgans, Maija Wessman, Arn M. J. M. van den Maagdenberg, John-Anker Zwart, Dorret I. Boomsma, George Davey Smith, Kari Stefansson, Nicholas Eriksson, Mark J. Daly, Benjamin M. Neale, Jes Olesen, Daniel I. Chasman, Dale R. Nyholt, Aarno Palotie, The 23andMe Research Team, Michelle Agee, Adam Auton, Robert K. Bell, Katarzyna Bryc, Sarah L. Elson, Pierre Fontanillas, Nicholas A. Furlotte, David A. Hinds, Karen E. Huber, Aaron Kleinman, Nadia K. Litterman, Jennifer C. McCreight, Matthew H. McIntyre, Joanna L. Mountain, Elizabeth S. Noblin, Carrie A. M. Northover, Steven J. Pitts, J. Fah Sathirapongsasuti, Olga V. Sazonova, Janie F. Shelton, Suyash Shringarpure, Chao Tian, Joyce Y. Tung, Vladimir Vacic, Tobias Kurth, Daniel I. Chasman

**Affiliations:** 1 Division of Preventive Medicine, Brigham and Women’s Hospital, Boston, MA 02215 USA; 2 Harvard Medical School, Boston, MA 02115 USA; 3 Department of Epidemiology, Harvard T.H. Chan School of Public Health, Boston, MA 02215 USA; 4 Institute of Public Health, Charité – Universitätsmedizin Berlin, Charitéplatz 1, 10117 Berlin, Germany; 5 Psychiatric and Neurodevelopmental Genetics Unit, Massachusetts General Hospital and Harvard Medical School, Boston, MA USA; 6 Medical and Population Genetics Program, Broad Institute of MIT and Harvard, Cambridge, MA USA; 7 Stanley Center for Psychiatric Research, Broad Institute of MIT and Harvard, Cambridge, MA USA; 8 Wellcome Trust Sanger Institute, Wellcome Trust Genome Campus, Hinxton, UK; 9 Analytic and Translational Genetics Unit, Massachusetts General Hospital and Harvard Medical School, Boston, MA USA; 10 FORMI, Oslo University Hospital, P.O. 4956 Nydalen, 0424 Oslo, Norway; 11 Department of Neurology, Oslo University Hospital, P.O. 4956 Nydalen, 0424 Oslo, Norway; 12 Institute of Clinical Medicine, University of Oslo, P.O. 1171 Blindern, 0318 Oslo, Norway; 13 Institute for Molecular Medicine Finland (FIMM), University of Helsinki, Helsinki, Finland; 14 Estonian Genome Center, University of Tartu, Tartu, Estonia; 15 Division of Endocrinology, Boston Children’s Hospital, Boston, MA USA; 16 Department of Epidemiology Research, Statens Serum Institut, Copenhagen, Denmark; 17 Novo Nordisk Foundation Center for Basic Metabolic Research, University of Copenhagen, Copenhagen, Denmark; 18 Illumina, Illumina Way, San Diego, CA 5200 USA; 19 Vall d’Hebron Research Institute, Pediatric Neurology, Barcelona, Spain; 20 Folkhälsan Institute of Genetics, FI-00290 Helsinki, Finland; 21 Neuroscience Center, University of Helsinki, FI-00014 Helsinki, Finland; 22 Research Programs Unit, Molecular Neurology, University of Helsinki, FI-00014 Helsinki, Finland; 23 23andMe, Inc., 223 N Mathilda Ave, Sunnyvale, CA 94086 USA; 24 Inserm Research Center for Epidemiology and Biostatistics (U897), University of Bordeaux, 33076 Bordeaux, France; 25 Division of Preventive Medicine, Brigham and Women’s Hospital, Boston, MA 02215 USA; 26 deCODE Genetics, 101 Reykjavik, Iceland; 27 Medical Research Council (MRC) Integrative Epidemiology Unit, University of Bristol, Bristol, UK; 28 Department of Biological Psychology, VU University Amsterdam, 1081 BT Amsterdam, The Netherlands; 29 Department of Neurology, Leiden University Medical Centre, PO Box 9600, 2300 RC Leiden, The Netherlands; 30 Department of Neurology, Helsinki University Central Hospital, Haartmaninkatu 4, 00290 Helsinki, Finland; 31 Institute for Stroke and Dementia Research, Klinikum der Universtität München, Ludwig-Maximilians-Universität München, Feodor-Lynen-Str. 17, 81377 Munich, Germany; 32 Department of Neurology and Epileptology, Hertie Institute for Clincal Brain Research, University of Tuebingen, Tuebingen, Germany; 33 Department of Neuroscience, Karolinska Institutet, 171 77 Stockholm, Sweden; 34 Department of Genetics and Computational Biology, QIMR Berghofer Medical Research Institute, 300 Herston Road, Brisbane, QLD 4006 Australia; 35 Institute of Human Genetics, Ulm University, 89081 Ulm, Germany; 36 Center for Life Course Epidemiology and Systems Medicine, University of Oulu, Box 5000, FI-90014 Oulu, Finland; 37 Department of Twin Research and Genetic Epidemiology, King’s College London, London, UK; 38 Department of Epidemiology, Erasmus University Medical Center, 3015 CN Rotterdam, The Netherlands; 39 Department of Radiology, Erasmus University Medical Center, 3015 CN Rotterdam, The Netherlands; 40 Department of Clinical Chemistry, Fimlab Laboratories, and School of Medicine, University of Tampere, 33520 Tampere, Finland; 41 Department of Public Health, University of Helsinki, Helsinki, Finland; 42 Harvard Medical School, Boston, MA 02115 USA; 43 University Duisburg Essen, Essen, Germany; 44 Landspitali University Hospital, 101 Reykjavik, Iceland; 45 Department of Psychiatry, VU University Medical Centre, 1081 HL Amsterdam, The Netherlands; 46 Department of Psychiatry, Washington University School of Medicine, 660 South Euclid, CB 8134, St. Louis, MO 63110 USA; 47 University Medical Center Groningen, University of Groningen, Groningen, The Netherlands 9700RB; 48 MRC Functional Genomics Unit, Department of Physiology, Anatomy & Genetics, Oxford University, Oxford, UK; 49 Nuffield Department of Clinical Neuroscience, University of Oxford, Oxford, UK; 50 Oxford Headache Centre, John Radcliffe Hospital, Oxford, UK; 51 Max-Planck-Institute of Psychiatry, Munich, Germany; 52 Christian Albrechts University, Kiel, Germany; 53 Institute of Human Genetics, Helmholtz Center Munich, Neuherberg, Germany; 54 Department of General Practice and Primary Health Care, University of Helsinki and Helsinki University Hospital, Helsinki, Finland; 55 National Institute for Health and Welfare, Helsinki, Finland; 56 Institute of Clinical Medicine, University of Helsinki, Helsinki, Finland; 57 Department of Environmental Health, Harvard T.H. Chan School of Public Health, Boston, MA 02115 USA; 58 Department of Internal Medicine, Erasmus University Medical Center, 3015 CN Rotterdam, The Netherlands; 59 Department of Pain Management and Research, Oslo University Hospital, 0424 Oslo, Norway; 60 Medical Faculty, University of Oslo, 0318 Oslo, Norway; 61 Division of Mental Health, Norwegian Institute of Public Health, P.O. Box 4404 Nydalen, 0403 Oslo, Norway; 62 Kiel Pain and Headache Center, 24149 Kiel, Germany; 63 Danish Headache Center, Department of Neurology, Rigshospitalet, Glostrup Hospital, University of Copenhagen, Copenhagen, Denmark; 64 Institute of Biological Psychiatry, Mental Health Center Sct. Hans, University of Copenhagen, Roskilde, Denmark; 65 Institute Of Biological Psychiatry, MHC Sct. Hans, Mental Health Services Copenhagen, 2100 Copenhagen, Denmark; 66 Institute of Clinical Sciences, Faculty of Medicine and Health Sciences, University of Copenhagen, 2100 Copenhagen, Denmark; 67 iPSYCH - The Lundbeck Foundation’s Initiative for Integrative Psychiatric Research, 2100 Copenhagen, Denmark; 68 Department of Public Health, University of Helsinki, Helsinki, Finland; 69 Department of Health, National Institute for Health and Welfare, Helsinki, Finland; 70 Research Centre of Applied and Preventive Cardiovascular Medicine, University of Turku, 20521 Turku, Finland; 71 Department of Clinical Physiology and Nuclear Medicine, Turku University Hospital, 20521 Turku, Finland; 72 Department of Neurology, Erasmus University Medical Center, 3015 CN Rotterdam, The Netherlands; 73 Department of Epidemiology and Biostatistics, MRC Health Protection Agency (HPE) Centre for Environment and Health, School of Public Health, Imperial College London, London, W2 1PG UK; 74 Biocenter Oulu, University of Oulu, Box 5000, 90014 Oulu, Finland; 75 Unit of Primary Care, Oulu University Hospital, Box 10, FIN-90029 Oulu, Finland; 76 University Medical Center Hamburg Eppendorf, Institute of Human Genetics, 20246 Hamburg, Germany; 77 Population Health Research Institute, St George’s, University of London, Cranmer Terrace, London, SW17 0RE UK; 78 Munich Cluster for Systems Neurology (SyNergy), Munich, Germany; 79 Leiden University Medical Centre, Department of Human Genetics, PO Box 9600, 2300 RC Leiden, The Netherlands; 80 Faculty of Medicine, University of Iceland, 101 Reykjavik, Iceland; 81 Statistical and Genomic Epidemiology Laboratory, Institute of Health and Biomedical Innovation, Queensland University of Technology, 60 Musk Ave, Kelvin Grove, QLD 4059 Australia; 82 Department of Neurology, Massachusetts General Hospital, Boston, MA USA; 83 23andMe, Inc., 223 N Mathilda Ave, Sunnyvale, CA 94086 USA

**Keywords:** Genome-wide association studies, Hypertension, Migraine

## Abstract

Blood pressure (BP) was inconsistently associated with migraine and the mechanisms of BP-lowering medications in migraine prophylaxis are unknown. Leveraging large-scale summary statistics for migraine (*N*_cases_/*N*_controls_ = 59,674/316,078) and BP (*N* = 757,601), we find positive genetic correlations of migraine with diastolic BP (DBP, *r*_g_ = 0.11, *P* = 3.56 × 10^−06^) and systolic BP (SBP, *r*_g_ = 0.06, *P* = 0.01), but not pulse pressure (PP, *r*_g_ = −0.01, *P* = 0.75). Cross-trait meta-analysis reveals 14 shared loci (*P* ≤ 5 × 10^−08^), nine of which replicate (*P* < 0.05) in the UK Biobank. Five shared loci (*ITGB5*, *SMG6*, *ADRA2B*, *ANKDD1B*, and *KIAA0040*) are reinforced in gene-level analysis and highlight potential mechanisms involving vascular development, endothelial function and calcium homeostasis. Mendelian randomization reveals stronger instrumental estimates of DBP (OR [95% CI] = 1.20 [1.15–1.25]/10 mmHg; *P* = 5.57 × 10^−25^) on migraine than SBP (1.05 [1.03–1.07]/10 mmHg; *P* = 2.60 × 10^−07^) and a corresponding opposite effect for PP (0.92 [0.88–0.95]/10 mmHg; *P* = 3.65 × 10^−07^). These findings support a critical role of DBP in migraine susceptibility and shared biology underlying BP and migraine.

## Abstract (web summary)

The association between blood pressure (BP) and migraine is poorly understood. Here, the authors explore this relationship using summary-level GWAS data for BP and migraine. Cross-trait meta-analysis reveals shared loci between BP and migraine, while Mendelian randomization suggests that diastolic BP specifically plays a key role in migraine susceptibility.

## Introduction

Migraine is a chronic intermittent neurological disorder affecting up to 14.7% people worldwide and ranks as the second leading cause of disability, responsible for 5.6% of all years lived with disability^1^. The link between migraine and the vascular system has been substantiated by an array of physiologic and epidemiologic evidence, including migraine comorbidities with other vascular conditions including stroke, coronary artery disease (CAD)^2^. Recently, additional evidence for vascular involvement in migraine has emerged from genome-wide association studies (GWAS)^3^. Approximately, 40% (13 of 38) of the genome-wide significant GWAS loci for migraine map near genes with known or suspected vascular functions, including vascular development, endothelial structure, and smooth muscle function. Loci mapping to the *END1*/*PHACTR1*, *LRP1*, and *FHL5* genes in particular are shared by migraine and CAD or cervical artery dissection^4,5^.

Blood pressure (BP) has been associated not only with vascular disease but also with migraine^6^. In contrast to highly consistent associations of increased BP with increased susceptibility to vascular disease, associations of BP with migraine are not consistent^7^. For example, some studies have found associations between elevated systolic BP (SBP) or diastolic BP (DBP) and lower prevalence of migraine^8^, whereas some have found inverse associations only for SBP^9,10^. One study suggested that migraine was associated with higher DBP but lower SBP^11^. Still other reports focused on pulse pressure (PP), defined as the difference between SBP and DBP, consistently showed an inverse relationship between PP and migraine^9,11^. The relationship is further complicated by longitudinal studies suggesting that migraine may increase the risk of incident hypertension^12,13^, whereas BP has been found to be inversely related to onset of headache and migraine^14^. Regardless, BP-lowering medications notably provide prophylactic benefit for many migraineurs, and the choice of antihypertensive appears to be related to comorbidities, cost, availability, or side effect profile rather than the specific mechanism of BP-lowering^15,16^.

Recently developed but widely accepted genetic methods leveraging only GWAS summary statistics may be used to estimate global^17^ and local genetic correlation^18^ between BP measures (i.e. SBP, DBP, or PP) and migraine. Additional genetic methods using GWAS summary statistics, including cross-trait meta-analysis^19^ and transcriptome-wide association study (TWAS)^20^, may be used to identify specific shared genetic components and pathophysiology between BP and migraine. Finally, instrumental genetic analysis, i.e. Mendelian randomization (MR), may suggest causality and directionality of effects of BP on migraine, or the reverse, i.e. migraine influences on BP^21^. Therefore, in the current study, we leverage large-scale genetic summary-level data and the preceding genetic methods to gain insight into mechanistic links between BP and migraine.

Our analysis identifies positive overall genetic correlations of migraine with DBP and SBP, but not PP, and evidence of local genetic overlap with BP at certain previously identified migraine loci after accounting for multiple testing. Cross-trait meta-analysis reveals shared loci between BP and migraine, some of which are also reinforced in gene-level analysis highlighting potential shared biological mechanisms. In addition, MR shows stronger instrumental estimates of DBP on migraine than SBP. Our results suggest a critical role of DBP in migraine susceptibility and shared biological mechanisms between BP and migraine.

## Results

### Shared heritability between migraine and blood pressure

There was a positive overall genetic correlation of migraine with DBP (*r*_g_ = 0.11, Wald test *P* = 3.56 × 10^−06^) and SBP (*r*_g_ = 0.06, Wald test *P* = 0.01), but not PP (*r*_g_ = −0.01, Wald test *P* = 0.75) using linkage disequilibrium (LD) score regression (LDSC) (Table 1). When extended to the migraine subtypes: migraine with aura (MA) and migraine without aura (MO), DBP was consistently correlated with both MA (*r*_g_ = 0.17, Wald test *P* = 1.50 × 10^−03^) and MO (*r*_g_ = 0.14, Wald test *P* = 1.20 × 10^−03^), whereas SBP was only marginally correlated with MA (*r*_g_ = 0.10, Wald test *P* = 0.04). Findings for genetic covariance analyzer (GNOVA), which included SNPs with lower minor allele frequency (MAF) than LDSC, were similar with *r*_g_ of 0.12 (Wald test *P* = 3.45 × 10^−07^), 0.07 (Wald test *P* = 4.64 × 10^−03^), and 0.00 (Wald test *P* = 0.94) for DBP, SBP, and PP, respectively (Table 1). Partitioned genetic correlation did not reveal strong contrasts but suggested that shared effects were concentrated in some chromosomes with the strongest positive genetic correlation observed at chr22 (*r*_g_ = 0.47, Wald test *P* = 1.37 × 10^−04^) between migraine and DBP, and the strongest negative genetic correlation observed at chr19 (*r*_g_ = −0.32, Wald test *P* = 1.28 × 10^−03^) between migraine and PP (Supplementary Figs. 10–21).

**Table 1:** Genetic correlation between migraine and blood pressure.

Method	Trait 1	Trait 2	*r*_g_	P*	gcov	gcov_se
LDSC	Any migraine	DBP	0.11	3.56 × 10^−06^	0.018	0.009
		SBP	0.06	0.01	0.004	0.009
		PP	−0.01	0.75	−0.009	0.008
	Migraine with aura	DBP	0.17	1.50 × 10^−03^	−0.006	0.008
		SBP	0.10	0.04	−0.014	0.008
		PP	0.00	0.92	−0.015	0.007
	Migraine without aura	DBP	0.14	1.20 × 10^−03^	0.014	0.008
		SBP	0.03	0.43	0.010	0.008
		PP	−0.08	0.06	0.002	0.007
GNOVA	Any migraine	DBP	0.12	3.45 × 10^−07^	0.009	0.002
		SBP	0.07	4.64 × 10^−03^	0.005	0.002
		PP	0.00	0.94	0.000	0.002
	Migraine with aura	DBP	0.15	1.90 × 10^−05^	0.008	0.002
		SBP	0.10	2.57 × 10^−03^	0.006	0.002
		PP	0.03	0.33	0.002	0.002
	Migraine without aura	DBP	0.13	1.86 × 10^−04^	0.008	0.002
		SBP	−0.02	0.66	−0.001	0.002
		PP	−0.12	2.12 × 10^−04^	−0.006	0.002

*r*_*g*_ Genetic correlation, *gcov* genetic covariance, *gcov_se* standard error of genetic covariance, *LDSC* LD score regression, *GNOVA* genetic covariance analyzer, *DBP* diastolic blood pressure, *SBP* systolic blood pressure, *PP* pulse pressure.

*P-value was calculated for the genetic correlation in LDSC and for the genetic covariance in GNOVA, *P*-values are based on two-sided Wald test.

**Fig. 1:**
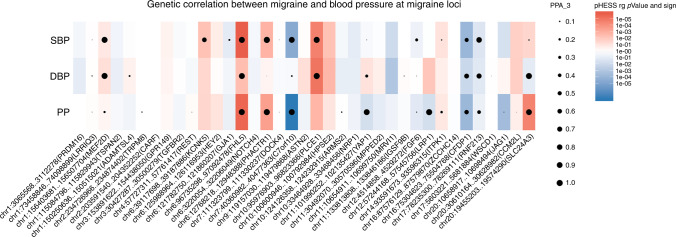
Local genetic correlation between migraine and BP traits at reported migraine loci using ρ-HESS and GWAS-PW. Colors represent the significance level of local genetic correlation between migraine and blood pressure (BP) traits (DBP, SBP, and PP) using ρ-HESS (*P*_ρ-HESS_ based on Wald test), red for positive genetic correlation and blue for negative genetic correlation at the corresponding locus. Dots represent the estimated posterior probability (PPA_3) that genetic associations with migraine and BP traits (DBP, SBP, and PP) co-localize at the corresponding locus, larger size indicate larger posterior probability. Significant local genetic correlation between BP traits and migraine was observed at three regions: harboring gene *FHL5*, *C7orf10*, and *PLCE1*, after controlling for multiple testing (*P*_ρ-HESS_ < 0.05/1703, see details in Supplementary Table 1) and with high estimated posterior probability (PPA_3 > 0.9, see details in Supplementary Table 2).

The local genomic regions around individual migraine loci from GWAS showed signals of genetic overlap with BP (Fig. 1). Accounting for multiple testing, there was genome-wide significant local genetic correlation between migraine and BP at three regions (chr6: 94441175..97093511 harboring previous migraine locus *FHL5*; chr7: 39862670..42001811 harboring previous migraine locus *C7orf10*; and chr10: 95396368..96221243 harboring previous migraine locus *PLCE1*) using heritability estimation from summary statistics (ρ-HESS) (Fig. 1 and Supplementary Table 1, *P* < 0.05/1703). The genetic correlation between migraine and SBP was negative in the chromosome 7 region despite being positive across the whole genome (Fig. 1). For PP, although the overall genome-wide genetic correlation with migraine was null, there were significant local genetic correlations at chromosome 6 (Wald test *P* = 3.20 × 10^−06^) and 7 (Wald test *P* = 3.98 × 10^−08^), which were also significantly correlated for the other BP measures. Results were consistent for these regions with the alternative pairwise traits analysis of GWAS (GWAS-PW) approach (i.e. PPA_3 > 0.9, Fig. 1 and Supplementary Table 2).

Taken together, although the overall genetic correlations between BP traits and migraine were relatively modest compared to more closely related phenotypes, e.g. among psychiatric disorders (*r*_g_ ~ 0.6) or between lipids and CAD (*r*_g_ ~ 0.25)^22^, they nevertheless indicate potential shared genetic etiologies, especially at certain chromosomes or regions, and are therefore worthy of additional investigation into potential mechanisms using cross-trait analysis and expression-trait analysis.

### Cross-trait meta-analysis of migraine with BP measurements

We conducted cross-trait meta-analysis to identify individual SNPs that may share association with BP and migraine using the Cross Phenotype Association (CPASSOC) package. Thirty-three independent loci reached genome-wide significance for combined statistics (*P*_CPASSOC_ ≤ 5 × 10^−08^) and suggestive trait-specific significance (*P*_GWAS_ ≤ 1 × 10^−05^) for migraine and at least one BP measurement (Supplementary Tables 3–5), 19 of which were previously reported migraine loci, including *PHACTR1*, *LRP1, FHL5*, *C7orf10*, *MPPED2*, *CFDP1*, and *SLC24A3*. Nine of the remaining 14 shared loci (Table 2) were replicated at nominal significance level in the independent migraine association study using UK Biobank data, and 10 of them were also related with broadly-defined headache (*P* < 0.05, Supplementary Table 6).

**Table 2:** Candidate migraine loci from cross-trait meta-analysis between migraine and blood pressure using CPASSOC.

Trait 1	Trait 2	SNP	POS	A1	A2	MAF	Trait 1		Trait 2		*P*_CPASSOC_	Genes
							BETA	*P*	BETA	*P*		
Any migraine	DBP	rs72663521	chr1p34.3	A	G	0.19	0.04	1.94 × 10^−06^	0.13	1.42 × 10^−08^	2.22 × 10^−12^	*BMP8A, KIAA0754, MACF1, PABPC4, PPIEL, SNORA55*
		rs3766694	chr1q25.1	T	C	0.39	−0.03	1.26 × 10^−06^	0.11	4.07 × 10^−10^	3.17 × 10^−14^	*KIAA0040*
		rs62155750	chr2q11.1	A	G	0.31	0.04	4.42 × 10^−07^	−0.22	8.27 × 10^−29^	5.42 × 10^−34^	*ADRA2B, ARID5A, ASTL, CIAO1, CNNM4, DUSP2, FAHD2A, FAHD2CP, FER1L5, GPAT2, ITPRIPL1, KANSL3, KCNIP3, LINC00342, LMAN2L, NCAPH, NEURL3, PROM2, SNRNP200, STARD7, STARD7-AS1, TMEM127, TRIM43, TRIM43B*
		rs6438857	chr3q21.2	T	C	0.43	−0.03	8.92 × 10^−07^	0.15	1.50 × 10^−17^	2.64 × 10^−22^	*ITGB5, KALRN, MUC13, UMPS*
		rs6881648	chr5q13.3	A	C	0.37	−0.04	4.76 × 10^−07^	−0.17	5.06 × 10^−21^	3.43 × 10^−26^	*ANKDD1B, ANKRD31, COL4A3BP, HMGCR, POC5, POLK*
		rs1271309	chr12q24.31	A	G	0.17	−0.04	8.56 × 10^−06^	−0.20	1.45 × 10^−16^	2.04 × 10^−20^	*FAM101A, MIR6880, NCOR2, ZNF664-FAM101A*
		rs13260	chr13q34	T	G	0.09	0.06	6.60 × 10^−07^	−0.20	1.66 × 10^−10^	8.69 × 10^−15^	*COL4A1*
		rs8008129	chr14q23.1	T	C	0.34	0.03	3.91 × 10^−06^	0.09	1.37 × 10^−06^	7.22 × 10^−10^	*ACTR10, ARID4A, FLJ31306, PSMA3*
		rs28451064	chr21q22.11	A	G	0.13	−0.06	2.69 × 10^−07^	0.13	1.54 × 10^−06^	1.96 × 10^−10^	*Intergenic near MRPS6*
	SBP	rs6438857	chr3q21.2	T	C	0.43	−0.03	8.92 × 10^−07^	0.27	3.13 × 10^−19^	1.77 × 10^−23^	*ITGB5, KALRN, MUC13, UMPS*
		rs1048483	chr17p13.3	T	C	0.49	−0.03	1.31 × 10^−06^	−0.30	6.49 × 10^−23^	9.29 × 10^−27^	*DPH1, HIC1, LOC101927839, MIR132, MIR212, OVCA2, RTN4RL1, SMG6, SRR, TSR1*
		rs8080108	chr17q21.32	T	C	0.30	−0.03	3.74 × 10^−06^	−0.30	3.15 × 10^−20^	1.22 × 10^−23^	*ABI3, FLJ40194, LOC102724596, MIR6129, PHB, PHOSPHO1, ZNF652*
	PP	rs6438857	chr3q21.2	T	C	0.43	−0.03	8.92 × 10^−07^	0.13	9.00 × 10^−10^	2.55 × 10^−14^	*ITGB5, MUC13, UMPS*
		rs974819	chr11q22.3	T	C	0.29	−0.03	1.00 × 10^−05^	0.11	4.15 × 10^−07^	1.67 × 10^−10^	*Intergenic*
		rs12875271	chr13q34	A	G	0.09	−0.06	5.15 × 10^−07^	−0.19	1.18 × 10^−07^	6.29 × 10^−12^	*COL4A1*
		rs28577186	chr16p13.3	A	G	0.35	−0.04	1.44 × 10^−06^	−0.14	8.39 × 10^−10^	3.77 × 10^−14^	*C16orf96, CDIP1, CORO7, CORO7-PAM16, DNAJA3, HMOX2, MGRN1, NMRAL1, PAM16, UBALD1, VASN*
		rs1048483	chr17p13.3	T	C	0.49	−0.03	1.31 × 10^−06^	−0.20	1.47 × 10^−22^	5.13 × 10^−28^	*DPH1, HIC1, LOC101927839, MIR132, MIR212, OVCA2, RTN4RL1, SMG6, SRR, TSR1*
		rs1800470	chr19q13.2	A	G	0.40	0.04	4.97 × 10^−07^	−0.15	1.76 × 10^−12^	1.49 × 10^−17^	*ATP5SL, B3GNT8, B9D2, BCKDHA, EXOSC5, TGFB1, TMEM91*
		rs9982601	chr21q22.11	T	C	0.13	−0.05	1.78 × 10^−07^	−0.21	7.51 × 10^−12^	3.38 × 10^−17^	*Intergenic near MRPS6*

Position is under build 37/hg19.

All these loci were candidate genes to migraine with genome-wide significant (*P* < 5 × 10^−8^) for cross-trait meta-analysis (using heterogonous version of CPASSOC, SHet) and *P* < 1 × 10^−5^ for single trait GWAS, *P-*values are based on S_Het_ statistic.

*POS* position, *MAF* minor allele frequency, *DBP* diastolic blood pressure, *SBP* systolic blood pressure, *PP* pulse pressure.

Among the candidate migraine loci, lead SNP *rs62155750* was most significant (chr2q11.1, *P*_CPASSOC_ = 5.42 × 10^−34^ for DBP based on S_Het_ statistic). *Rs62155750* was a significant expression quantitative trait locus (eQTL) for its nearby gene *ADRA2B* (Supplementary Table 7), encoding the subtype B of the α2-adrenergic receptor that regulates neurotransmitter release from sympathetic nerves and adrenergic neurons in the central nervous system^23^. Interestingly, this locus was related to migraine (*P* = 0.02 based on S_Het_ statistic) but not broadly defined headache (*P* = 0.55 based on S_Het_ statistic) in the replication dataset (Supplementary Table 6). The second strongest signal overall was lead SNP *rs1048483* (at chr17p13.3) that was associated with both SBP (*P*_CPASSOC_ = 9.29 × 10^−27^ based on S_Het_ statistic) and PP (*P*_CPASSOC_ = 5.13 × 10^−28^ based on S_Het_ statistic. *Rs1048483* mapped to *SMG6* that encodes a nonsense-mediated mRNA decay factor, and is a significant eQTL for the nearby gene *SSR* (Serine Racemase, Supplementary Table 8), which is responsible for transforming l‐serine to d‐serine, a key co-agonist with glutamate at *N*‐methyl‐d‐aspartate (NMDA) receptors^24^. Lead SNP *rs6438857* (at chr3q21.2, *P*_CPASSOC_ = 2.64 × 10^−22^, 1.77 × 10^−23^, 2.55 × 10^−14^ for DBP, SBP, and PP, respectively based on S_Het_ statistic) implicating *ITGB5* was the only locus that was shared between migraine and all the three BP measurements. *ITGB5* encodes a beta subunit of integrin (integrin alpha-V/beta-5), which is a member of integrin family of heterodimeric transmembrane cell surface receptors and has a role in vascular permeability induced by vascular endothelial growth factor (VEGF) in the systemic circulation^25^. *COL4A1* at chr13q34 was shared between migraine and DBP (lead SNP *rs13260*, *P*_CPASSOC_ = 8.69 × 10^−15^ based on S_Het_ statistic) as well as PP (lead SNP *rs12875271*, *P*_CPASSOC_ = 6.29 × 10^−12^ based on S_Het_ statistic). *COL4A1* encodes a type IV collagen alpha protein, and *COL4A1* mutations may present with small vessel disease and stroke, both of which also have migraine as a clinical feature^26,27^. *TGFB1* at chr19q13.2 (lead SNP *rs1800470*, *P*_CPASSOC_ = 1.49 × 10^−17^ based on S_Het_ statistic) was shared between migraine and PP alone and encodes a transforming growth factor-beta 1 protein (TGF-β1) family member.

Cross-trait meta-analysis between migraine subtypes (MA and MO) and BP showed that previous reported migraine loci, including *PHACTR1*, *LRP1*, and *FHL5*, were shared between both migraine subtypes and BP while locus *rs4141663* implicating *ITGB5* was genome-wide significant in cross-trait meta-analysis between MO and BP measurements, but not MA (Supplementary Tables 9–14).

### Transcriptome-wide association studies

**Fig. 2:**
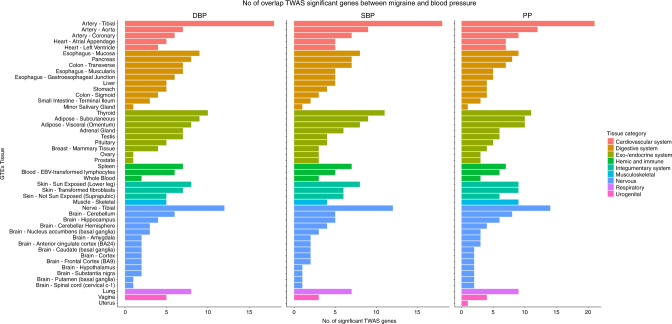
Number of shared TWAS significant genes between migraine and BP traits across 48 GTEx tissues (version 7). The *X* axis shows the count of genes from tissues in the GTEx database meeting significance thresholds for multiple testing for migraine and for each of the BP measures as indicated. The *Y* axis lists GTEx tissues. Colors represent different tissue categories. The null hypothesis of TWAS is no expression-trait association (or genetic correlation between expression and a trait) conditional on the observed GWAS statistics at the corresponding locus. The total number of TWAS gene-tissue pairs being tested is 206,397 across 48 GTEx tissues. TWAS transcriptome-wide association studies, BP blood pressure, DBP diastolic blood pressure, SBP systolic blood pressure, PP pulse pressure, No. number.

We performed TWAS to identify gene-level genetic overlap between BP and migraine. There were 76 TWAS genes that were transcriptome-wide significant for both migraine and at least one BP trait, most of which were identified from gene expression in tissues of cardiovascular and nervous system (Fig. 2). Restricting this list to shared genes with independent signals (see Methods), we identified 23 genes that were TWAS significant for both migraine and at least one of the BP traits from tissues including artery, nerve, skin, esophagus mucosa, and whole blood (Supplementary Tables 15–17), among which 12 were migraine candidate genes. Five of these 12 genes were also identified by the cross-trait meta-analysis (*ITGB5*, *SMG6*, *ADRA2B*, *ANKDD1B*, and *KIAA0040*). *ITGB5*, *SMG6*, and *ADRA2B* are described above. Data on *ANKDD1B* and *KIAA0040* were limited, but *ANKDD1B* was previously suggested to have a shared role between migraine and major depressive disorder (MDD)^28^. Other gene-level genetic overlap between migraine and BP included genes (*CISD2*, *DMPK*, and *C12orf5*) that were related to regulation of calcium homeostasis and reactive oxygen species (ROS)^29,30^. TWAS genes with independent effects shared by subtypes of migraine and BP were consistent with findings for overall migraine at *ITGB5*, while identifying additional associations at *HMOX2* for MA and BP, and *HVCN1* and *MANBA* for MO and BP (Supplementary Figs. 22–27, Supplementary Tables 18–23).

### Instrumental variable analysis

Finally, we used bi-directional MR instrumental analysis to develop evidence for causality in the relationship between BP and migraine. Genetically instrumented elevated DBP and SBP, and decreased PP were associated with increased risk of having migraine with odds ratios (OR) of 1.20 (95% confidence interval [CI] = 1.15–1.25; Wald test *P* = 5.01 × 10^−24^) and 1.05 (95% CI = 1.03–1.07; Wald test *P* = 2.34 × 10^−06^) per 10 mmHg increment of DBP and SBP, and 1.09 (95% CI = 1.05–1.14; Wald test *P* = 3.29 × 10^−06^) per 10 mmHg decrement of PP (Table 3). There were also significant instrumental variable estimates from migraine to BP. Reverse MR showed significant negative instrumental effects per doubling odds of migraine on SBP (estimate = 0.67 mmHg decrement, Wald test *P* = 1.01 × 10^−10^) and PP (estimate = 0.55 mmHg decrement, Wald test *P* = 3.21 × 10^−15^), but not DBP (estimate = 0.08 mmHg decrement, Wald test *P* = 0.45). All heterogeneity *P*-values were non-significant (*P*_HEIDI_ > 0.01) indicating at worst only subtle heterogeneity among retained instruments. In conditional analysis to distinguish effects mediated by DBP from those mediated by SBP, there was an increase in the instrumental association of high DBP on migraine with conditioning on SBP (OR [95% CI] = 1.38 [1.30–1.46], Wald test *P* = 4.16 × 10^−37^), while an opposite effect of high SBP on migraine with conditioning on DBP (OR [95% CI] = 0.86 [0.83–0.90], Wald test *P* = 2.08 × 10^−22^). The diverging instrumental effects of DBP and SBP on migraine were also supported by restricting analysis to SNP instruments that were non-significant (*P* > 0.05) for one measure but highly significant (*P* < 1 × 10^−5^) for the other (Supplementary Fig. 28). For significance thresholds of *P* < 5 × 10^−8^ or smaller, the instrumental effects of DBP and SBP for migraine were associated respectively with increased and decreased migraine susceptibility. The instrumental variable analysis revealed consistent associations of elevated DBP and decreased PP with MO (OR [95% CI] = 1.34 [1.21–1.47], Wald test *P* = 1.24 × 10^−09^, OR [95% CI] = 1.16 [1.05, 1.28], Wald test *P* = 5.80 × 10^−03^, respectively), whereas no significant association was observed for MA after controlling for multiple testing (Table 3). Sensitivity analysis for the main MR analysis using inverse-variance weighted (IVW), weighted median, simple median, and MR-Egger procedures suggested there was no systematic bias due to pleiotropy (Supplementary Table 24), and MR-Steiger results showed that all the causal estimates were oriented in the intended direction (all *P*_MR-Steiger_ < 0.05). Taken together, the instrumental analyses suggest a potential causal role of elevated DBP on migraine susceptibility, whereas conditional on DBP, SBP may be causally protective. These relationships are also reflected in a potential inverse causal relationship between PP and migraine.

**Table 3:** Bi-directional instrumental estimates between migraine and blood pressure using GSMR.

Exposure	Outcome	Covariates	Direction	Instrumental estimates^a^	se	*P*-Bonferroni
DBP	Any migraine	—	Forward	0.18	0.02	5.01 × 10^−24^
			Reverse	−0.11	0.07	0.45
	MA	—	Forward	0.12	0.05	0.18
			Reverse^b^			
	MO	—	Forward	0.29	0.05	1.24 × 10^−09^
			Reverse^b^			
SBP	Any migraine	—	Forward	0.05	0.01	2.34 × 10^−06^
			Reverse	−0.97	0.15	1.01 × 10^−10^
	MA	—	Forward	0.04	0.03	1.00
			Reverse^b^			
	MO	—	Forward	0.06	0.03	0.36
			Reverse^b^			
PP	Any migraine	—	Forward	−0.09	0.02	3.29 × 10^−06^
			Reverse	−0.79	0.10	3.21 × 10^−15^
	MA	—	Forward	−0.06	0.05	1.00
			Reverse^b^			
	MO	—	Forward	−0.15	0.05	5.80 × 10^−03^
			Reverse^b^			
Conditional GSMR^c^						
DBP	Any migraine	SBP	Forward	0.32	0.03	4.16 × 10^−37^
SBP	Any migraine	DBP	Forward	−0.15	0.02	2.08 × 10^−22^

*GSMR* Generalized summary-data-based Mendelian randomization, *se* standard error, *DBP* diastolic blood pressure, *SBP* systolic blood pressure, *PP* pulse pressure, *MA* migraine with aura, *MO* migraine without aura.

*P-*values are based on two-sided Wald test and used Bonferroni correction.

^a^The instrumental estimate is corresponding to 10 mmHg increment of blood pressure for the forward direction.

^b^Too few instruments to conduct reverse GSMR for migraine with aura and without aura (number of genome-wide significant index SNPs <10).

^c^Conditional GSMR was performed by conditioning the exposure on the corresponding covariates (using mtCOJO, https://cnsgenomics.com/software/gcta/#mtCOJO and then using the conditioned summary statistics to infer the instrumental estimates from the exposure to the outcome.

We also applied MR to explore the potential role of causality in anti-hypertensives for migraine prophylaxis effect by only examining lead variants in targets of BP-lowering medications (i.e. beta blocker: *ADRB1*, ACE inhibitor: *ACE*, calcium channel blockers: *CACNB2*, *CACNA1D*, and *CACNA1C*)^31^. Instrumental associations at these SNPs were directionally consistent with the preceding findings but none was significant alone or in combination (all *P* > 0.05), nor was any SNP strongly associated with migraine alone (all *P* > 0.01) (Supplementary Table 25).

When applied to two cardiovascular comorbidities of migraine, stroke and CAD, the instrumental methods suggested a prominent role for SBP rather than DBP (Table 4). Although both SBP and DBP were strongly associated with all stroke subtypes in the primary analysis, conditioning by SPB attenuated the DBP effect for all stroke subtypes except for large artery stroke (LAS), for which there was a significant inverse DBP association. After conditioning on DBP, SBP remained significantly associated with any stroke, ischemic stroke, large artery stroke, and small vessel stroke. Similarly, after conditioning on DBP, SBP was positively associated with CAD, but DBP conditioned on SBP had an inverse association. In sensitivity analysis restricted to SNP instruments that were significant (*P* < 1 × 10^−5^) for one BP trait but non-significant for the other (*P* > 0.05), SBP was inferred to have stronger effects than DBP on CAD and LAS, for which the effect of DBP was protective as observed in the conditional analysis (Supplementary Fig. 29). For the other stroke outcomes, effects of SBP were stronger than or comparable to effects of DBP, especially when using stronger SNP instruments.

**Table 4:** Instrumental estimates between blood pressure and cardiovascular diseases (stroke and CAD) using GSMR.

Exposure	Outcome	Direction	GSMR^a^			Conditional GSMR^b^			
			Instrumental estimates	se	*P*	Covariates	Instrumental estimates	se	*P*
DBP	AS	Forward	0.50	0.03	1.82E-47	SBP	−0.04	0.03	0.24
SBP		Forward	0.31	0.02	9.49E-61	DBP	0.13	0.02	1.04E-12
DBP	IS	Forward	0.49	0.04	1.36E-38	SBP	−0.1	0.03	3.05E-03
SBP		Forward	0.30	0.02	2.36E-51	DBP	0.19	0.02	4.10E-22
DBP	LAS	Forward	0.59	0.09	9.90E-11	SBP	−0.67	0.08	1.10E-15
SBP		Forward	0.56	0.05	6.28E-30	DBP	0.49	0.05	2.70E-25
DBP	CES	Forward	0.27	0.07	9.67E-05	SBP	0.01	0.06	0.84
SBP		Forward	0.17	0.04	4.36E-06	DBP	0.06	0.04	0.10
DBP	SVS	Forward	0.75	0.09	2.11E-18	SBP	0.12	0.08	0.12
SBP		Forward	0.39	0.05	2.62E-17	DBP	0.17	0.04	6.65E-05
DBP	CAD	Forward	0.59	0.04	3.69E-58	SBP	−0.19	0.03	2.83E-08
SBP		Forward	0.34	0.02	3.87E-71	DBP	0.2	0.02	6.56E-26

*GSMR* Generalized summary-data-based Mendelian randomization, *se* standard error, *DBP* diastolic blood pressure, *SBP* systolic blood pressure, *AS* any stroke, *IS* ischemic stroke, *LAS* large artery stroke, *CES* cardioembolic stroke, *SVD* small vessel stroke, *CAD* coronary artery disease.

*P-*values are based on two-sided Wald test.

^a^The instrumental estimate is corresponding to 10 mmHg increment of blood pressure on the corresponding outcome.

^b^Conditional GSMR was performed by conditioning the exposure on the corresponding covariates (using mtCOJO, https://cnsgenomics.com/software/gcta/#mtCOJO) and then use the conditioned summary statistics to infer the instrumental estimates from the exposure to the outcome.

## Discussion

The conclusions from our genetic analyses were highly consistent and generally support observational associations of positive correlation between BP and migraine^32^ but also qualify these associations in important ways. We find the strongest association between elevated DBP and increased migraine susceptibility. Weaker genetic relationships of elevated SBP with migraine were largely explained by effects on DBP, and conditional on DBP, genetically determined SBP was inversely related to migraine susceptibility. The latter relationship was supported by SNP instruments exclusively associated with SBP and the reverse direction instrumental variable analysis. Consistent with distinct effects of SBP and DBP, greater genetically determined PP was strongly associated with less susceptibility to migraine in the instrumental variable analysis. Because we leveraged germline genetic variation as instrumental variables from large independent studies, our causal estimates will be less affected by reverse causation and possibly also selection bias than inference about relationships between BP and migraine from observational epidemiology^33,34^. In fact, the findings from genetics are concordant with at least one of the prior observational studies^8^.

Meanwhile, 9 replicating SNPs from cross-trait association analysis as well as 12 genes from TWAS of both migraine and BP suggested potential functions relevant to migraine. The five loci identified in both SNP and TWAS analysis revealed potential shared biological mechanisms in migraine and BP regulation involving vascular development and endothelial function, neurogenic inflammation, calcium homeostasis through proteins encoded by *ITGB5*, *SMG6*, *ADRA2B*, *ANKDD1B*, and *KIAA0040* and, in particular, functions of the α2-adrenergic receptor type B encoded by *ADRA2B*. Neurotransmitters, such as glutamate, serotonin (5-HT), dopamine (DA), noradrenalin (NE), substance P, and calcitonin gene-related peptide (CGRP), have all been identified as contributing causally to migraine^35^, as well as potential therapeutic targets^36,37^, and all are related with the α2-adrenergic receptor regulation^38^. Therefore, our results support the role of α2-adrenergic receptor in migraine mechanisms.

In contrast to the results for the genetic effects of DBP and PP on migraine, the genetic association between BP and cardiovascular events was driven by SBP, consistent with the results from observational studies^39^. This suggests that different mechanisms may underlie BP associations with migraine compared to CVD. Thus, observational associations of migraine with cardiovascular events likely do not involve BP-based etiology in a trivial way, a conclusion further supported by the larger MR effects of BP on cardiovascular events compared to the MR effects of BP on migraine. However, it is also possible that potential genetic heterogeneity in migraine or misclassification due to changes in migraine presentation over time may have attenuated the MR association between BP and migraine^3^.

This study comprehensively investigates the genetic-based association between migraine and BP. The main strengths of our study include large-scale genetic data (sample size up to 757,601), independent replication of migraine candidate loci from cross-trait meta-analysis, the use of multiple MR sensitivity analysis for outliers, horizontal pleiotropy, and reverse causation, and the use of exclusive SNP instruments for DBP or SBP that were significant for one trait (*P* < 1.00 × 10^−5^) but non-significant (*P* > 0.05) for the other. However, we acknowledge limitations. First, our conclusions are limited to a general susceptibility of migraine and its major subtypes MA and MO but may not extend to different migraine traits over time or forms of migraine that may not arise from the common, population-based genetic susceptibilities implicit in our datasets, e.g. familial forms of migraine. Second, although the instrumental analysis focused on genetic variation in targets of BP-lowering medications (beta blocker, ACE inhibitor, and calcium channel blocker) was not significant, it may also have been underpowered. Based on the combined effects of SNPs in these genes on BP, we estimated there was only <50% power at nominal significance to detect such instrumental effects on migraine in our datasets^40^. Third, although our analysis points to tissues and genes relevant to migraine susceptibility and BP, more work is needed to identify individual cell types and more detailed molecular mechanisms with the goal of developing potential therapeutic strategies.

Nevertheless, the findings further our understanding of the long-standing debate about the role of BP in migraine susceptibility, reveal the prominent genetic-based role of DBP in migraine susceptibility, and identify shared genetic components including *ADRA2B*, all of which may provide insight into future migraine therapies.

## Methods

### Summary statistics from GWAS for migraine and blood pressure

We used the most recent GWAS summary-level data from International Headache Genetics Consortium (IHGC) for migraine (any migraine and two subtypes of migraine: migraine with aura [MA] and migraine without aura [MO]) and from the International Consortium of Blood Pressure-Genome Wide Association Studies (ICBP) and UK Biobank (UKB) for three BP traits (SBP, DBP, and PP)^3,41^. The migraine meta-analysis summary statistics combined 59,674 cases and 316,078 controls from 22 cohort level GWASs^3^, whereas the BP meta-analysis summary statistics combined 757,601 participants from the UKB (*N* = 458,577) and ICBP (*N* = 299,024 across 77 cohorts)^41^. In the original GWASs, migraine and its two sub-forms (MA and MO) were defined by diagnostic criteria from the International Headache Society and the summary statistics were adjusted for age, sex, and principle components where applicable in each sub-cohort^3^, whereas BP summary statistics (including three traits: SBP, DBP, and PP) were adjusted for age, age^2^, sex, and body mass index (BMI) in the parent study, and all sub-cohorts corrected for hypertension treatment (+15/10 mmHg in the presence of any hypertensive medication)^41^. All of the participants were of European descent with only a small fraction of overlapping samples (*N* = 39,199, proportion of overlapping samples is ~10% for migraine summary statistics, and ~5% for BP summary statistics) between migraine and BP traits. Analysis in the current study was restricted to SNPs, at most ~7 million, which were common to GWASs for migraine and the BP traits. To compare the instrumental effects of BP traits on migraine and two migraine cardiovascular comorbidities, coronary artery disease (CAD) and stroke, we used publicly available GWAS summary statistics from European descent individuals for CAD and stroke from CARDIoGRAM and MEGASTROKE, respectively^42,43^. To minimize the bias from overlapping samples when conducting the instrumental analyses of BP with CAD and stroke, we used BP GWAS summary statistics (*N* = 361,194) from the UK Biobank, which is publicly available at http://www.nealelab.is/uk-biobank/^44^. All participants provided written informed consent to each of the sub-cohort of the consortium.

### Genetic correlation analysis

To evaluate genetic correlation between migraine and BP, we used conventional cross-trait linkage disequilibrium (LD) score regression (LDSC)^17^ and the more recent genetic covariance analyzer (GNOVA)^45^. For LDSC, we used precomputed LD-scores derived from ~1.2 million common- and well-imputed SNPs in European populations as represented in the Hapmap3 reference panel excluding the HLA region^17^. With GNOVA, which is potentially more powerful than LDSC^45^, we estimated the genetic correlation across ~5 million well-imputed SNPs in the 1000 Genomes Project and partitioned the estimates among categories of SNPs defined by 11 functional categories^46^, quartiles of MAF, and regions implicated in transcription for seven broadly-defined tissue types^45^. Both LDSC and GNOVA controlled for potential overlapping samples between each pair of traits^17,45^.

### Local genetic correlation

We estimated local genetic correlations between migraine and BP traits in 1703 pre-specified LD-independent segments with both ρ-HESS^18^ and GWAS-PW^47^. Both methods are designed to identify small contiguous regions of the genome in which the genetic associations with two traits are locally concordant. However, they use different approaches. ρ-HESS quantifies the local genetic covariance (and correlation) and *P*-values (*P*_ρ-HESS_) between pairs of traits at local regions^18^, whereas GWAS-PW uses a Bayesian framework to estimate the posterior probability (PPA_3) that genetic associations with the two traits co-localize using priors that are learned from the data^47^. BP and migraine were considered to have genetic correlation at local region if *P*_ρ-HESS_ was significant after correcting for multiple testing (*P*_ρ-HESS_ < 0.05/1703) and PPA_3 from GWAS-PW was larger than 0.9.

### Cross-trait meta-analysis between migraine and BP traits

We conducted pairwise cross-trait meta-analysis using Cross Phenotype Association (CPASSOC)^19^ through the statistic *S*_Het_ that implements a sample size-weighted, fixed effect meta-analysis of the association statistics from the individual traits while modeling genetic covariance from all sources. In these analyses, we used total sample size values directly from the summary statistics file for BP and an average effective sample size for migraine^48^. The cross-trait meta-analysis was not inflated by observing a mean ratio of (LDSC intercept-1)/(mean(*χ*^2^) − 1) at 0.05 (Supplementary Figs. 1–9). Replication of migraine candidate associations from CPASSOC was performed using an independent dataset from UK Biobank (using data from data field 20002 and 6159 for migraine and recent headache, respectively, see details in Supplementary Note 1).

### Transcriptome-wide association studies

To identify genes whose expression pattern across tissues implicates etiology or biological mechanisms shared by migraine and the BP measures, we performed TWAS^49^. With TWAS, we compared gene-based models of genetic effects on tissue-specific gene expression from GTEx v.7 for migraine and the BP measures from the GWAS summary statistics to estimate strength of association between concordant gene-based genetic influences on gene expression on migraine or BP. In total, we performed 48 TWASs for each trait, one tissue–trait pair at a time. The null hypothesis of TWAS is no expression–trait association (or genetic correlation between expression and a trait) conditional on the observed GWAS statistics at the locus. In practice, a permutation test based on 1000 resampling iterations was run for each TWAS gene to ensure that the TWAS false positive rate was well controlled^49^. We applied Bonferroni correction to identify significant expression-trait associations adjusted for multiple comparisons for all gene–tissue pairs tested for each trait (~200,000 gene-tissue pairs in total, significant expression–trait associations were defined as *P*_Bonferroni_ < 0.05), and then identified genes that had Bonferroni significant associations for both migraine and BP. We further tested for conditional relationships among the shared genes to identify an independent set of gene-based genetic models using an extension of TWAS that leverages previous methods for joint/conditional tests of SNPs using summary statistics^20^ (Supplementary Note 2).

### Generalized summary-data-based Mendelian randomization

To examine evidence for potential causal relationships between migraine and BP, we conducted instrumental variable analysis using bi-directional MR implemented in generalized summary-data-based Mendelian randomization (GSMR)^21^. GSMR applies strict criteria to select independent SNP instruments and extends conventional MR by accounting for the sampling variance in the genetic effects on both exposure (*b*_*zx*_) and outcome (*b*_*zy*_) in estimating the instrumental effect. Further, as pleiotropy is an important potential confounder that could bias the estimates and possibly result in an inflated test-statistic in MR, we used heterogeneity criteria in HEIDI (heterogeneity in dependent instruments, *P*_HEIDI_ < 0.01) in the GSMR package to exclude likely pleiotropic SNPs from the analysis. To evaluate separate effects of SBP and DBP on migraine, we performed conditional instrumental analysis using mtCOJO (multi-trait-based conditional and joint analysis), also within GSMR, with a two-step procedure requiring only the GWAS summary statistics^21^. SNP effects on SBP (*y*) were adjusted for effects on DBP (*x*) (or vice-versa) (i.e. *b*_*xy*_ obtained from GSMR) in step 1, and then the adjusted instruments were used to derive the conditional instrumental estimate in step 2. *P*-values were corrected for multiple testing using Bonferroni criteria. We conducted sensitivity analyses using conventional inverse-variance weighted (IVW) MR, weighted median, simple median, MR-egger (Egger regression), and MR-Steiger (Supplementary Note 3). As migraine is a binary variable, we interpreted the reverse causal estimates as the average change in BP per doubling (twofold increase) in the odds of migraine, which could be obtained by multiplying the reverse causal estimate by 0.693 (log_e_2)^50^.

### Reporting summary

Further information on research design is available in the Nature Research Reporting Summary linked to this article.

## Supplementary information

Supplementary Information

Peer Review File

Reporting Summary

## Data Availability

Summary-level data for CAD (CARDIoGRAM), Stroke (MEGASTROKE), and BP (International Consortium of Blood Pressure genetics [ICBP] and the UK Biobank [UKB]) are publicly available at: http://www.cardiogramplusc4d.org/data-downloads/ and http://www.megastroke.org/download.html; and http://www.nealelab.is/uk-biobank/. Summary-level data (*P* < 1 × 10^−5^) from International Headache Genetics Consortium (IHGC) for migraine are available here: http://www.headachegenetics.org/content/datasets-and-cohorts. Individual level data from the UK Biobank (UKB) are available upon application: https://www.ukbiobank.ac.uk/.
