# Supplementary material for: A genome-wide cross-phenotype meta-analysis of the association of blood pressure with migraine
Source: Nat Commun. 2020 Jul 6;11:3368. doi: 10.1038/s41467-020-17002-0 (PMC7338361; doi:10.1038/s41467-020-17002-0)
Supplement: Supplementary file 1 — Supplementary Information [file 41467_2020_17002_MOESM1_ESM.pdf]

Supplementary Information

A genome-wide cross-phenotype meta-analysis of the association of blood pressure with migraine

Guo et. al.

Contents

Supplementary Notes ... 4
Supplementary Note 1 ... 4
Supplementary Note 2 ... 4
Supplementary Note 3 ... 4
Supplementary Table 1 ... 6
Supplementary Table 2 ... 7
Supplementary Table 3 ... 8
Supplementary Table 4 ... 10
Supplementary Table 5 ... 11
Supplementary Table 6 ... 13
Supplementary Table 7 ... 14
Supplementary Table 8 ... 15
Supplementary Table 9 ... 16
Supplementary Table 10 ... 17
Supplementary Table 11 ... 18
Supplementary Table 12 ... 19
Supplementary Table 13 ... 20
Supplementary Table 14 ... 21
Supplementary Table 15 ... 22
Supplementary Table 16 ... 27
Supplementary Table 17 ... 30
Supplementary Table 18 ... 33
Supplementary Table 19 ... 35
Supplementary Table 20 ... 37
Supplementary Table 21 ... 39
Supplementary Table 22 ... 41
Supplementary Table 23 ... 43
Supplementary Table 24 ... 45
Supplementary Table 25 ... 47
Supplementary Figure 1 ... 48
Supplementary Figure 2 ... 48
Supplementary Figure 3 ... 48
Supplementary Figure 4 ... 49
Supplementary Figure 5 ... 49

Supplementary Figure 6......49
Supplementary Figure 7......50
Supplementary Figure 8......50
Supplementary Figure 9......50
Supplementary Figure 10......51
Supplementary Figure 11......51
Supplementary Figure 12......52
Supplementary Figure 13......52
Supplementary Figure 14......53
Supplementary Figure 15......53
Supplementary Figure 16......54
Supplementary Figure 17......54
Supplementary Figure 18......55
Supplementary Figure 19......55
Supplementary Figure 20......56
Supplementary Figure 21......56
Supplementary Figure 22......57
Supplementary Figure 23......58
Supplementary Figure 24......59
Supplementary Figure 25......60
Supplementary Figure 26......61
Supplementary Figure 27......62
Supplementary Figure 28......63
Supplementary Figure 29......64
Author list for International Headache Genetics Consortium (IHGC)......65
Author list for MEGASTROKE Consortium......67
Author list for CARDIoGRAM Consortium......74
Author list for ICBP Consortium......76
References......82

Supplementary Notes

Supplementary Note 1

Replication of novel migraine candidate associations from CPASSOC

Replication of novel migraine candidate associations from CPASSOC was performed using logistic models adjusting for sex, age, genotype array, recruitment center, and the first 10 genetic principal components (PCs) in an independent data set from UK Biobank (restricted to unrelated European ancestry) for migraine (N_{cases} / $N_{controls}$= 10,005 / 267,330 from data field 20002 with excluding participants with broad headache [defined by data field 6159] in migraine controls to avoid bias due to high correlation and heterogeneity between them) and broad headache (N_{cases} / $N_{controls}$= 67,052 / 269,883, data field: 6159).

Supplementary Note 2

Transcriptome-wide association studies (TWAS)

We performed TWAS to identify genes whose expression pattern across tissues implicates etiology or biological mechanisms shared by migraine and the BP measures. To do this, we first merged the summary statistics for each trait to a subset of SNPs with Hapmap3 SNPs excluding the HLA region [1], and then integrated this pruned GWAS summary data (~1.2 million SNPs) for migraine or each of BP traits (SBP, DBP, and PP) with LD-adjusted weight vector learned from the gene expression data to perform TWASs across 48 tissues from GTEx v.7 [2] using available software and precompiled data resources (see URLs) [1,3]. The strength of association between concordant gene-based genetic influences on gene expression on migraine or BP was given by Z_{TWAS} as described [3]. The number of available gene-based models for gene expression from GTEx ranged from 1,604 for brain substantia nigra to 9,826 for thyroid. We also conducted conditional TWAS analysis among the shared genes by leveraging previous methods for joint/conditional tests of SNPs using summary statistics [4,5] to identify an independent set of TWAS genes. The correlation/LD matrix for this step was computed using Pearson correlation of genetic effects on gene expression predicted for the 1000 genomes genotypes as described[4] across all pairwise genes shared by migraine and the BP measures. With this correlation matrix, we determined separate sets of independent gene-based models of genetic

effects for migraine and BP measures separately, deriving a final set of shared gene-based models as the overlap across tissues.

Supplementary Note 3

Mendelian Randomization (MR) sensitivity analyses

We conducted sensitivity analyses for the primary MR analysis using conventional inverse-variance weighted (IVW) MR, weighted median, simple median, MR-egger (Egger regression) implemented in R package "MendelianRandomization" [6]. We also applied MR-Steiger with the R package "TwoSampleMR" to assure that the causal direction between the hypothesized exposure and outcome was correctly assigned [7].

Supplementary Table 1 Genome-wide significant regions from local genetic correlation analysis (*ρ*-HESS) between migraine and blood pressure (*P*<0.05/1703)

Trait 1	Trait 2	chr	start	end	num_snp	local_rhog	var	se	z	*p*
Migraine	DBP	10	95396368	96221243	1073	0.00022339	2.55E-09	5.05E-05	4.42	9.85E-06
	SBP	6	94441175	97093511	4084	0.00022129	2.14E-09	4.63E-05	4.78	1.74E-06
		7	39862670	42001811	2725	-0.0001779	1.77E-09	4.21E-05	-4.23	2.35E-05
		10	95396368	96221243	1073	0.00022985	2.98E-09	5.46E-05	4.21	2.56E-05
	PP	6	94441175	97093511	4084	0.00021535	2.14E-09	4.62E-05	4.66	3.20E-06
		7	39862670	42001811	2725	-0.00025176	2.10E-09	4.58E-05	-5.49	3.98E-08

Abbreviations: chr: chromosome; num_snp: number of SNPs in region; local_rhog: local genetic covariance in the corresponding region; se: standard error; DBP: diastolic blood pressure; SBP: systolic blood pressure; PP: pulse pressure.

Supplementary Table 2 Genome-wide significant regions from local genetic correlation analysis (GWAS-PW) between migraine and blood pressure (PPA_3>0.90)

Trait 1	Trait 2	NSNP	chr	st	sp	PPA_1	PPA_2	PPA_3	PPA_4
Migraine	DBP	4201	1	156336687	158027330	4.79E-09	1.68E-18	0.97	0.03
		5308	1	175089963	177433277	1.48E-10	0.0006	0.96	0.04
		3197	2	95358799	98994973	6.01E-28	0.00022	0.93	0.06
		3876	3	123518258	124935910	1.24E-17	0.00055	0.96	0.04
		6708	6	94441595	97093400	4.02E-11	4.37E-23	0.96	0.04
		1695	10	95397754	96220615	2.31E-56	1.11E-11	0.99	0.01
		4293	10	96222131	97820339	2.39E-26	9.16E-05	0.94	0.06
		849	12	8429979	9031328	1.60E-17	0.00255	0.98	0.01
		4914	13	109815112	111231864	3.33E-11	0.00067	0.95	0.04
		4992	20	18972387	20959637	3.89E-10	5.13E-16	0.98	0.02
	SBP	4201	1	156336687	158027330	1.01E-08	3.26E-22	0.95	0.05
		3876	3	123518258	124935910	7.80E-19	1.14E-07	0.91	0.09
		3426	6	11791351	13209144	3.58E-18	8.69E-23	1.00	0.00
		6708	6	94441595	97093400	2.01E-20	4.02E-27	0.96	0.04
		4050	7	39863558	42001171	2.62E-16	3.54E-16	0.93	0.07
		1695	10	95397754	96220615	8.33E-48	2.43E-15	0.98	0.02
		849	12	8429979	9031328	1.05E-10	7.13E-07	0.95	0.05
	PP	3876	3	123518258	124935910	2.19E-10	0.00112	0.90	0.10
		3426	6	11791351	13209144	2.70E-36	5.29E-19	1.00	0.00
		6708	6	94441595	97093400	1.55E-17	2.31E-23	0.98	0.02
		4050	7	39863558	42001171	8.26E-27	2.29E-12	0.95	0.05
		8343	11	101331132	103958766	5.52E-12	1.80E-05	0.95	0.05
		2853	12	55665948	57548466	1.59E-09	8.35E-45	0.99	0.01
		5202	12	89684199	92065305	1.55E-59	0.00734	0.91	0.08
		4914	13	109815112	111231864	3.49E-08	0.00027	0.98	0.02
		4992	20	18972387	20959637	1.13E-34	2.37E-15	0.91	0.09
		3322	21	35226082	36523905	6.58E-12	9.37E-05	0.94	0.06

Abbreviations: chr: chromosome; NSNP: number of SNPs in region; st:start; ed:end; PPA_1: posterior probability for model 1 (association only to phenotype 1); PPA_2: posterior probability for model 2 (association only to phenotype 2); PPA_3: posterior probability for model 3 (shared association to both phenotypes); PPA_4: posterior probability for model 4 (two distinct associations, one to each phenotype); DBP: diastolic blood pressure; SBP: systolic blood pressure; PP: pulse pressure.

Supplementary Table 3 **Genome-wide significant loci shared between migraine and diastolic blood pressure (DBP) from cross-trait meta-analysis using CPASSOC (P_{meta}≤5×10^{-08})**

SNP	CHR	BP	POS	A1	A2	A1FREQ	Migraine			DBP			P_{meta}	Genes within Ranges
							BETA	SE	*P*	BETA	SE	*P*		
rs1271309	12	124820705	chr12:124771217..124843104	A	G	0.17	-0.04	0.01	8.56E-06	-0.20	0.02	1.45E-16	2.04E-20	FAM101A,MIR6880,NCOR2,ZNF664-FAM101A
rs13260	13	110802123	chr13:110788441..110818102	T	G	0.09	0.06	0.01	6.60E-07	-0.20	0.03	1.66E-10	8.69E-15	COL4A1
rs1861881	9	119312256	chr9:119157030..119486923	T	G	0.31	0.03	0.01	6.60E-06	0.12	0.02	6.49E-10	1.67E-13	ASTN2,LOC100128505,PAPPA,PAPPA-AS1,TRIM32
rs2004295	6	39154866	chr6:39093606..39187886	A	G	0.27	-0.04	0.01	4.60E-08	-0.13	0.02	4.79E-11	3.34E-16	KCNK5
rs2052692	11	10667641	chr11:10579687..10736660	A	G	0.33	-0.05	0.01	3.38E-13	0.17	0.02	4.28E-21	4.50E-31	LYVE1,MRVI1,MRVI1-AS1
rs2274319	1	156450873	chr1:156406853..156507670	T	C	0.35	0.07	0.01	1.43E-21	0.10	0.02	1.65E-07	3.64E-21	IQGAP3,MEF2D
rs2500281	1	3220382	chr1:3210950..3229134	A	G	0.28	-0.04	0.01	5.43E-06	0.10	0.02	9.73E-07	6.21E-10	PRDM16
rs28451064	21	35593827	chr21:35593827..35682958	A	G	0.13	-0.06	0.01	2.69E-07	0.13	0.03	1.54E-06	1.96E-10	near MRPS6
rs28455731	6	121846038	chr6:121782750..121848841	T	G	0.16	0.05	0.01	7.29E-09	-0.11	0.02	3.37E-06	7.51E-11	near GJA1
rs2971603	6	97035418	chr6:96732257..97081432	T	C	0.23	0.09	0.01	2.83E-27	0.13	0.02	1.23E-09	5.67E-27	FHL5,UFL1
rs3766694	1	175143207	chr1:175118895..175162805	T	C	0.61	-0.03	0.01	1.26E-06	0.11	0.02	4.07E-10	3.17E-14	KIAA0040
rs3851738	16	75387533	chr16:75305331..75516534	C	G	0.43	-0.04	0.01	1.34E-09	0.13	0.02	7.09E-13	3.12E-19	CFDP1,CHST6,TMEM170A
rs4814864	20	19469817	chr20:19455203..19573475	C	G	0.26	0.07	0.01	2.19E-19	-0.11	0.02	8.04E-09	2.05E-19	SLC24A3
rs57866767	10	96023077	chr10:95952031..96993955	T	C	0.44	0.05	0.01	2.32E-14	0.28	0.02	2.86E-57	7.42E-71	C10orf129,CYP2C8,CYP2C9,CYP2C18,CYP2C19,HELLS,NOC3L,PLCE1,PLCE1-AS1,TBC1D12
rs62155750	2	96491456	chr2:95931218..97428320	A	G	0.31	0.04	0.01	4.42E-07	-0.22	0.02	8.27E-29	5.42E-34	ADRA2B,ARID5A,ASTL,CIAO1,CNNM4,DUSP2,FAHD2A,FAHD2CP,FER1L5,GPAT2,ITPRIPL1,KANSL3,KCNIP3,LINC00342,LMAN2L,NCAPH,NEURL3,PROM2,SNRNP200,STARD7
rs6438857	3	124557643	chr3:124331571..124648820	T	C	0.57	-0.03	0.01	8.92E-07	0.15	0.02	1.50E-17	2.64E-22	ITGB5,KALRN,MUC13,UMPS

rs6881648	5	74991849	chr5:74328643..75146256	A	C	0.37	-0.04	0.01	4.76E-07	-0.17	0.02	5.06E-21	3.43E-26	ANKDD1B,ANKRD31,COL4A3BP,HMGCR,POC5,POLK
rs72663521	1	40005903	chr1:39551488..40088043	A	G	0.19	0.04	0.01	1.94E-06	0.13	0.02	1.42E-08	2.22E-12	BMP8A,KIAA0754,MACF1,PABPC4,PPIEL,SNORA55
rs8008129	14	58829032	chr14:58668484..58853140	T	C	0.34	0.03	0.01	3.91E-06	0.09	0.02	1.37E-06	7.22E-10	ACTR10,ARID4A,FLJ31306,PSMA3
rs978851	10	100660301	chr10:100601902..101139262	T	C	0.29	0.04	0.01	7.76E-06	0.09	0.02	5.50E-06	5.51E-09	CNNM1,HPSE2

Abbreviations: CHR: chromosome; BP: physical position; POS: position; A1FREQ: A1 allele frequency; DBP: diastolic blood pressure.

Position is under build 37/hg19.

All these loci were genome-wide significant ($P<5\times10^{-8}$) for cross-trait meta-analysis (using heterogonous version of CPASSOC, SHet) and $P<1\times10^{-5}$ for single trait GWAS.

Supplementary Table 4 Genome-wide significant loci shared between migraine and systolic blood pressure (SBP) from cross-trait meta-analysis using CPASSOC (P_{meta}≤5×10^{-08})

SNP	CHR	BP	POS	A1	A2	A1FREQ1	Migraine			SBP			P_{meta}	Genes within Ranges
							BETA	SE	*P*	BETA	SE	*P*		
rs1048483	17	1966457	chr17:1926337..2229067	T	C	0.49	-0.03	0.01	1.31E-06	-0.30	0.03	6.49E-23	9.29E-27	DPH1,HIC1,LOC101927839,MIR132,MIR212,OVCA2,RTN4RL1,SMG6,SRR,TSR1
rs12202891	6	12768218	chr6:12768218..12768218	T	C	0.17	-0.07	0.01	2.16E-10	-0.21	0.04	5.81E-07	2.83E-12	PHACTR1
rs12947578	17	78246734	chr17:78235300..78285371	T	C	0.43	0.05	0.01	1.11E-09	-0.16	0.03	1.01E-06	8.76E-12	RNF213
rs1332844	6	12889004	chr6:12762814..12982423	T	C	0.36	-0.05	0.01	1.48E-13	-0.21	0.03	1.06E-11	1.19E-19	PHACTR1
rs17171710	7	40440233	chr7:40120135..40479852	T	C	0.11	0.09	0.01	4.98E-15	-0.41	0.05	1.13E-16	1.17E-26	CDK13,MPLKIP,SUGCT (C7orf10)
rs2052692	11	10667641	chr11:10579687..10736660	A	G	0.33	-0.05	0.01	3.38E-13	0.19	0.03	2.13E-09	6.23E-17	LYVE1,MRVI1,MRVI1-AS1
rs2274319	1	156450873	chr1:156406853..156507670	T	C	0.35	0.07	0.01	1.43E-21	0.16	0.03	2.47E-07	1.70E-20	IQGAP3,MEF2D
rs2983896	6	97029871	chr6:96682566..97081432	A	G	0.22	0.09	0.01	4.79E-27	0.34	0.04	1.61E-20	1.53E-37	FHL5,UFL1
rs4814864	20	19469817	chr20:19455203..19573475	C	G	0.26	0.07	0.01	2.19E-19	0.18	0.03	1.44E-07	2.36E-18	SLC24A3
rs4888408	16	75432824	chr16:75240935..75516534	A	G	0.43	-0.04	0.01	3.02E-09	0.37	0.03	1.42E-32	4.24E-39	BCAR1,CFDP1,CHST6,CTRB1,CTRB2,LOC100506281,TMEM170A
rs546240	11	30533622	chr11:30493671..30570681	T	C	0.38	-0.03	0.01	3.55E-06	0.15	0.03	5.76E-07	3.84E-10	MPPED2
rs57866767	10	96023077	chr10:95952031..96797500	T	C	0.44	0.05	0.01	2.32E-14	0.45	0.03	1.14E-49	2.82E-60	CYP2C8,CYP2C9,CYP2C18,CYP2C19,HELLS,NOC3L,PLCE1,PLCE1-AS1,TBC1D12
rs6438857	3	124557643	chr3:124331571..124648820	T	C	0.57	-0.03	0.01	8.92E-07	0.27	0.03	3.13E-19	1.77E-23	ITGB5,KALRN,MUC13,UMPS
rs733701	6	39171862	chr6:39115413..39187886	T	C	0.26	0.06	0.01	1.26E-12	0.19	0.03	3.70E-08	7.53E-15	KCNK5
rs8080108	17	47513711	chr17:47289566..47516523	T	C	0.30	-0.03	0.01	3.74E-06	-0.30	0.03	3.15E-20	1.22E-23	ABI3,FLJ40194,LOC102724596,MIR6129,PHB,PHOSPHO1,ZNF652
rs978851	10	100660301	chr10:100601902..101139262	T	C	0.29	0.04	0.01	7.76E-06	0.15	0.03	8.10E-06	1.53E-08	CNNM1,HPSE2

Abbreviations: CHR: chromosome; BP: physical position; POS: position; A1FREQ: A1 allele frequency; SBP: systolic blood pressure.

Position is under build 37/hg19.

All these loci were genome-wide significant ($P<5\times10^{-8}$) for cross-trait meta-analysis (using heterogonous version of CPASSOC, SHet) and $P<1\times10^{-5}$ for single trait GWAS.

Supplementary Table 5 Genome-wide significant loci shared between migraine and pulse pressure (PP) from cross-trait meta-analysis using CPASSOC (P_{meta}≤5×10^{-08})

SNP	CHR	BP	POS	A1	A2	A1FREQ1	Migraine			PP			P_{meta}	Genes within Ranges
							BETA	SE	*P*	BETA	SE	*P*		
rs1048483	17	1966457	chr17:1926337..2229067	T	C	0.49	-0.03	0.01	1.31E-06	-0.20	0.02	1.47E-22	5.13E-28	DPH1,HIC1,LOC101927839,MIR132,MIR212,OVCA2,RTN4RL1,SMG6,SRR,TSR1
rs11172113	12	57527283	chr12:57256380..57545756	T	C	0.42	0.10	0.01	5.64E-49	-0.12	0.02	3.02E-08	1.71E-50	GPR182,LRP1,MYO1A,NAB2,RDH16,SDR9C7,STAT6,TAC3,TMEM194A,ZBTB39
rs12202891	6	12768218	chr6:12768218..13022728	T	C	0.17	-0.07	0.01	2.16E-10	-0.21	0.03	5.19E-14	9.87E-22	PHACTR1
rs12875271	13	110792743	chr13:110788441..110804809	A	G	0.09	-0.06	0.01	5.15E-07	-0.19	0.04	1.18E-07	6.29E-12	COL4A1
rs1332844	6	12889004	chr6:12762814..13098302	T	C	0.36	-0.05	0.01	1.48E-13	-0.20	0.02	6.95E-22	3.11E-33	PHACTR1
rs17171710	7	40440233	chr7:40000089..40479852	T	C	0.11	0.09	0.01	4.98E-15	-0.37	0.03	7.39E-28	3.10E-41	CDK13,MPLKIP,SUGCT (C7orf10)
rs1800470	19	41858921	chr19:41851509..41946095	A	G	0.40	0.04	0.01	4.97E-07	-0.15	0.02	1.76E-12	1.49E-17	ATP5SL,B3GNT8,B9D2,BCKDHA,EXOSC5,TGFB1,TMEM91
rs2282652	11	102097315	chr11:101915105..102125714	T	C	0.33	-0.04	0.01	6.47E-08	0.15	0.02	1.17E-11	2.46E-17	C11orf70,YAP1
rs28577186	16	4488191	chr16:4399075..4687695	A	G	0.35	-0.04	0.01	1.44E-06	-0.14	0.02	8.39E-10	3.77E-14	C16orf96,CDIP1,CORO7,CORO7-PAM16,DNAJA3,HMOX2,MGRN1,NMRAL1,PAM16,UBALD1,VASN
rs2983896	6	97029871	chr6:96732257..97081432	A	G	0.22	0.09	0.01	4.79E-27	0.21	0.02	1.26E-17	2.74E-36	FHL5,UFL1
rs4888408	16	75432824	chr16:75242729..75516534	A	G	0.43	-0.04	0.01	3.02E-09	0.25	0.02	1.01E-32	1.31E-41	BCAR1,CFDP1,CHST6,CTRB1,LOC100506281,TMEM170A
rs546240	11	30533622	chr11:30449319..30570681	T	C	0.38	-0.03	0.01	3.55E-06	0.13	0.02	4.47E-10	3.07E-14	MPPED2
rs57866767	10	96023077	chr10:95973747..96784778	T	C	0.44	0.05	0.01	2.32E-14	0.18	0.02	4.88E-18	2.35E-29	CYP2C9,CYP2C18,CYP2C19,HELLS,NOC3L,PLCE1,PLCE1-AS1,TBC1D12
rs6035355	20	19465089	chr20:19455203..19614751	A	T	0.28	0.07	0.01	4.86E-18	0.29	0.02	1.25E-36	9.26E-54	SLC24A3
rs6438857	3	124557643	chr3:124441501..124648820	T	C	0.57	-0.03	0.01	8.92E-07	0.13	0.02	9.00E-10	2.55E-14	ITGB5,MUC13,UMPS
rs6515020	20	19504904	chr20:19460032..19587337	T	C	0.09	-0.06	0.01	1.12E-07	-0.21	0.03	5.79E-10	4.03E-15	SLC24A3

rs6693567	1	150510660	chr1:150250534..150832108	T	C	0.27	-0.04	0.01	1.21E-08	0.14	0.02	4.09E-09	8.51E-15	ADAMTSL4,ADAMTSL4-AS1,ARNT,C1orf54,CIART,CTSK,CTSS,ECM1,ENSA,GOLPH3L,HORMAD1,LINC00568,MCL1,MIR4257,MIR6878,MRPS21,PRPF3,RPRD2,TARS2
rs934287	2	203708307	chr2:203320423..204367311	A	G	0.17	-0.04	0.01	3.66E-06	0.16	0.03	1.18E-09	1.01E-13	ABI2,BMPR2,CARF,CYP20A1,FAM117B,ICA1L,NBEAL1,RAPH1,WDR12
rs974819	11	103660567	chr11:103599717..103718660	T	C	0.29	-0.03	0.01	1.00E-05	0.11	0.02	4.15E-07	1.67E-10	Intergenic
rs9982601	21	35599128	chr21:35566316..35716371	T	C	0.13	-0.05	0.01	1.78E-07	-0.21	0.03	7.51E-12	3.38E-17	Intergenic

Abbreviations: CHR: chromosome; BP: physical position; POS: position; A1FREQ: A1 allele frequency; PP: pulse pressure.

Position is under build 37/hg19.

All these loci were genome-wide significant ($P<5\times10^{-8}$) for cross-trait meta-analysis (using heterogonous version of CPASSOC, SHet) and $P<1\times10^{-5}$ for single trait GWAS.

Supplementary Table 6 replication of migraine novel loci from CPASSOC meta-analysis by using logistic regression in UK Biobank

Chr	SNP	BP	A1	A2	MAF	Migraine (N=277,335)			Headache (N=336,935)		
						BETA	SE	*P*	BETA	SE	*P*
1	rs72663521	40005903	G	A	0.18	0.004	0.019	0.82	-0.020	0.008	0.01
1	rs3766694	175143207	T	C	0.38	-0.041	0.015	6.07E-03	-0.028	0.006	1.54E-05
2	rs62155750	96491456	A	G	0.31	0.036	0.016	0.02	0.004	0.007	0.55
3	rs6438857	124557643	T	C	0.42	-0.028	0.015	0.05	-0.015	0.006	0.02
5	rs6881648	74991849	A	C	0.37	-0.066	0.015	9.39E-06	-0.029	0.006	6.86E-06
11	rs974819	103660567	T	C	0.29	-0.009	0.016	0.56	0.008	0.007	0.23
12	rs1271309	124820705	A	G	0.16	-0.062	0.020	1.78E-03	-0.030	0.008	3.27E-04
13	rs12875271	110792743	A	G	0.09	-0.064	0.024	8.59E-03	-0.024	0.011	0.02
13	rs13260	110802123	G	T	0.09	-0.063	0.025	0.01	-0.022	0.011	0.04
14	rs8008129	58829032	T	C	0.36	0.041	0.015	6.93E-03	0.011	0.006	0.08
16	rs28577186	4488191	G	A	0.33	0.028	0.015	0.07	-0.005	0.007	0.44
17	rs1048483	1966457	C	T	0.49	0.019	0.015	0.20	0.014	0.006	0.03
17	rs8080108	47513711	T	C	0.31	-0.054	0.016	6.43E-04	-0.030	0.007	9.98E-06
19	rs1800470	41858921	G	A	0.37	-0.049	0.015	1.19E-03	-0.024	0.006	1.59E-04
21	rs28451064	35593827	G	A	0.13	0.117	0.023	2.96E-07	0.032	0.009	6.37E-04
21	rs9982601	35599128	C	T	0.13	0.092	0.022	3.82E-05	0.025	0.009	6.14E-03

Abbreviations: Chr: chromosome; BP: physical position; MAF: minor allele frequency; SE: standard error.

Position is under build 37/hg19.

Model adjusted for sex, age, genotype array, recruitment center, and 10 PCs.

Supplementary Table 7 eQTL results for rs62155750 at *ADRA2B* from GTEx (version 7 release)

Gencode Id	Gene Symbol	Variant Id	SNP Id	P-Value	NES	Tissue
ENSG00000222040.3	ADRA2B	2_96491456_A_G_b37	rs62155750	1.40E-14	0.46	Thyroid
ENSG00000222040.3	ADRA2B	2_96491456_A_G_b37	rs62155750	7.90E-11	0.41	Nerve - Tibial
ENSG00000222040.3	ADRA2B	2_96491456_A_G_b37	rs62155750	2.10E-08	0.33	Colon - Transverse
ENSG00000222040.3	ADRA2B	2_96491456_A_G_b37	rs62155750	2.90E-08	0.29	Adipose - Subcutaneous
ENSG00000222040.3	ADRA2B	2_96491456_A_G_b37	rs62155750	3.30E-08	0.34	Stomach
ENSG00000222040.3	ADRA2B	2_96491456_A_G_b37	rs62155750	4.1E-06	0.31	Adipose - Visceral (Omentum)
ENSG00000222040.3	ADRA2B	2_96491456_A_G_b37	rs62155750	4.8E-06	0.26	Esophagus - Muscularis
ENSG00000222040.3	ADRA2B	2_96491456_A_G_b37	rs62155750	0.000038	0.22	Muscle - Skeletal

Abbreviations: NES=normalized effect size.

Supplementary Table 8 **eQTL results for rs1048483 from GTEx (version 7 release)**

Gencode Id	Gene Symbol	Variant Id	SNP Id	*P*-Value	NES	Tissue
ENSG00000167720.8	SRR	17_1966457_C_T_b37	rs1048483	2.30E-17	-0.45	Artery - Tibial
ENSG00000167720.8	SRR	17_1966457_C_T_b37	rs1048483	4.20E-17	-0.41	Muscle - Skeletal
ENSG00000167720.8	SRR	17_1966457_C_T_b37	rs1048483	5.50E-16	-0.46	Esophagus - Mucosa
ENSG00000167720.8	SRR	17_1966457_C_T_b37	rs1048483	2.10E-14	-0.34	Lung
ENSG00000167720.8	SRR	17_1966457_C_T_b37	rs1048483	2.70E-14	-0.36	Thyroid
ENSG00000167720.8	SRR	17_1966457_C_T_b37	rs1048483	6.00E-14	-0.43	Skin - Sun Exposed (Lower leg)
ENSG00000167720.8	SRR	17_1966457_C_T_b37	rs1048483	1.10E-13	-0.35	Esophagus - Muscularis
ENSG00000167720.8	SRR	17_1966457_C_T_b37	rs1048483	3.40E-12	-0.37	Adipose - Subcutaneous
ENSG00000167720.8	SRR	17_1966457_C_T_b37	rs1048483	9.90E-12	-0.4	Heart - Atrial Appendage
ENSG00000167720.8	SRR	17_1966457_C_T_b37	rs1048483	1.40E-11	-0.37	Adipose - Visceral (Omentum)
ENSG00000167720.8	SRR	17_1966457_C_T_b37	rs1048483	1.40E-10	-0.4	Colon - Transverse
ENSG00000167720.8	SRR	17_1966457_C_T_b37	rs1048483	2.60E-10	-0.44	Colon - Sigmoid
ENSG00000167720.8	SRR	17_1966457_C_T_b37	rs1048483	3.60E-10	-0.36	Whole Blood
ENSG00000167720.8	SRR	17_1966457_C_T_b37	rs1048483	2.00E-09	-0.36	Skin - Not Sun Exposed (Suprapubic)
ENSG00000167720.8	SRR	17_1966457_C_T_b37	rs1048483	2.20E-09	-0.46	Stomach
ENSG00000167720.8	SRR	17_1966457_C_T_b37	rs1048483	2.80E-09	-0.38	Breast - Mammary Tissue
ENSG00000167720.8	SRR	17_1966457_C_T_b37	rs1048483	3.50E-09	-0.36	Nerve - Tibial
ENSG00000167720.8	SRR	17_1966457_C_T_b37	rs1048483	1.20E-08	-0.33	Esophagus - Gastroesophageal Junction
ENSG00000167720.8	SRR	17_1966457_C_T_b37	rs1048483	1.20E-07	-0.29	Artery - Aorta
ENSG00000167720.8	SRR	17_1966457_C_T_b37	rs1048483	1.60E-07	-0.46	Pancreas
ENSG00000167720.8	SRR	17_1966457_C_T_b37	rs1048483	1.70E-07	-0.28	Cells - Transformed fibroblasts
ENSG00000167720.8	SRR	17_1966457_C_T_b37	rs1048483	3.60E-07	-0.45	Brain - Cerebellum
ENSG00000167720.8	SRR	17_1966457_C_T_b37	rs1048483	4.60E-07	-0.29	Heart - Left Ventricle
ENSG00000167720.8	SRR	17_1966457_C_T_b37	rs1048483	5.00E-07	-0.34	Adrenal Gland
ENSG00000167720.8	SRR	17_1966457_C_T_b37	rs1048483	8.20E-07	-0.49	Brain - Anterior cingulate cortex (BA24)
ENSG00000167720.8	SRR	17_1966457_C_T_b37	rs1048483	9.70E-07	-0.47	Brain - Cortex
ENSG00000185924.6	RTN4RL1	17_1966457_C_T_b37	rs1048483	0.0000013	0.17	Adipose - Visceral (Omentum)

Abbreviations: NES=normalized effect size.

Supplementary Table 9 Genome-wide significant loci shared between migraine with aura and diastolic blood pressure (DBP) from cross-trait meta-analysis using CPASSOC (P_{meta}≤5×10^{-08})

SNP	CHR	BP	POS	A1	A2	A1FREQ1	Migraine with aura			SBP			P_{meta}	Genes within Ranges
							BETA	SE	*P*	BETA	SE	*P*		
rs3798293	6	97033370	chr6:96841762..97067047	A	G	0.22	-0.12	0.03	6.63E-07	-0.13	0.02	2.70E-10	1.04E-12	[**FHL5**,UFL1]
rs2052692	11	10667641	chr11:10579687..10736660	A	G	0.65	-0.11	0.02	3.09E-06	0.17	0.02	4.28E-21	1.09E-24	[LYVE1,**MRVI1**, MRVI1-AS1]

Abbreviations: CHR: chromosome; BP: physical position; POS: position; A1FREQ: A1 allele frequency; DBP: dystolic blood pressure.

Position is under build 37/hg19.

All these loci were genome-wide significant (*P*<5×10^{-8}) for cross-trait meta-analysis (using heterogonous version of CPASSOC, SHet) and *P*<1×10^{-5} for single trait GWAS.

Supplementary Table 10 Genome-wide significant loci shared between migraine with aura and systolic blood pressure (SBP) from cross-trait meta-analysis using CPASSOC (P_{meta}≤5×10^{-08})

SNP	CHR	BP	POS	A1	A2	A1FREQ1	Migraine with aura			SBP			P_{meta}	Genes within Ranges
							BETA	SE	*P*	BETA	SE	*P*		
rs2983896	6	97029871	chr6:96767685..97081432	A	G	0.22	0.12	0.03	8.85E-07	0.34	0.04	1.61E-20	2.72E-26	[**FHL5**,UFL1]
rs2052692	11	10667641	chr11:10579687..10736660	A	G	0.65	-0.11	0.02	3.09E-06	0.19	0.03	2.13E-09	3.02E-12	[LYVE1,**MRVI1**,MRVI1-AS1]

Abbreviations: CHR: chromosome; BP: physical position; POS: position; A1FREQ: A1 allele frequency; SBP: systolic blood pressure.

Position is under build 37/hg19.

All these loci were genome-wide significant (P<5×10^{-8}) for cross-trait meta-analysis (using heterogonous version of CPASSOC, SHet) and P<1×10^{-5} for single trait GWAS.

Supplementary Table 11 Genome-wide significant loci shared between migraine with aura and pulse pressure (PP) from cross-trait meta-analysis using CPASSOC (P_{meta}≤5×10^{-08})

SNP	CHR	BP	POS	A1	A2	A1FREQ1	Migraine with aura			PP			P_{meta}	Genes within Ranges
							BETA	SE	*P*	BETA	SE	*P*		
rs2983896	6	97029871	chr6:96767685..97070190	A	G	0.22	0.12	0.03	8.85E-07	0.21	0.02	1.26E-17	8.11E-22	[**FHL5**,UFL1]
rs11172113	12	57527283	chr12:57502981..57540751	T	C	0.45	0.10	0.02	1.11E-06	-0.12	0.02	3.02E-08	1.58E-10	[**LRP1**,STAT6]

Abbreviations: CHR: chromosome; BP: physical position; POS: position; A1FREQ: A1 allele frequency; PP: pulse pressure.

Position is under build 37/hg19.

All these loci were genome-wide significant (P<5×10^{-8}) for cross-trait meta-analysis (using heterogonous version of CPASSOC, SHet) and P<1×10^{-5} for single trait GWAS.

Supplementary Table 12 Genome-wide significant loci shared between migraine without aura and diastolic blood pressure (DBP) from cross-trait meta-analysis using CPASSOC ($P_{meta} \leq 5\times10^{-08}$)

SNP	CHR	BP	POS	A1	A2	A1FREQ1	Migraine without aura			DBP			P_{meta}	Genes within Ranges
							BETA	SE	*P*	BETA	SE	*P*		
rs2274319	1	156450873	chr1:156450873..156500604	T	C	0.64	0.09	0.02	2.45E-06	0.10	0.02	1.65E-07	7.57E-10	[IQGAP3,**MEF2D**]
rs4141663	3	124551967	chr3:124331571..124648820	T	C	0.44	0.08	0.02	8.18E-06	-0.15	0.02	1.41E-17	5.36E-22	[**ITGB5**,KALRN,MUC13,UMPS]
rs34995334	6	121845679	chr6:121782750..121848841	A	G	0.17	-0.12	0.03	1.44E-06	0.11	0.02	3.27E-06	3.96E-08	Intergenic
rs3798293	6	97033370	chr6:96786215..97067047	A	G	0.22	-0.15	0.02	5.99E-12	-0.13	0.02	2.70E-10	8.21E-15	[**FHL5**,UFL1]
rs78378222	17	7571752	chr17:7417640..7700063	T	G	0.02	-0.34	0.07	2.91E-06	-0.61	0.08	1.71E-14	1.80E-18	[ATP1B2,CD68,DNAH2,EFNB3,EIF4A1,FXR2,MPDU1,POLR2A,RPL29P2,SAT2,SENP3,SENP3-EIF4A1,SHBG,SNORA48,SNORA67,SNORD10,SOX15,TNFSF12,TNFSF12-TNFSF13,TNFSF13,**TP53**,WRAP53]

Abbreviations: CHR: chromosome; BP: physical position; POS: position; A1FREQ: A1 allele frequency; DBP: diastolic blood pressure.

Position is under build 37/hg19.

All these loci were genome-wide significant ($P<5\times10^{-8}$) for cross-trait meta-analysis (using heterogonous version of CPASSOC, SHet) and $P<1\times10^{-5}$ for single trait GWAS.

Supplementary Table 13 Genome-wide significant loci shared between migraine without aura and systolic blood pressure (SBP) from cross-trait meta-analysis using CPASSOC (P_{meta}≤5×10^{-08})

SNP	CHR	BP	POS	A1	A2	A1FREQ1	Migraine without aura			SBP			P_{meta}	Genes within Ranges
							BETA	SE	P	BETA	SE	P		
rs2274319	1	156450873	chr1:156439000..156474119	T	C	0.64	0.09	0.02	2.45E-06	0.16	0.03	2.47E-07	1.14E-08	[**MEF2D**]
rs2983896	6	97029871	chr6:96767685..97081432	A	G	0.22	0.15	0.02	5.82E-12	0.34	0.04	1.61E-20	1.45E-24	[**FHL5**,UFL1]
rs7454157	6	12909874	chr6:12764286..12948388	A	G	0.35	0.11	0.02	8.20E-08	0.21	0.03	1.31E-11	9.53E-14	[**PHACTR1**]

Abbreviations: CHR: chromosome; BP: physical position; POS: position; A1FREQ: A1 allele frequency; SBP: systolic blood pressure.

Position is under build 37/hg19.

All these loci were genome-wide significant (P<5×10^{-8}) for cross-trait meta-analysis (using heterogonous version of CPASSOC, SHet) and P<1×10^{-5} for single trait GWAS.

Supplementary Table 14 Genome-wide significant loci shared between migraine without aura and pulse pressure (PP) from cross-trait meta-analysis using CPASSOC (P_{meta}≤5×10^{-08})

SNP	CHR	BP	POS	A1	A2	A1FREQ1	Migraine without aura			PP			P_{meta}	Genes within Ranges
							BETA	SE	*P*	BETA	SE	*P*		
rs11172113	12	57527283	chr12:57362422..57540751	T	C	0.45	0.16	0.02	4.27E-16	-0.12	0.02	3.02E-08	1.68E-17	[GPR182,**LRP1**,MYO1A,NAB2,STAT6,TAC3,TMEM194A,ZBTB39]
rs12631961	3	124561848	chr3:124450321..124648336	T	C	0.42	0.09	0.02	5.16E-06	-0.13	0.02	6.51E-10	8.23E-13	[**ITGB5**,MUC13,UMPS]
rs1332844	6	12889004	chr6:12762814..13052693	T	C	0.65	-0.10	0.02	1.95E-07	-0.20	0.02	6.95E-22	2.84E-28	[**PHACTR1**]
rs2983896	6	97029871	chr6:96767685..97070190	A	G	0.22	0.15	0.02	5.82E-12	0.21	0.02	1.26E-17	1.52E-24	[**FHL5,**UFL1]
rs78378222	17	7571752	chr17:7417640..7700063	T	G	0.02	-0.34	0.07	2.91E-06	1.05	0.09	1.28E-28	7.32E-36	[ATP1B2,CD68,DNAH2,EFNB3,EIF4A1,FXR2,MPDU1,POLR2A,RPL29P2,SAT2,SENP3,SENP3-EIF4A1,SHBG,SNORA48,SNORA67,SNORD10,SOX15,TNFSF12,TNFSF12-TNFSF13,TNFSF13,**TP53**,WRAP53]
rs80080721	17	47777540	chr17:47661137..47964202	T	G	0.11	0.13	0.03	8.98E-06	-0.18	0.03	9.88E-09	2.70E-11	[FAM117A,FLJ45513,KAT7,NXPH3,**SLC35B1**,SPOP,TAC4]

Abbreviations: CHR: chromosome; BP: physical position; POS: position; A1FREQ: A1 allele frequency; PP: pulse pressure.

Position is under build 37/hg19.

All these loci were genome-wide significant (P<5×10^{-8}) for cross-trait meta-analysis (using heterogonous version of CPASSOC, SHet) and P<1×10^{-5} for single trait GWAS.

Supplementary Table 15 The conditional/joint significant shared TWAS genes between migraine and diastolic blood pressure (DBP)

ID	CHR	Migraine				DBP			
		Tissue	BEST.GWAS.ID	TWAS.Z	$P_{Bonferroni}$	Tissue	BEST.GWAS.ID	TWAS.Z	$P_{Bonferroni}$
KIAA0040	1	Artery_Tibial	rs2272784	5.00	4.75E-03	Skin_Sun_Exposed_Lower_leg	rs2272784	6.08	1.02E-05
						Skin_Not_Sun_Exposed_Suprapubic	rs2272784	5.95	2.00E-05
TSPAN2	1	Artery_Aorta	rs2078371	9.72	1.54E-18	Minor_Salivary_Gland	rs11466111	-4.19	0.05
		Artery_Tibial	rs2078371	7.20	5.01E-09				
ADRA2B	2	Skin_Sun_Exposed_Lower_leg	rs11695514	-5.07	3.45E-03	Esophagus_Gastroesophageal_Junction	rs1813381	11.21	1.82E-25
						Skin_Not_Sun_Exposed_Suprapubic	rs1813381	11.06	1.42E-24
						Adipose_Visceral_Omentum	rs1813381	10.95	4.18E-24
						Thyroid	rs1813381	10.80	3.30E-23
						Adipose_Subcutaneous	rs1813381	10.68	9.83E-23
						Stomach	rs1813381	10.52	2.92E-22
						Colon_Transverse	rs1813381	10.52	3.67E-22
						Skin_Sun_Exposed_Lower_leg	rs1813381	10.31	5.77E-21
						Spleen	rs1813381	10.24	5.73E-21
						Nerve_Tibial	rs1813381	10.23	1.36E-20
						Breast_Mammary_Tissue	rs1813381	10.20	1.01E-20
						Esophagus_Muscularis	rs1813381	10.18	1.88E-20
						Heart_Left_Ventricle	rs1813381	10.11	2.41E-20
						Muscle_Skeletal	rs1813381	9.86	4.52E-19
						Heart_Atrial_Appendage	rs1813381	9.78	7.52E-19
						Testis	rs1813381	-6.44	1.06E-06
						Lung	rs1813381	6.31	2.11E-06
						Brain_Cerebellum	rs1813381	-5.81	3.69E-05
						Artery_Aorta	rs1813381	-5.02	3.34E-03
ITGB5	3	Artery_Coronary	rs4141663	4.58	0.01	Artery_Coronary	rs4141663	-9.09	3.15E-16
		Artery_Tibial	rs4141663	4.77	0.01	Artery_Tibial	rs4141663	-8.46	2.14E-13
		Cells_Transformed_fibroblasts	rs4141663	-5.27	9.99E-04	Colon_Sigmoid	rs4141663	-8.35	3.31E-13
		Esophagus_Mucosa	rs4141663	-5.35	6.86E-04	Esophagus_Muscularis	rs4141663	-8.27	9.94E-13

		Nerve_Tibial	rs4141663	5.08	3.54E-03	Nerve_Tibial	rs4141663	-8.27	1.26E-12
						Esophagus_Gastroesophageal_Junction	rs4141663	-8.20	1.15E-12
						Artery_Aorta	rs4141663	-8.09	3.77E-12
						Esophagus_Mucosa	rs4141663	7.77	6.21E-11
						Brain_Hypothalamus	rs4141663	-6.57	1.14E-07
						Muscle_Skeletal	rs4141663	-4.52	0.05
RP11-71H17.9	3	Brain_Cerebellum	rs4141663	-4.81	0.01	Brain_Cerebellum	rs4141663	5.14	1.60E-03
		Brain_Nucleus_accumbens_basal_ganglia	rs4141663	-4.50	0.02	Ovary	rs4141663	4.39	0.03
CISD2	4	Artery_Tibial	rs7694724	4.61	0.03	Ovary	rs13116385	8.31	2.53E-13
						Colon_Sigmoid	rs13116385	7.87	1.64E-11
						Esophagus_Mucosa	rs13116385	7.87	2.86E-11
						Nerve_Tibial	rs13116385	7.63	2.28E-10
						Skin_Sun_Exposed_Lower_leg	rs13116385	7.39	1.24E-09
						Breast_Mammary_Tissue	rs13116385	7.28	1.64E-09
						Cells_Transformed_fibroblasts	rs13116385	7.16	5.83E-09
						Esophagus_Muscularis	rs13116385	7.02	1.68E-08
						Thyroid	rs13116385	6.90	4.99E-08
						Brain_Hippocampus	rs13116385	6.70	4.53E-08
						Uterus	rs13116385	6.56	1.11E-07
						Brain_Cerebellar_Hemisphere	rs13116385	6.44	5.24E-07
						Adipose_Visceral_Omentum	rs13116385	6.33	1.53E-06
						Artery_Tibial	rs13116385	5.84	4.11E-05
						Adipose_Subcutaneous	rs13116385	5.76	6.59E-05
						Lung	rs13116385	5.48	3.25E-04
						Skin_Not_Sun_Exposed_Suprapubic	rs13116385	4.83	0.01
ANKDD1B	5	Esophagus_Mucosa	rs34358	-4.85	0.01	Esophagus_Mucosa	rs2307111	-9.47	2.19E-17
		Nerve_Tibial	rs34358	-4.65	0.03	Thyroid	rs2307111	-8.99	2.41E-15
					0.03	Nerve_Tibial	rs2307111	-8.72	2.61E-14
					0.03	Brain_Cerebellum	rs2307111	-8.06	4.50E-12
					0.03	Skin_Not_Sun_Exposed_Suprapubic	rs2307111	-8.01	8.52E-12

					0.03	Colon_Sigmoid	rs2307111	-7.97	7.66E-12
					0.03	Esophagus_Muscularis	rs2307111	-7.39	1.12E-09
					0.03	Skin_Sun_Exposed_Lower_leg	rs2307111	-6.64	2.81E-07
					0.03	Cells_Transformed_fibroblasts	rs2307111	-6.28	2.51E-06
					0.03	Brain_Cerebellar_Hemisphere	rs2307111	-6.16	3.17E-06
GJA1	6	Artery_Tibial	rs9320821	5.33	7.94E-04	Artery_Aorta	rs6932373	-8.78	1.08E-14
		Adipose_Subcutaneous	rs2983896	9.95	2.01E-19	Spleen	rs11756315	6.17	2.97E-06
		Artery_Tibial	rs2983896	10.14	2.99E-20	Nerve_Tibial	rs11756315	6.09	1.09E-05
		Brain_Cerebellum	rs2983896	9.49	1.32E-17	Pancreas	rs11756315	5.74	4.64E-05
		Nerve_Tibial	rs2983896	9.81	1.02E-18	Whole_Blood	rs11756315	5.68	8.01E-05
UFL1	6	Pancreas	rs2983896	10.44	8.56E-22	Stomach	rs11756315	5.55	1.24E-04
		Spleen	rs2983896	9.18	1.87E-16	Artery_Tibial	rs11756315	5.52	2.69E-04
		Stomach	rs2983896	8.98	1.20E-15	Adipose_Subcutaneous	rs11756315	5.47	3.59E-04
		Vagina	rs2983896	7.52	1.06E-10				
		Whole_Blood	rs2983896	7.16	4.85E-09				
		Vagina	rs495828	4.42	0.02	Artery_Aorta	rs579459	-8.28	7.86E-13
						Adrenal_Gland	rs579459	-7.83	2.21E-11
						Whole_Blood	rs579459	7.82	3.07E-11
						Artery_Tibial	rs579459	-7.26	3.15E-09
						Artery_Coronary	rs579459	-7.23	1.48E-09
						Breast_Mammary_Tissue	rs579459	-6.95	1.84E-08
						Colon_Sigmoid	rs579459	-6.72	9.00E-08
ABO	9					Pituitary	rs579459	-6.56	2.25E-07
						Heart_Atrial_Appendage	rs579459	-6.48	5.13E-07
						Vagina	rs579459	-6.46	2.11E-07
						Adipose_Subcutaneous	rs579459	-6.13	6.89E-06
						Adipose_Visceral_Omentum	rs579459	-6.05	8.67E-06
						Muscle_Skeletal	rs579459	-5.67	1.06E-04
						Colon_Transverse	rs579459	-5.64	8.65E-05
						Esophagus_Mucosa	rs579459	-5.59	1.80E-04

						Brain_Cerebellar_Hemisphere	rs579459	-5.36	3.52E-04
						Heart_Left_Ventricle	rs579459	-5.34	4.55E-04
						Pancreas	rs579459	-4.97	3.35E-03
						Uterus	rs579459	-4.53	0.01
CYP2C9	10	Skin_Not_Sun_Exposed_Suprapubic	rs7078478	-5.73	7.42E-05	Skin_Sun_Exposed_Lower_leg	rs11187960	-8.69	3.11E-14
		Skin_Sun_Exposed_Lower_leg	rs7078478	-4.56	0.04	Skin_Not_Sun_Exposed_Suprapubic	rs11187960	-8.25	1.16E-12
MRVI1	11	Artery_Tibial	rs4909945	6.95	2.99E-08	Artery_Tibial	rs10840378	-6.59	3.50E-07
						Esophagus_Muscularis	rs10840378	6.54	4.75E-07
						Brain_Substantia_nigra	rs10840378	6.51	1.19E-07
						Brain_Cerebellum	rs10840378	6.04	8.92E-06
						Brain_Cortex	rs10840378	5.76	3.32E-05
C12orf5	12	Artery_Tibial	rs2160875	5.44	4.28E-04	Nerve_Tibial	rs3217926	5.05	4.28E-03
		Stomach	rs2160875	4.45	0.04				
CFDP1	16	Adipose_Subcutaneous	rs4888378	5.24	1.29E-03	Brain_Hippocampus	rs766521	-7.20	1.37E-09
		Adipose_Visceral_Omentum	rs4888378	4.52	0.04	Cells_Transformed_fibroblasts	rs766521	-6.84	5.70E-08
		Adrenal_Gland	rs4888378	4.40	0.05	Nerve_Tibial	rs766521	-6.15	7.32E-06
		Artery_Coronary	rs4888378	4.64	0.01	Brain_Cerebellum	rs766521	-5.56	1.58E-04
		Brain_Cortex	rs4888378	4.86	4.61E-03	Muscle_Skeletal	rs766521	-4.95	0.01
		Brain_Hippocampus	rs4888378	6.02	3.97E-06	Breast_Mammary_Tissue	rs766521	-4.78	0.01
		Breast_Mammary_Tissue	rs4888378	5.49	2.01E-04	Artery_Coronary	rs766521	-4.75	0.01
		Cells_EBV-transformed_lymphocytes	rs4888378	5.00	1.57E-03	Adipose_Subcutaneous	rs766521	-4.73	0.02
		Cells_Transformed_fibroblasts	rs4888378	6.34	1.69E-06	Brain_Cortex	rs766521	-4.64	0.01
		Colon_Sigmoid	rs4888378	4.84	0.01	Esophagus_Gastroesophageal_Junction	rs766521	-4.64	0.02
		Esophagus_Gastroesophageal_Junction	rs4888378	4.82	0.01	Pancreas	rs766521	-4.45	0.04
		Esophagus_Muscularis	rs4888378	4.73	0.02	Colon_Sigmoid	rs766521	-4.44	0.04
		Lung	rs4888378	4.60	0.03	Cells_EBV-transformed_lymphocytes	rs766521	-4.43	0.03
		Muscle_Skeletal	rs4888378	5.44	3.82E-04				
		Nerve_Tibial	rs4888378	5.97	2.32E-05				
		Pancreas	rs4888378	5.39	3.55E-04				
		Skin_Not_Sun_Exposed_Suprapubic	rs4888378	5.17	1.67E-03				

		Stomach	rs4888378	4.55	0.02				
		Artery_Aorta	rs4814863	-7.38	9.77E-10	Artery_Aorta	rs4814858	5.32	6.53E-04
SLC24A3	20	Artery_Coronary	rs4814863	-6.75	4.76E-08	Artery_Coronary	rs4814858	5.19	6.72E-04
		Artery_Tibial	rs4814863	-8.43	2.77E-13				

Abbreviations: CHR: chromosome; DBP: diastolic blood pressure.

All these loci were TWAS significant ($P_{\text{Bonferroni}}$<0.05) for both migraine and blood pressure in at least one GTEx tissues, and significant from TWAS conditional/joint analysis for both traits.

Supplementary Table 16 The conditional/joint significant shared TWAS genes between migraine and systolic blood pressure (SBP)

ID	CHR	Migraine				SBP			
		Tissue	BEST.GWAS.ID	TWAS.Z	$P_{Bonferroni}$	Tissue	BEST.GWAS.ID	TWAS.Z	$P_{Bonferroni}$
ITGB5	3	Artery_Coronary	rs4141663	4.58	0.01	Artery_Coronary	rs4141663	-9.27	5.71E-17
		Artery_Tibial	rs4141663	4.77	0.01	Artery_Tibial	rs4141663	-8.88	5.18E-15
		Cells_Transformed_fibroblasts	rs4141663	-5.27	9.99E-04	Esophagus_Muscularis	rs4141663	-8.64	4.31E-14
		Esophagus_Mucosa	rs4141663	-5.35	6.86E-04	Nerve_Tibial	rs4141663	-8.65	4.85E-14
		Nerve_Tibial	rs4141663	5.08	3.54E-03	Artery_Aorta	rs4141663	-8.56	7.23E-14
						Esophagus_Gastroesophageal_Junction	rs4141663	-8.52	7.42E-14
						Colon_Sigmoid	rs4141663	-8.25	7.56E-13
						Esophagus_Mucosa	rs4141663	7.77	6.42E-11
						Brain_Hypothalamus	rs4141663	-7.13	2.19E-09
SNX24	5	Artery_Aorta	rs13172078	-4.91	0.01	Adrenal_Gland	rs1644318	-6.69	9.60E-08
						Skin_Sun_Exposed_Lower_leg	rs1644318	-5.54	2.58E-04
						Artery_Aorta	rs1644318	-5.12	1.91E-03
RP1-257A7.5	6	Artery_Aorta	rs9349379	9.61	4.61E-18	Artery_Tibial	rs9349379	8.60	6.28E-14
		Artery_Coronary	rs9349379	7.62	7.96E-11	Artery_Aorta	rs9349379	8.54	8.50E-14
		Artery_Tibial	rs9349379	9.72	2.10E-18	Artery_Coronary	rs9349379	7.12	3.36E-09
UFL1	6	Adipose_Subcutaneous	rs2983896	9.95	2.01E-19	Spleen	rs2983896	9.17	1.99E-16
		Artery_Tibial	rs2983896	10.14	2.99E-20	Nerve_Tibial	rs2983896	9.15	5.60E-16
		Brain_Cerebellum	rs2983896	9.49	1.32E-17	Pancreas	rs2983896	9.00	1.16E-15
		Nerve_Tibial	rs2983896	9.81	1.02E-18	Whole_Blood	rs2983896	8.51	1.05E-13
		Pancreas	rs2983896	10.44	8.56E-22	Stomach	rs2983896	8.10	2.43E-12
		Spleen	rs2983896	9.18	1.87E-16	Artery_Tibial	rs2983896	7.69	1.16E-10
		Stomach	rs2983896	8.98	1.20E-15	Adipose_Subcutaneous	rs2983896	7.57	2.98E-10
		Vagina	rs2983896	7.52	1.06E-10	Brain_Cerebellum	rs2983896	6.46	5.92E-07
		Whole_Blood	rs2983896	7.16	4.85E-09	Vagina	rs2983896	4.68	0.01
C7orf10	7	Artery_Aorta	rs12533531	-7.76	5.35E-11	Artery_Aorta	rs17171710	8.21	1.37E-12
RBM20	10	Brain_Cortex	rs10885035	4.46	0.03	Artery_Tibial	rs2419577	-4.92	0.01
						Brain_Substantia_nigra	rs2419577	-4.39	0.02

MRVI1	11	Artery_Tibial	rs4909945	6.95	2.99E-08	Esophagus_Muscularis	rs1470260	7.17	5.68E-09
						Brain_Substantia_nigra	rs1470260	5.39	1.12E-04
						Brain_Cerebellum	rs1470260	5.27	7.65E-04
						Brain_Cortex	rs1470260	5.12	1.20E-03
SMG6	17	Esophagus_Mucosa	rs2236375	-4.63	0.03	Esophagus_Mucosa	rs8069947	-7.84	3.60E-11
						Brain_Cerebellum	rs8069947	-6.64	1.85E-07
						Brain_Cortex	rs8069947	-6.12	3.71E-06
						Testis	rs8069947	-5.13	2.57E-03
						Skin_Sun_Exposed_Lower_leg	rs8069947	-4.87	0.01
DMPK	19	Pancreas	rs1548029	4.45	0.04	Pancreas	rs7256192	7.19	3.17E-09
						Artery_Tibial	rs7256192	6.56	4.49E-07
						Artery_Aorta	rs7256192	6.20	3.49E-06
						Adipose_Subcutaneous	rs7256192	6.24	3.62E-06
						Thyroid	rs7256192	5.61	1.97E-04
						Esophagus_Mucosa	rs7256192	5.57	2.06E-04
						Prostate	rs7256192	5.35	2.37E-04
						Nerve_Tibial	rs7256192	5.02	4.84E-03
						Skin_Sun_Exposed_Lower_leg	rs7256192	5.00	0.01
						Breast_Mammary_Tissue	rs7256192	4.69	0.01
						Whole_Blood	rs7256192	4.67	0.02
						Lung	rs7256192	4.71	0.02
						Skin_Not_Sun_Exposed_Suprapubic	rs7256192	4.56	0.04
						Adipose_Visceral_Omentum	rs7256192	4.49	0.04
SLC24A3	20	Artery_Aorta	rs4814863	-7.38	9.77E-10	Artery_Tibial	rs17812022	-6.31	2.26E-06
		Artery_Coronary	rs4814863	-6.75	4.76E-08	Artery_Aorta	rs17812022	-4.57	0.03
		Artery_Tibial	rs4814863	-8.43	2.77E-13				
ASCC2	22	Cells_EBV-transformed_lymphocytes	rs5752933	-4.81	4.11E-03	Brain_Nucleus_accumbens_basal_ganglia	rs12627866	6.18	2.01E-06
						Artery_Tibial	rs12627866	6.01	1.46E-05
						Esophagus_Muscularis	rs12627866	5.95	2.06E-05
						Artery_Aorta	rs12627866	5.92	2.08E-05

Muscle_Skeletal	rs12627866	5.56	1.95E-04
Nerve_Tibial	rs12627866	5.50	3.66E-04
Brain_Cortex	rs12627866	5.08	1.52E-03
Heart_Atrial_Appendage	rs12627866	5.10	1.93E-03
Lung	rs12627866	4.91	0.01
Brain_Cerebellar_Hemisphere	rs12627866	4.47	0.03

Abbreviations: CHR: chromosome; SBP: systolic blood pressure.

All these loci were TWAS significant ($P_{\text{Bonferroni}}$<0.05) for both migraine and blood pressure in at least one GTEx tissues, and significant from TWAS conditional/joint analysis for both traits.

Supplementary Table 17 **The conditional/joint significant shared TWAS genes between migraine and pulse pressure (PP)**

ID	CHR	Migraine				PP			
		Tissue	BEST.GWAS.ID	TWAS.Z	$P_{Bonferroni}$	Tissue	BEST.GWAS.ID	TWAS.Z	$P_{Bonferroni}$
MEF2D	1	Artery_Tibial	rs1925950	7.56	3.25E-10	Artery_Tibial	rs1185700	5.03	3.91E-03
ITGB5	3	Artery_Coronary	rs4141663	4.58	0.01	Artery_Coronary	rs4141663	-6.17	2.16E-06
		Artery_Tibial	rs4141663	4.77	0.01	Artery_Tibial	rs4141663	-6.20	4.62E-06
		Cells_Transformed_fibroblasts	rs4141663	-5.27	9.99E-04	Artery_Aorta	rs4141663	-5.97	1.50E-05
		Esophagus_Mucosa	rs4141663	-5.35	6.86E-04	Esophagus_Muscularis	rs4141663	-5.93	2.30E-05
		Nerve_Tibial	rs4141663	5.08	3.54E-03	Esophagus_Gastroesophageal_Junction	rs4141663	-5.78	3.61E-05
						Nerve_Tibial	rs4141663	-5.82	5.61E-05
						Colon_Sigmoid	rs4141663	-5.38	3.53E-04
						Brain_Hypothalamus	rs4141663	-5.05	1.00E-03
						Esophagus_Mucosa	rs4141663	4.92	0.01
SNX24	5	Artery_Aorta	rs13172078	-4.91	0.01	Adrenal_Gland	rs1644318	-9.30	6.33E-17
						Muscle_Skeletal	rs1644318	-6.76	1.04E-07
						Skin_Sun_Exposed_Lower_leg	rs1644318	-5.67	1.24E-04
						Artery_Aorta	rs1644318	-5.29	7.80E-04
						Brain_Frontal_Cortex_BA9	rs1644318	-4.69	0.01
GJA1	6	Artery_Tibial	rs9320821	5.33	7.94E-04	Adipose_Visceral_Omentum	rs9401457	-6.28	2.06E-06
						Nerve_Tibial	rs9401457	-6.00	1.88E-05
						Artery_Aorta	rs9401457	5.38	4.69E-04
RP1-257A7.5	6	Artery_Aorta	rs9349379	9.61	4.61E-18	Artery_Aorta	rs9349379	12.64	7.74E-33
		Artery_Coronary	rs9349379	7.62	7.96E-11	Artery_Tibial	rs9349379	12.65	9.06E-33
		Artery_Tibial	rs9349379	9.72	2.10E-18	Artery_Coronary	rs9349379	10.83	8.12E-24
UFL1	6	Adipose_Subcutaneous	rs2983896	9.95	2.01E-19	Pancreas	rs2983896	8.42	1.82E-13
		Artery_Tibial	rs2983896	10.14	2.99E-20	Spleen	rs2983896	8.28	5.29E-13
		Brain_Cerebellum	rs2983896	9.49	1.32E-17	Nerve_Tibial	rs2983896	8.28	1.15E-12
		Nerve_Tibial	rs2983896	9.81	1.02E-18	Whole_Blood	rs2983896	7.84	2.71E-11
		Pancreas	rs2983896	10.44	8.56E-22	Stomach	rs2983896	6.99	1.20E-08
		Spleen	rs2983896	9.18	1.87E-16	Artery_Tibial	rs2983896	6.69	1.87E-07
		Stomach	rs2983896	8.98	1.20E-15	Adipose_Subcutaneous	rs2983896	6.56	4.30E-07
		Vagina	rs2983896	7.52	1.06E-10	Brain_Cerebellum	rs2983896	6.03	9.43E-06
		Whole_Blood	rs2983896	7.16	4.85E-09	Vagina	rs2983896	4.40	0.02

C7orf10	7	Artery_Aorta	rs12533531	-7.76	5.35E-11	Artery_Aorta	rs17171710	10.78	2.77E-23
RBM20	10	Brain_Cortex	rs10885035	4.46	0.03	Brain_Substantia_nigra	rs4918582	-5.52	5.33E-05
						Artery_Aorta	rs4918582	-4.56	0.03
SMG6	17	Esophagus_Mucosa	rs2236375	-4.63	0.03	Esophagus_Mucosa	rs1048483	-8.25	1.23E-12
						Brain_Cortex	rs1048483	-6.81	3.86E-08
						Brain_Cerebellum	rs1048483	-5.11	1.89E-03
						Cells_Transformed_fibroblasts	rs1048483	-5.12	2.26E-03
						Adipose_Subcutaneous	rs1048483	-4.57	0.04
DMPK	19	Pancreas	rs1548029	4.45	0.04	Pancreas	rs11672660	7.58	1.70E-10
						Artery_Tibial	rs11672660	7.30	2.39E-09
						Artery_Aorta	rs11672660	7.15	5.68E-09
						Adipose_Subcutaneous	rs11672660	6.99	2.22E-08
						Esophagus_Mucosa	rs11672660	6.46	8.33E-07
						Thyroid	rs11672660	6.28	3.22E-06
						Prostate	rs11672660	5.96	7.02E-06
						Skin_Sun_Exposed_Lower_leg	rs11672660	5.59	1.96E-04
						Nerve_Tibial	rs11672660	5.58	2.33E-04
						Breast_Mammary_Tissue	rs11672660	5.26	7.08E-04
						Lung	rs11672660	5.30	8.72E-04
						Whole_Blood	rs11672660	5.18	1.31E-03
						Skin_Not_Sun_Exposed_Suprapubic	rs11672660	5.13	2.12E-03
						Adipose_Visceral_Omentum	rs11672660	5.06	2.54E-03
						Spleen	rs11672660	4.69	0.01
SLC24A3	20	Artery_Aorta	rs4814863	-7.38	9.77E-10	Artery_Tibial	rs3790228	-12.25	1.32E-30
		Artery_Coronary	rs4814863	-6.75	4.76E-08	Artery_Aorta	rs3790228	-10.88	8.94E-24
		Artery_Tibial	rs4814863	-8.43	2.77E-13	Artery_Coronary	rs3790228	-7.29	9.64E-10
						Cells_Transformed_fibroblasts	rs3790228	-7.37	1.25E-09
						Adipose_Subcutaneous	rs3790228	-6.01	1.47E-05
ASCC2	22	Cells_EBV-transformed_lymphocytes	rs5752933	-4.81	4.11E-03	Brain_Nucleus_accumbens_basal_ganglia	rs16988381	6.38	5.55E-07
						Esophagus_Muscularis	rs16988381	6.44	9.18E-07
						Artery_Aorta	rs16988381	6.20	3.51E-06
						Artery_Tibial	rs16988381	6.21	4.41E-06
						Nerve_Tibial	rs16988381	5.68	1.27E-04
						Brain_Cortex	rs16988381	5.29	4.89E-04

				Brain_Cerebellar_Hemisphere	rs16988381	4.67	0.01
				Lung	rs16988381	4.75	0.02
				Brain_Cerebellum	rs16988381	4.63	0.02
				Muscle_Skeletal	rs16988381	4.65	0.02

Abbreviations: CHR: chromosome; PP: pulse pressure.

All these loci were TWAS significant ($P_{\text{Bonferroni}}$<0.05) for both migraine and blood pressure in at least one GTEx tissues, and significant from TWAS conditional/joint analysis for both traits.

Supplementary Table 18 The shared TWAS genes between migraine with aura and diastolic blood pressure (DBP)

ID	CHR	Migraine with aura				DBP			
		Tissue	BEST.GWAS.ID	TWAS.Z	$P_{Bonferroni}$	Tissue	BEST.GWAS.ID	TWAS.Z	$P_{Bonferroni}$
MST1	3	Brain_Anterior_cingulate_cortex_BA24	rs4855881	4.31	0.04	Spleen	rs11130216	-7.17	3.27E-09
						Skin_Sun_Exposed_Lower_leg	rs11130216	-7.08	1.23E-08
						Heart_Left_Ventricle	rs11130216	-6.48	4.44E-07
						Stomach	rs11130216	-6.39	7.30E-07
						Lung	rs11130216	-6.33	1.89E-06
						Cells_Transformed_fibroblasts	rs11130216	-6.21	3.83E-06
						Thyroid	rs11130216	-6.17	6.70E-06
						Esophagus_Mucosa	rs11130216	-5.66	1.20E-04
						Brain_Cerebellum	rs11130216	-5.65	9.09E-05
						Brain_Hypothalamus	rs11130216	-5.45	1.15E-04
						Adipose_Subcutaneous	rs11130216	-5.43	4.51E-04
						Artery_Coronary	rs11130216	-4.77	0.01
						Brain_Anterior_cingulate_cortex_BA24	rs11130216	-4.55	0.01
						Brain_Hippocampus	rs11130216	-4.28	0.04
RP11-624M8.1	6	Brain_Amygdala	rs1832937	-4.22	0.04	Lung	rs6919947	-5.19	1.58E-03
UFL1	6	Brain_Cerebellum	rs13208321	4.46	0.05	Spleen	rs11756315	6.17	2.97E-06
						Nerve_Tibial	rs11756315	6.09	1.09E-05
						Pancreas	rs11756315	5.74	4.64E-05
						Whole_Blood	rs11756315	5.68	8.01E-05
						Stomach	rs11756315	5.55	1.24E-04
						Artery_Tibial	rs11756315	5.52	2.69E-04
						Adipose_Subcutaneous	rs11756315	5.47	3.59E-04
RHOF	12	Minor_Salivary_Gland	rs34395322	4.49	0.01	Minor_Salivary_Gland	rs11059094	4.55	0.01
HMOX2	16	Artery_Tibial	rs2304634	4.88	0.01	Spleen	rs12446456	6.91	2.13E-08
PDE6G	17	Muscle_Skeletal	rs1122424	5.10	2.43E-03	Testis	rs11150782	-4.73	0.02

Brain_Cerebellum	rs11150782	-4.60	0.02

Abbreviations: CHR: chromosome; DBP: diastolic blood pressure.

All these loci were TWAS significant ($P_{Bonferroni}$<0.05) for both migraine and blood pressure in at least one GTEx tissues.

Supplementary Table 19 **The shared TWAS genes between migraine with aura and systolic blood pressure (SBP)**

ID	CHR	Migraine with aura				SBP			
		Tissue	BEST.GWAS.ID	TWAS.Z	$P_{Bonferroni}$	Tissue	BEST.GWAS.ID	TWAS.Z	$P_{Bonferroni}$
MST1	3	Brain_Anterior_cingulate_cortex_BA24	rs4855881	4.31	0.04	Spleen	rs13100791	-5.74	4.22E-05
						Skin_Sun_Exposed_Lower_leg	rs13100791	-5.57	2.28E-04
						Brain_Hypothalamus	rs13100791	-4.95	1.64E-03
						Brain_Cerebellum	rs13100791	-5.09	2.06E-03
						Thyroid	rs13100791	-5.04	4.45E-03
						Heart_Left_Ventricle	rs13100791	-4.87	0.01
						Stomach	rs13100791	-4.70	0.01
						Esophagus_Mucosa	rs13100791	-4.70	0.02
						Lung	rs13100791	-4.59	0.03
RP11-624M8.1	6	Brain_Amygdala	rs1832937	-4.22	0.04	Lung	rs6900852	-6.92	3.40E-08
						Brain_Hippocampus	rs6900852	-5.36	1.85E-04
						Brain_Spinal_cord_cervical_c-1	rs6900852	-5.06	8.17E-04
						Ovary	rs6900852	-5.00	1.56E-03
						Brain_Cerebellar_Hemisphere	rs6900852	-4.96	3.01E-03
						Brain_Anterior_cingulate_cortex_BA24	rs6900852	-4.82	3.85E-03
						Brain_Hypothalamus	rs6900852	-4.62	0.01
						Pancreas	rs6900852	-4.75	0.01
						Brain_Amygdala	rs6900852	-4.52	0.01
						Nerve_Tibial	rs6900852	-4.85	0.01
						Thyroid	rs6900852	-4.79	0.02
						Esophagus_Gastroesophageal_Junction	rs6900852	-4.65	0.02
UFL1	6	Brain_Cerebellum	rs13208321	4.46	0.05	Spleen	rs2983896	9.17	1.99E-16
						Nerve_Tibial	rs2983896	9.15	5.60E-16
						Pancreas	rs2983896	9.00	1.16E-15
						Whole_Blood	rs2983896	8.51	1.05E-13
						Stomach	rs2983896	8.10	2.43E-12

						Artery_Tibial	rs2983896	7.69	1.16E-10
						Adipose_Subcutaneous	rs2983896	7.57	2.98E-10
						Brain_Cerebellum	rs2983896	6.46	5.92E-07
						Vagina	rs2983896	4.68	0.01
RHOF	12	Minor_Salivary_Gland	rs34395322	4.49	0.01	Minor_Salivary_Gland	rs4069666	4.51	0.01
NMRAL1	16	Adrenal_Gland	rs2304634	4.42	0.04	Brain_Anterior_cingulate_cortex_BA24	rs12446456	4.41	0.03
HMOX2	16	Artery_Tibial	rs2304634	4.88	0.01	Spleen	rs12446456	9.22	1.26E-16
						Adipose_Visceral_Omentum	rs12446456	6.16	4.33E-06
						Brain_Cerebellum	rs12446456	4.96	4.16E-03
						Thyroid	rs12446456	4.63	0.04
NMRAL1	16	Muscle_Skeletal	rs2304634	4.58	0.03	Brain_Anterior_cingulate_cortex_BA24	rs12446456	4.41	0.03
		Prostate	rs2304634	4.42	0.03				
		Thyroid	rs2304634	4.77	0.02				
PAM16	16	Whole_Blood	rs2304634	-4.47	0.05	Spleen	rs9935080	-4.73	0.01
						Stomach	rs9935080	-4.61	0.02
						Heart_Left_Ventricle	rs9935080	4.48	0.04

Abbreviations: CHR: chromosome; SBP: systolic blood pressure.

All these loci were TWAS significant ($P_{\text{Bonferroni}}$<0.05) for both migraine and blood pressure in at least one GTEx tissues.

Supplementary Table 20 The shared TWAS genes between migraine with aura and pulse pressure (PP)

ID	CHR	Migraine with aura				PP			
		Tissue	BEST.GWAS.ID	TWAS.Z	$P_{Bonferroni}$	Tissue	BEST.GWAS.ID	TWAS.Z	$P_{Bonferroni}$
RP11-624M8.1	6	Brain_Amygdala	rs1832937	-4.22	0.04	Lung	rs4897160	-5.43	4.28E-04
						Brain_Hippocampus	rs4897160	-4.87	2.49E-03
UFL1	6	Brain_Cerebellum	rs13208321	4.46	0.05	Pancreas	rs2983896	8.42	1.82E-13
						Spleen	rs2983896	8.28	5.29E-13
						Nerve_Tibial	rs2983896	8.28	1.15E-12
						Whole_Blood	rs2983896	7.84	2.71E-11
						Stomach	rs2983896	6.99	1.20E-08
						Artery_Tibial	rs2983896	6.69	1.87E-07
						Adipose_Subcutaneous	rs2983896	6.56	4.30E-07
						Brain_Cerebellum	rs2983896	6.03	9.43E-06
						Vagina	rs2983896	4.40	0.02
LRP1	12	Artery_Tibial	rs11172113	4.87	0.01	Skin_Sun_Exposed_Lower_leg	rs11172113	-5.75	7.71E-05
						Artery_Tibial	rs11172113	-5.54	2.44E-04
NMRAL1	16	Adrenal_Gland	rs2304634	4.42	0.04	Brain_Anterior_cingulate_cortex_BA24	rs4785955	5.52	8.94E-05
		Muscle_Skeletal	rs2304634	4.58	0.03	Skin_Sun_Exposed_Lower_leg	rs4785955	5.69	1.08E-04
		Prostate	rs2304634	4.42	0.03	Esophagus_Gastroesophageal_Junction	rs4785955	5.56	1.31E-04
		Thyroid	rs2304634	4.77	0.02	Brain_Putamen_basal_ganglia	rs4785955	5.27	3.84E-04
						Brain_Cortex	rs4785955	5.22	7.02E-04
						Lung	rs4785955	5.27	1.06E-03
						Ovary	rs4785955	5.04	1.27E-03
						Cells_Transformed_fibroblasts	rs4785955	5.21	1.40E-03
						Heart_Left_Ventricle	rs4785955	5.06	2.06E-03
						Stomach	rs4785955	5.04	2.08E-03
						Liver	rs4785955	4.95	2.15E-03
						Pancreas	rs4785955	5.05	2.18E-03
						Esophagus_Muscularis	rs4785955	5.13	2.23E-03
						Breast_Mammary_Tissue	rs4785955	5.03	2.37E-03

						Esophagus_Mucosa	rs4785955	5.11	2.53E-03
						Pituitary	rs4785955	4.97	2.85E-03
						Colon_Sigmoid	rs4785955	4.99	2.94E-03
						Nerve_Tibial	rs4785955	5.12	2.94E-03
						Brain_Cerebellar_Hemisphere	rs4785955	4.95	3.14E-03
						Whole_Blood	rs4785955	5.00	3.41E-03
						Artery_Tibial	rs4785955	5.02	4.08E-03
						Brain_Hippocampus	rs4785955	4.65	0.01
						Brain_Cerebellum	rs4785955	4.74	0.01
						Heart_Atrial_Appendage	rs4785955	4.73	0.01
						Adipose_Visceral_Omentum	rs4785955	4.69	0.02
						Skin_Not_Sun_Exposed_Suprapubic	rs4785955	4.72	0.02
						Brain_Nucleus_accumbens_basal_ganglia	rs4785955	4.50	0.02
HMOX2	16	Artery_Tibial	rs2304634	4.88	0.01	Spleen	rs4785955	7.49	2.94E-10
						Brain_Cerebellum	rs4785955	6.11	5.69E-06
						Adipose_Visceral_Omentum	rs4785955	5.94	1.75E-05
						Thyroid	rs4785955	5.67	1.37E-04
						Brain_Nucleus_accumbens_basal_ganglia	rs4785955	-5.10	1.06E-03
						Breast_Mammary_Tissue	rs4785955	4.90	4.71E-03
						Brain_Caudate_basal_ganglia	rs4785955	-4.79	0.01
						Lung	rs4785955	4.83	0.01
						Nerve_Tibial	rs4785955	4.72	0.02
PAM16	16	Whole_Blood	rs2304634	-4.47	0.05	Stomach	rs4785955	-5.18	9.88E-04

Abbreviations: CHR: chromosome; PP: pulse pressure.

All these loci were TWAS significant ($P_{\text{Bonferroni}}$<0.05) for both migraine and blood pressure in at least one GTEx tissues.

Supplementary Table 21 The shared TWAS genes between migraine without aura and diastolic blood pressure (DBP)

ID	CHR	Migraine without aura				DBP			
		Tissue	BEST.GWAS.ID	TWAS.Z	$P_{\text{Bonferroni}}$	Tissue	BEST.GWAS.ID	TWAS.Z	$P_{\text{Bonferroni}}$
TSPAN2	1	Artery_Aorta	rs2078371	5.66	9.45E-05	Minor_Salivary_Gland	rs11466111	-4.19	0.05
ITGB5	3	Esophagus_Mucosa	rs1265646	-4.87	0.01	Artery_Coronary	rs4141663	-9.09	3.15E-16
		Nerve_Tibial	rs1265646	4.80	0.02	Artery_Tibial	rs4141663	-8.46	2.14E-13
						Colon_Sigmoid	rs4141663	-8.35	3.31E-13
						Esophagus_Muscularis	rs4141663	-8.27	9.94E-13
						Nerve_Tibial	rs4141663	-8.27	1.26E-12
						Esophagus_Gastroesophageal_Junction	rs4141663	-8.20	1.15E-12
						Artery_Aorta	rs4141663	-8.09	3.77E-12
						Esophagus_Mucosa	rs4141663	7.77	6.21E-11
						Brain_Hypothalamus	rs4141663	-6.57	1.14E-07
						Muscle_Skeletal	rs4141663	-4.52	0.05
MANBA	4	Small_Intestine_Terminal_Ileum	rs228614	4.39	0.03	Artery_Aorta	rs13107325	-8.82	6.98E-15
						Breast_Mammary_Tissue	rs13107325	-8.40	2.31E-13
						Cells_Transformed_fibroblasts	rs13107325	-8.39	3.49E-13
						Adrenal_Gland	rs13107325	-7.80	2.67E-11
						Brain_Cerebellar_Hemisphere	rs13107325	7.15	3.78E-09
						Spleen	rs13107325	6.55	2.52E-07
						Brain_Hippocampus	rs13107325	6.18	1.41E-06
						Small_Intestine_Terminal_Ileum	rs13107325	5.81	1.76E-05
						Testis	rs13107325	5.23	1.56E-03
U91328.19	6	Brain_Putamen_basal_ganglia	rs17531978	4.32	0.04	Brain_Cerebellum	rs198846	-10.43	1.08E-21
						Brain_Cerebellar_Hemisphere	rs198846	-8.54	5.84E-14
						Brain_Spinal_cord_cervical_c-1	rs198846	-7.42	2.25E-10
						Brain_Nucleus_accumbens_basal_ganglia	rs198846	-6.71	6.25E-08
						Colon_Sigmoid	rs198846	-5.11	1.57E-03
						Brain_Caudate_basal_ganglia	rs198846	-4.94	2.75E-03
						Brain_Hippocampus	rs198846	-4.75	4.53E-03
						Brain_Putamen_basal_ganglia	rs198846	-4.55	0.01
UFL1	6	Adipose_Subcutaneous	rs2273621	5.89	3.05E-05	Spleen	rs11756315	6.17	2.97E-06

		Artery_Tibial	rs2273621	6.13	7.34E-06	Nerve_Tibial	rs11756315	6.09	1.09E-05
		Brain_Cerebellum	rs2273621	6.62	2.06E-07	Pancreas	rs11756315	5.74	4.64E-05
		Nerve_Tibial	rs2273621	5.81	5.92E-05	Whole_Blood	rs11756315	5.68	8.01E-05
		Pancreas	rs2273621	6.67	1.33E-07	Stomach	rs11756315	5.55	1.24E-04
		Spleen	rs2273621	5.11	1.42E-03	Artery_Tibial	rs11756315	5.52	2.69E-04
		Stomach	rs2273621	5.19	9.44E-04	Adipose_Subcutaneous	rs11756315	5.47	3.59E-04
		Vagina	rs2273621	5.88	7.83E-06				
		Whole_Blood	rs2273621	4.86	0.01				
HVCN1	12	Brain_Hippocampus	rs17619763	5.36	1.87E-04	Brain_Hippocampus	rs11065822	-9.04	3.61E-16
						Whole_Blood	rs11065822	6.39	9.77E-07

Abbreviations: CHR: chromosome; DBP: diastolic blood pressure.

All these loci were TWAS significant ($P_{Bonferroni}$<0.05) for both migraine and blood pressure in at least one GTEx tissues.

Supplementary Table 22 The shared TWAS genes between migraine without aura and systolic blood pressure (SBP)

ID	CHR	Migraine without aura				SBP			
		Tissue	BEST.GWAS.ID	TWAS.Z	$P_{Bonferroni}$	Tissue	BEST.GWAS.ID	TWAS.Z	$P_{Bonferroni}$
ITGB5	3	Esophagus_Mucosa	rs1265646	-4.87	0.01	Artery_Coronary	rs4141663	-9.27	5.71E-17
		Nerve_Tibial	rs1265646	4.80	0.02	Artery_Tibial	rs4141663	-8.88	5.18E-15
						Esophagus_Muscularis	rs4141663	-8.64	4.31E-14
						Nerve_Tibial	rs4141663	-8.65	4.85E-14
						Artery_Aorta	rs4141663	-8.56	7.23E-14
						Esophagus_Gastroesophageal_Junction	rs4141663	-8.52	7.42E-14
						Colon_Sigmoid	rs4141663	-8.25	7.56E-13
						Esophagus_Mucosa	rs4141663	7.77	6.42E-11
						Brain_Hypothalamus	rs4141663	-7.13	2.19E-09
FHL5	6	Artery_Tibial	rs2273621	-5.05	3.55E-03	Artery_Tibial	rs2983896	-6.00	1.64E-05
PHACTR1	6	Artery_Aorta	rs9349379	6.29	2.03E-06	Artery_Aorta	rs9349379	8.50	1.24E-13
		Artery_Tibial	rs9349379	5.77	6.34E-05	Artery_Tibial	rs9349379	7.31	2.23E-09
		Brain_Spinal_cord_cervical_c-1	rs9349379	4.62	0.01				
RP1-257A7.5	6	Artery_Aorta	rs9349379	6.01	1.19E-05	Artery_Tibial	rs9349379	8.60	6.28E-14
		Artery_Coronary	rs9349379	6.20	1.84E-06	Artery_Aorta	rs9349379	8.54	8.50E-14
		Artery_Tibial	rs9349379	6.22	3.90E-06	Artery_Coronary	rs9349379	7.12	3.36E-09
U91328.19	6	Brain_Putamen_basal_ganglia	rs17531978	4.32	0.04	Brain_Cerebellum	rs198846	-6.40	9.15E-07
						Brain_Cerebellar_Hemisphere	rs198846	-5.11	1.40E-03
UFL1	6	Adipose_Subcutaneous	rs2273621	5.89	3.05E-05	Spleen	rs2983896	9.17	1.99E-16
		Artery_Tibial	rs2273621	6.13	7.34E-06	Nerve_Tibial	rs2983896	9.15	5.60E-16
		Brain_Cerebellum	rs2273621	6.62	2.06E-07	Pancreas	rs2983896	9.00	1.16E-15
		Nerve_Tibial	rs2273621	5.81	5.92E-05	Whole_Blood	rs2983896	8.51	1.05E-13
		Pancreas	rs2273621	6.67	1.33E-07	Stomach	rs2983896	8.10	2.43E-12
		Spleen	rs2273621	5.11	1.42E-03	Artery_Tibial	rs2983896	7.69	1.16E-10
		Stomach	rs2273621	5.19	9.44E-04	Adipose_Subcutaneous	rs2983896	7.57	2.98E-10
		Vagina	rs2273621	5.88	7.83E-06	Brain_Cerebellum	rs2983896	6.46	5.92E-07
		Whole_Blood	rs2273621	4.86	0.01	Vagina	rs2983896	4.68	0.01
HVCN1	12	Brain_Hippocampus	rs17619763	5.36	1.87E-04	Brain_Hippocampus	rs11065822	-4.93	1.86E-03
						Whole_Blood	rs11065822	4.94	4.59E-03

Abbreviations: CHR: chromosome; SBP: systolic blood pressure.

All these loci were TWAS significant ($P_{\text{Bonferroni}}$<0.05) for both migraine and blood pressure in at least one GTEx tissues.

Supplementary Table 23 The shared TWAS genes between migraine without aura and pulse pressure (PP)

ID	CHR	Migraine without aura				PP			
		Tissue	BEST.GWAS.ID	TWAS.Z	$P_{Bonferroni}$	Tissue	BEST.GWAS.ID	TWAS.Z	$P_{Bonferroni}$
ITGB5	3	Esophagus_Mucosa	rs1265646	-4.87	0.01	Artery_Coronary	rs4141663	-6.17	2.16E-06
		Nerve_Tibial	rs1265646	4.80	0.02	Artery_Tibial	rs4141663	-6.20	4.62E-06
						Artery_Aorta	rs4141663	-5.97	1.50E-05
						Esophagus_Muscularis	rs4141663	-5.93	2.30E-05
						Esophagus_Gastroesophageal_Junction	rs4141663	-5.78	3.61E-05
						Nerve_Tibial	rs4141663	-5.82	5.61E-05
						Colon_Sigmoid	rs4141663	-5.38	3.53E-04
						Brain_Hypothalamus	rs4141663	-5.05	1.00E-03
						Esophagus_Mucosa	rs4141663	4.92	0.01
MANBA	4	Small_Intestine_Terminal_Ileum	rs228614	4.39	0.03	Brain_Cerebellum	rs13107325	-6.56	3.19E-07
						Artery_Aorta	rs13107325	5.50	2.38E-04
						Breast_Mammary_Tissue	rs13107325	5.26	7.08E-04
						Adrenal_Gland	rs13107325	5.03	2.13E-03
						Spleen	rs13107325	-4.96	3.05E-03
						Brain_Cerebellar_Hemisphere	rs13107325	-4.85	0.01
						Cells_Transformed_fibroblasts	rs13107325	4.89	0.01
FHL5	6	Artery_Tibial	rs2273621	-5.05	3.55E-03	Artery_Tibial	rs2983896	-6.12	7.64E-06
PHACTR1	6	Artery_Aorta	rs9349379	6.29	2.03E-06	Artery_Aorta	rs9349379	12.39	1.83E-31
		Artery_Tibial	rs9349379	5.77	6.34E-05	Artery_Tibial	rs9349379	11.13	6.97E-25
		Brain_Spinal_cord_cervical_c-1	rs9349379	4.62	0.01	Breast_Mammary_Tissue	rs9349379	5.46	2.40E-04
						Cells_Transformed_fibroblasts	rs9349379	5.19	1.53E-03
RP1-257A7.5	6	Artery_Aorta	rs9349379	6.01	1.19E-05	Artery_Aorta	rs9349379	12.64	7.74E-33
		Artery_Coronary	rs9349379	6.20	1.84E-06	Artery_Tibial	rs9349379	12.65	9.06E-33
		Artery_Tibial	rs9349379	6.22	3.90E-06	Artery_Coronary	rs9349379	10.83	8.12E-24
U91328.19	6	Brain_Putamen_basal_ganglia	rs17531978	4.32	0.04	Whole_Blood	rs13161	6.27	2.12E-06
						Uterus	rs13161	4.33	0.03
UFL1	6	Adipose_Subcutaneous	rs2273621	5.89	3.05E-05	Pancreas	rs2983896	8.42	1.82E-13
		Artery_Tibial	rs2273621	6.13	7.34E-06	Spleen	rs2983896	8.28	5.29E-13
		Brain_Cerebellum	rs2273621	6.62	2.06E-07	Nerve_Tibial	rs2983896	8.28	1.15E-12
		Nerve_Tibial	rs2273621	5.81	5.92E-05	Whole_Blood	rs2983896	7.84	2.71E-11

		Pancreas	rs2273621	6.67	1.33E-07	Stomach	rs2983896	6.99	1.20E-08
		Spleen	rs2273621	5.11	1.42E-03	Artery_Tibial	rs2983896	6.69	1.87E-07
		Stomach	rs2273621	5.19	9.44E-04	Adipose_Subcutaneous	rs2983896	6.56	4.30E-07
		Vagina	rs2273621	5.88	7.83E-06	Brain_Cerebellum	rs2983896	6.03	9.43E-06
		Whole_Blood	rs2273621	4.86	0.01	Vagina	rs2983896	4.40	0.02
HBCBP	12	Adrenal_Gland	rs11172113	4.85	0.01	Adrenal_Gland	rs11172113	-7.59	1.44E-10
LRP1	12	Artery_Tibial	rs11172113	8.13	3.45E-12	Skin_Sun_Exposed_Lower_leg	rs11172113	-5.75	7.71E-05
						Artery_Tibial	rs11172113	-5.54	2.44E-04
TMEM194A	12	Heart_Atrial_Appendage	rs11172113	-4.93	4.57E-03	Heart_Atrial_Appendage	rs11172113	5.10	1.87E-03
ATP5SL	19	Brain_Cortex	rs3810174	4.48	0.03	Brain_Caudate_basal_ganglia	rs4803457	-4.71	0.01
C19orf69	19	Lung	rs3810174	4.62	0.03	Lung	rs4803457	-5.15	2.03E-03
						Nerve_Tibial	rs4803457	-5.09	3.48E-03
						Esophagus_Mucosa	rs4803457	-4.81	0.01
						Muscle_Skeletal	rs4803457	-4.53	0.04
						Adipose_Subcutaneous	rs4803457	-4.53	0.05

Abbreviations: CHR: chromosome; PP: pulse pressure.

All these loci were TWAS significant ($P_{\text{Bonferroni}}$<0.05) for both migraine and blood pressure in at least one GTEx tissues.

Supplementary Table 24 **Sensitivity analysis for mendelian randomization using IVW, weighted median, simple median, and MR-egger**

Method	Exposure	Outcome	Beta	SE	P
	β_{DBP}		0.16	0.02	<0.001
	β_{SBP}	$\beta_{Migraine}$	0.05	0.01	<0.001
	β_{PP}		-0.05	0.03	0.04
	β_{DBP}		0.14	0.05	0.01
IVW	β_{SBP}	β_{MA}	0.07	0.03	0.03
	β_{PP}		-0.06	0.05	0.24
	β_{DBP}		0.30	0.05	<0.001
	β_{SBP}	β_{MO}	0.07	0.03	0.03
	β_{PP}		-0.18	0.05	0.001
	β_{DBP}		0.15	0.03	<0.001
	β_{SBP}	$\beta_{Migraine}$	0.03	0.02	0.07
	β_{PP}		-0.09	0.03	<0.001
	β_{DBP}		0.06	0.08	0.45
Weighted Median	β_{SBP}	β_{MA}	0.01	0.05	0.81
	β_{PP}		-0.11	0.08	0.16
	β_{DBP}		0.30	0.07	<0.001
	β_{SBP}	β_{MO}	0.08	0.04	0.06
	β_{PP}		-0.15	0.07	0.04
	β_{DBP}		0.18	0.03	<0.001
	β_{SBP}	$\beta_{Migraine}$	0.03	0.02	0.04
	β_{PP}		-0.07	0.03	0.01
	β_{DBP}		0.10	0.08	0.19
Simple Median	β_{SBP}	β_{MA}	0.02	0.05	0.71
	β_{PP}		-0.05	0.07	0.51
	β_{DBP}		0.30	0.07	<0.001
	β_{SBP}	β_{MO}	0.08	0.04	0.06
	β_{PP}		-0.09	0.07	0.18
	β_{DBP}		0.14	0.06	0.02
	β_{SBP}	$\beta_{Migraine}$	0.06	0.04	0.11
MR-Egger	β_{PP}		-0.08	0.07	0.25
	β_{DBP}		0.14	0.14	0.34
	β_{SBP}	β_{MA}	0.06	0.09	0.51
	β_{PP}		-0.19	0.14	0.18

β_{DBP}		0.30	0.1	0.01
β_{SBP}	β_{MO}	0.10	0.08	0.16
β_{PP}		-0.21	0.15	0.16

Abbreviations: SE: standard error; IVW: inverse-variance weighted; MR-Egger: Egger regression; DBP: diastolic blood pressure; SBP: systolic blood pressure; PP: pulse pressure; MA: migraine with aura; MO: migraine without aura.

All sensitivity analyses were conducted using the corresponding models implemented in R package “MendelianRandomization”.

Supplementary Table 25 **Mendelian randomization (MR) analysis for five instruments from antihypertensive medications genetic targets with migraine and MR power calculation**

Exposure	Outcome	SNP	CHR	effect	MAF	*R2*	*R2*.TOTAL	MR.effect	*P*	MR.POWER
DBP	Anymigraine	rs8077276	17	-0.02	0.38	0.0001	0.0008	0.20	0.98	0.25
		rs2484294	10	0.03	0.27	0.0003				
		rs1888693	10	0.02	0.34	0.0002				
		rs3821843	3	0.02	0.32	0.0001				
		rs2239046	12	0.01	0.32	0.0000				
SBP		rs8077276	17	-0.02	0.38	0.0002	0.0011	0.08	0.89	0.1
		rs2484294	10	0.03	0.27	0.0004				
		rs1888693	10	0.03	0.34	0.0003				
		rs3821843	3	0.02	0.32	0.0002				
		rs2239046	12	0.01	0.32	0.0001				
PP		rs8077276	17	-0.01	0.38	0.0000	0.0003	-0.11	0.68	0.07
		rs2484294	10	0.01	0.27	0.0000				
		rs1888693	10	0.01	0.34	0.0001				
		rs3821843	3	0.01	0.32	0.0001				
		rs2239046	12	0.01	0.32	0.0000				

Abbreviations: SNP: single nucleotide polymorphisms; CHR: chromosome; MAF: minor allele frequency; R2: proportion of variance explained for the association between the corresponding SNP and the exposure variable; R2.TOTAL: proportion of variance explained for the association between all the five SNPs and the exposure variable; DBP: diastolic blood pressure; MR.POWER: estimated power in mendelian randomization; SBP: systolic blood pressure; PP: pulse pressure; MA: migraine with aura; MO: migraine without aura.

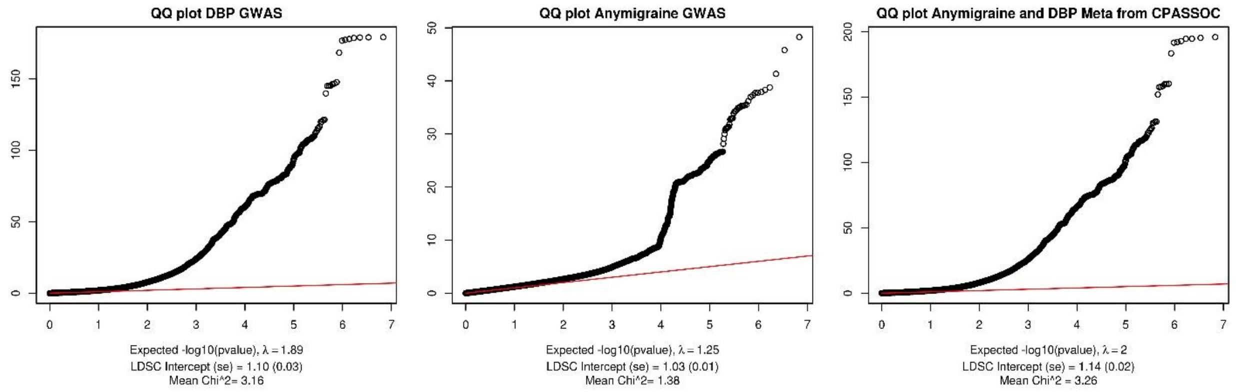

Supplementary Figure 1 **Quantile-quantile (QQ) plot for diastolic blood pressure (DBP), migraine, and cross-trait meta-analysis between DBP and migraine.**

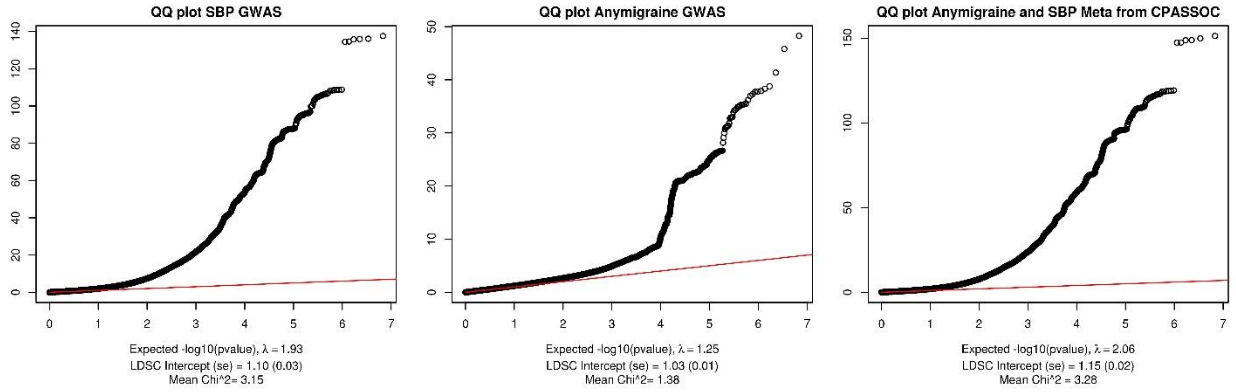

Supplementary Figure 2 **Quantile-quantile (QQ) plot for systolic blood pressure (SBP), migraine, and cross-trait meta-analysis between SBP and migraine.**

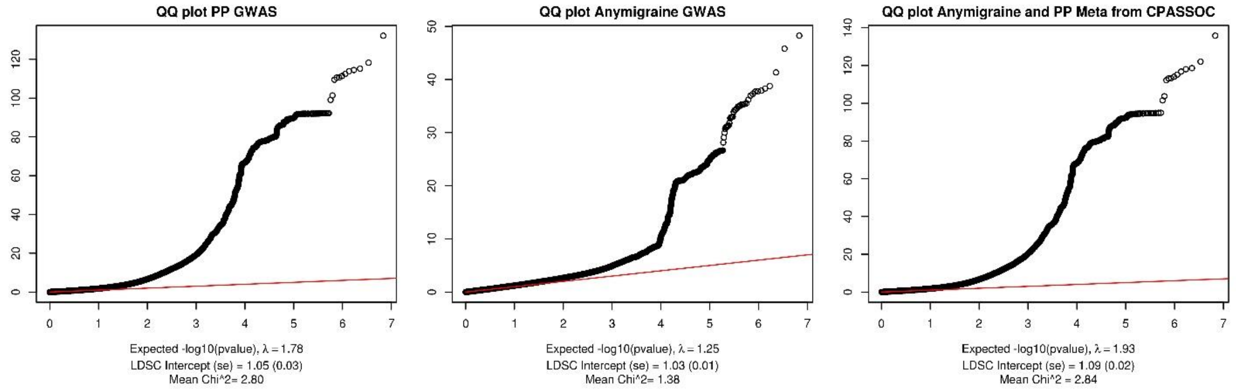

Supplementary Figure 3 **Quantile-quantile (QQ) plot for pulse pressure (PP), migraine, and cross-trait meta-analysis between SBP and migraine.**

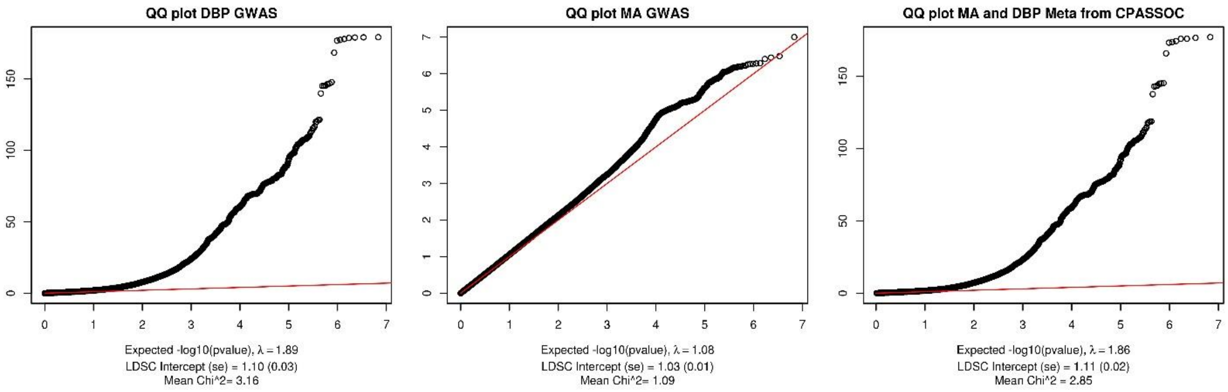

Supplementary Figure 4 Quantile-quantile (QQ) plot for diastolic blood pressure (DBP), migraine with aura (MA), and cross-trait meta-analysis between DBP and MA.

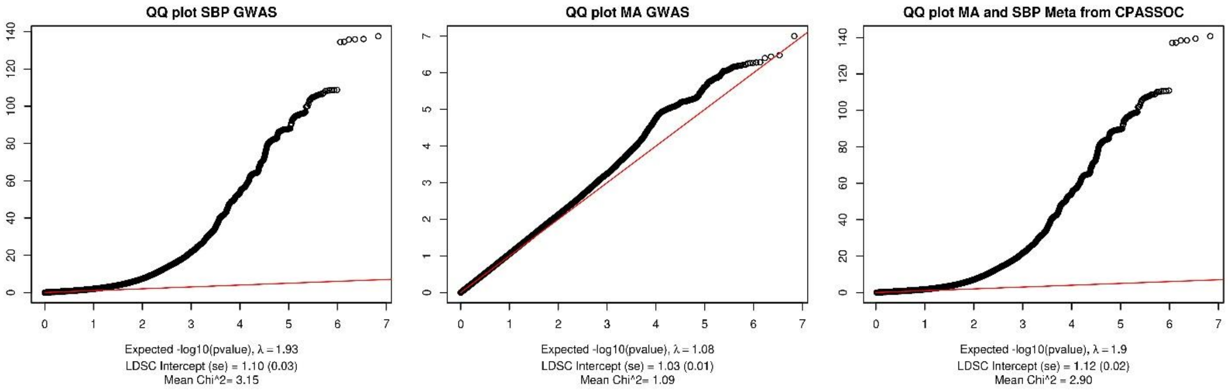

Supplementary Figure 5 Quantile-quantile (QQ) plot for systolic blood pressure (SBP), migraine with aura (MA), and cross-trait meta-analysis between SBP and MA.

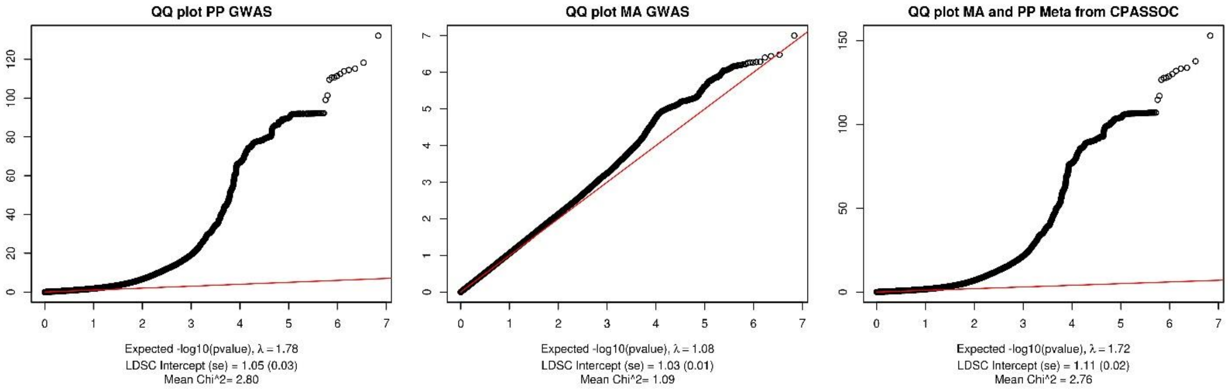

Supplementary Figure 6 Quantile-quantile (QQ) plot for pulse pressure (PP), migraine with aura (MA), and cross-trait meta-analysis between PP and MA.

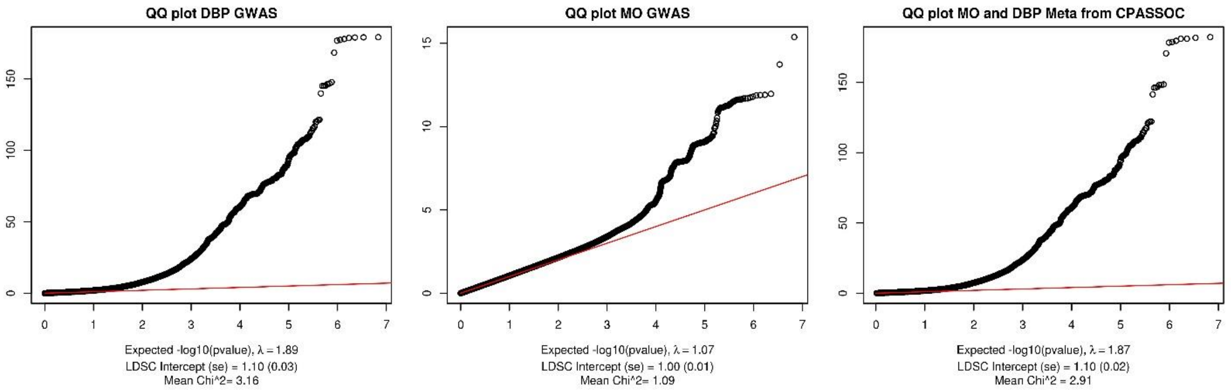

Supplementary Figure 7 **Quantile-quantile (QQ) plot for diastolic blood pressure (DBP), migraine without aura (MO), and cross-trait meta-analysis between DBP and MO.**

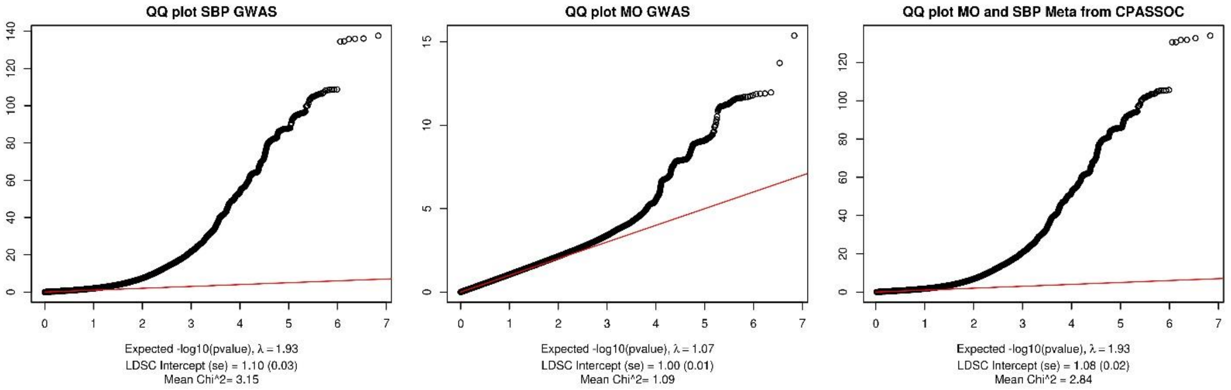

Supplementary Figure 8 **Quantile-quantile (QQ) plot for systolic blood pressure (SBP), migraine without aura (MO), and cross-trait meta-analysis between SBP and MO.**

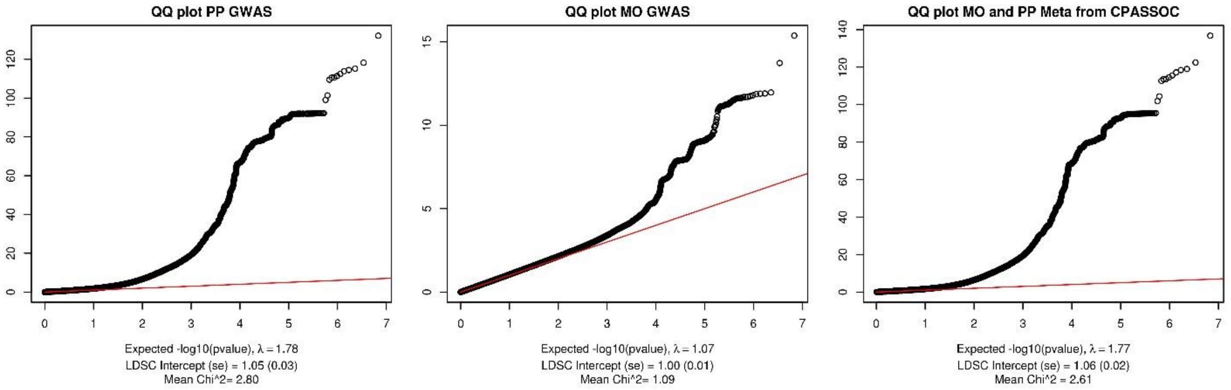

Supplementary Figure 9 **Quantile-quantile (QQ) plot for pulse pressure (PP), migraine without aura (MO), and cross-trait meta-analysis between PP and MO.**

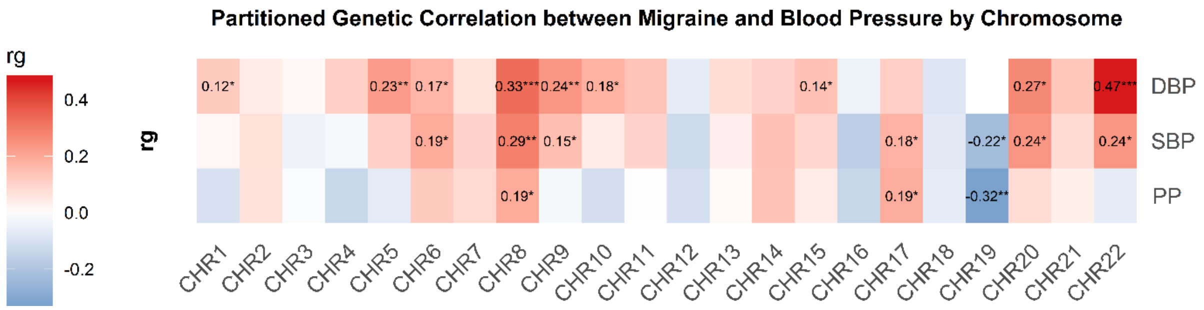

Supplementary Figure 10 Partitioned genetic correlation between migraine and blood pressure by different chromosomes. Colors represent the estimated annotation specific genetic correlation between migraine and BP traits (DBP, SBP, and PP) using GNOVA, red for positive genetic correlation and blue for negative genetic correlation at the corresponding annotation. The significant effects (P<0.05) are labeled with r_g. The nominally significant effects are labeled with "*" ($P < 0.05$), "**" ($P < 0.01$), and "***" ($P < 0.001$).

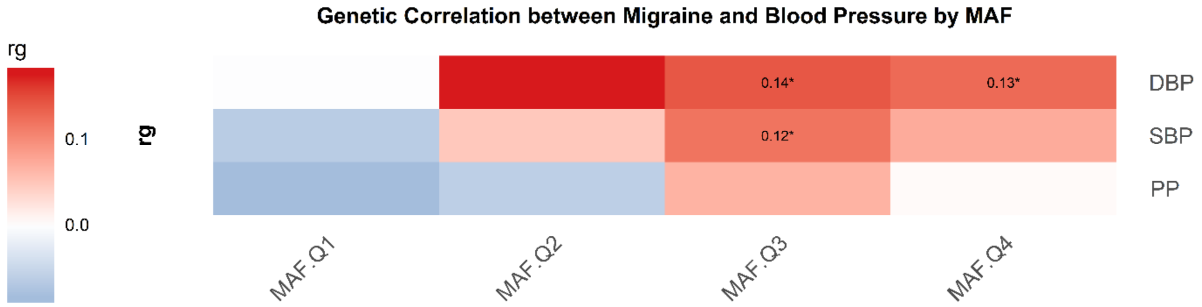

Supplementary Figure 11 Partitioned genetic correlation between migraine and blood pressure by minor allele frequency (MAF) quartiles. Colors represent the estimated annotation specific genetic correlation between migraine and BP traits (DBP, SBP, and PP) using GNOVA, red for positive genetic correlation and blue for negative genetic correlation at the corresponding annotation. The significant effects (P<0.05) are labeled with r_g. The nominally significant effects are labeled with "*" ($P < 0.05$), "**" ($P < 0.01$), and "***" ($P < 0.001$).

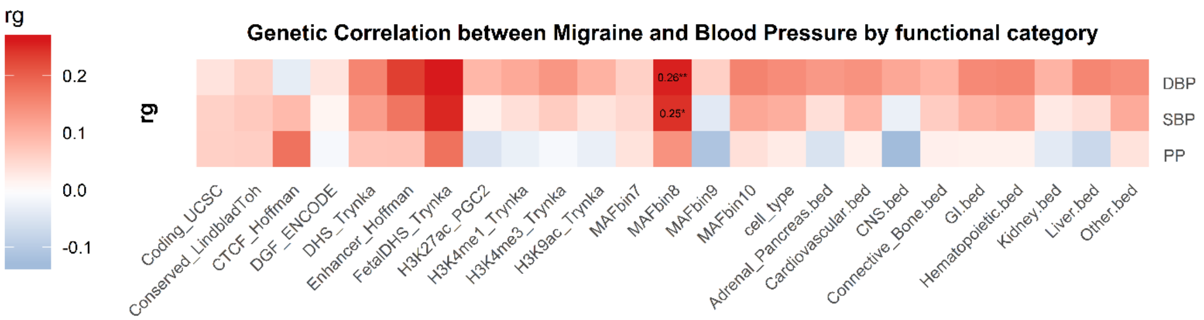

Supplementary Figure 12 Partitioned genetic correlation between migraine and blood pressure by different functional categories. Colors represent the estimated annotation specific genetic correlation between migraine and BP traits (DBP, SBP, and PP) using GNOVA, red for positive genetic correlation and blue for negative genetic correlation at the corresponding annotation. The significant effects (P<0.05) are labeled with r_g. The nominally significant effects are labeled with "*" ($P < 0.05$), "**" ($P < 0.01$), and "***" ($P < 0.001$).

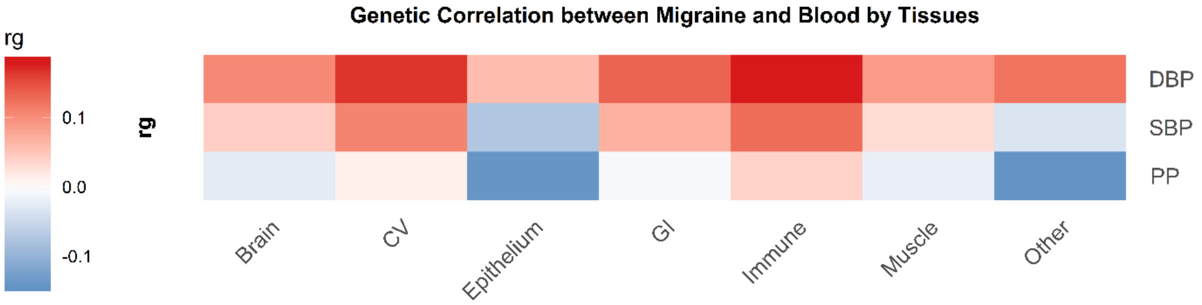

Supplementary Figure 13 Partitioned genetic correlation between migraine and blood pressure by seven tissues based on GenoSkyline-Plus annotations. Colors represent the estimated annotation specific genetic correlation between migraine and BP traits (DBP, SBP, and PP) using GNOVA, red for positive genetic correlation and blue for negative genetic correlation at the corresponding annotation. The significant effects (P<0.05) are labeled with r_g. The nominally significant effects are labeled with "*" ($P < 0.05$), "**" ($P < 0.01$), and "***" ($P < 0.001$).

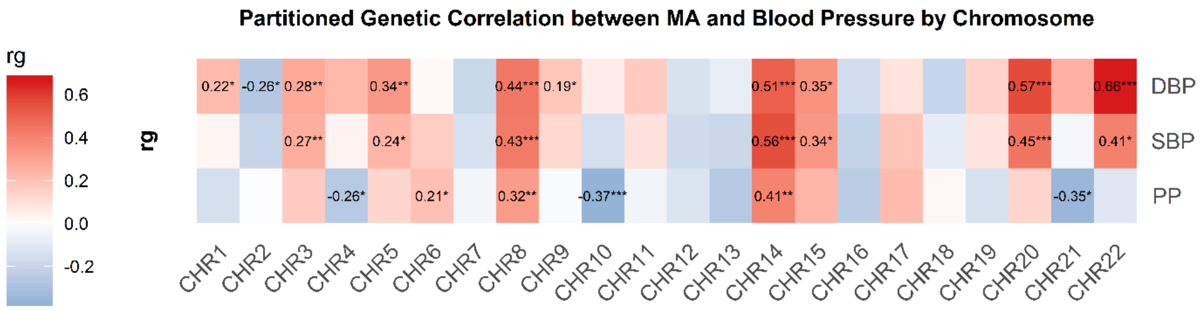

Supplementary Figure 14 Partitioned genetic correlation between migraine with aura (MA) and blood pressure by different chromosomes. Colors represent the estimated annotation specific genetic correlation between migraine with aura (MA) and BP traits (DBP, SBP, and PP) using GNOVA, red for positive genetic correlation and blue for negative genetic correlation at the corresponding annotation. The significant effects (P<0.05) are labeled with r_g. The nominally significant effects are labeled with "*" ($P < 0.05$), "**" ($P < 0.01$), and "***" ($P < 0.001$).

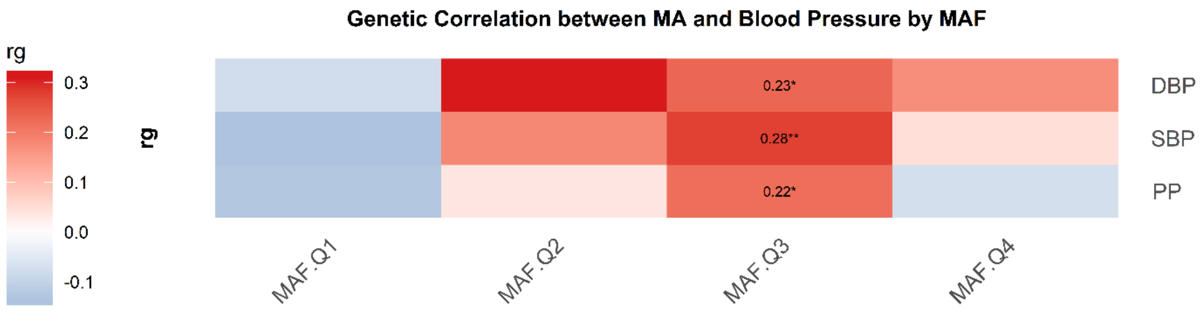

Supplementary Figure 15 Partitioned genetic correlation between migraine with aura (MA) and blood pressure by minor allele frequency (MAF) quartiles. Colors represent the estimated annotation specific genetic correlation between migraine with aura (MA) and BP traits (DBP, SBP, and PP) using GNOVA, red for positive genetic correlation and blue for negative genetic correlation at the corresponding annotation. The significant effects (P<0.05) are labeled with r_g. The nominally significant effects are labeled with "*" ($P < 0.05$), "**" ($P < 0.01$), and "***" ($P < 0.001$).

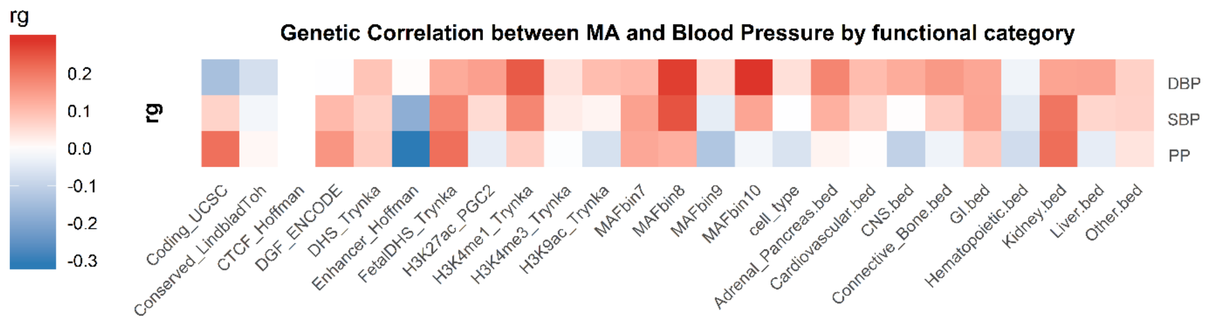

Supplementary Figure 16 Partitioned genetic correlation between migraine with aura (MA) and blood pressure by different functional categories. Colors represent the estimated annotation specific genetic correlation between migraine with aura (MA) and BP traits (DBP, SBP, and PP) using GNOVA, red for positive genetic correlation and blue for negative genetic correlation at the corresponding annotation. The significant effects (P<0.05) are labeled with r_g. The nominally significant effects are labeled with "*" ($P < 0.05$), "**" ($P < 0.01$), and "***" ($P < 0.001$).

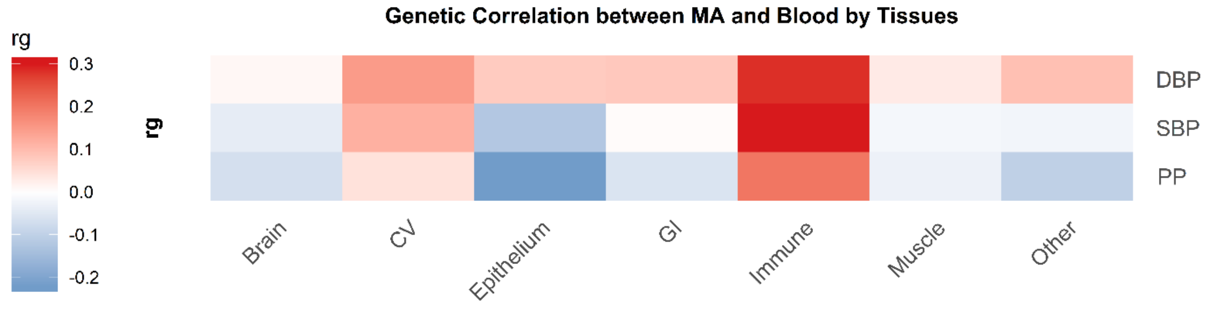

Supplementary Figure 17 Partitioned genetic correlation between migraine with aura (MA) and blood pressure by seven tissues based on GenoSkyline-Plus annotations. Colors represent the estimated annotation specific genetic correlation between migraine with aura (MA) and BP traits (DBP, SBP, and PP) using GNOVA, red for positive genetic correlation and blue for negative genetic correlation at the corresponding annotation. The significant effects (P<0.05) are labeled with r_g. The nominally significant effects are labeled with "*" ($P < 0.05$), "**" ($P < 0.01$), and "***" ($P < 0.001$).

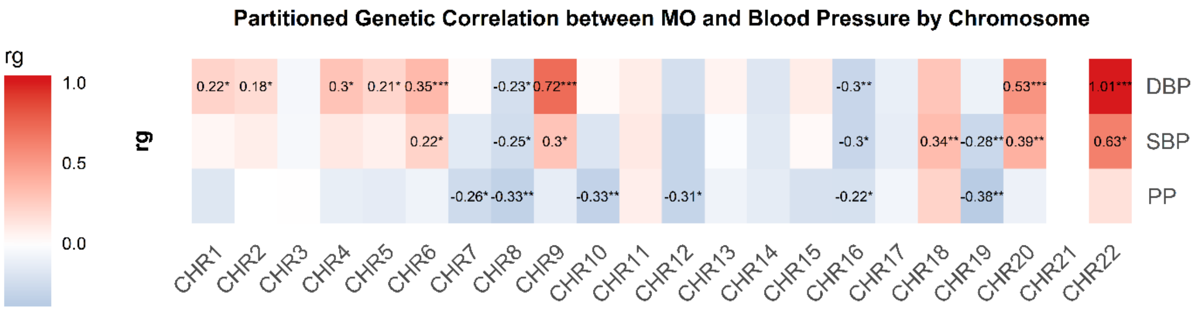

Supplementary Figure 18 **Partitioned genetic correlation between migraine without aura (MO) and blood pressure by different chromosomes.** Colors represent the estimated annotation specific genetic correlation between migraine without aura (MO) and BP traits (DBP, SBP, and PP) using GNOVA, red for positive genetic correlation and blue for negative genetic correlation at the corresponding annotation. The significant effects (P<0.05) are labeled with r_g. The nominally significant effects are labeled with "*" ($P < 0.05$), "**" ($P < 0.01$), and "***" ($P < 0.001$).

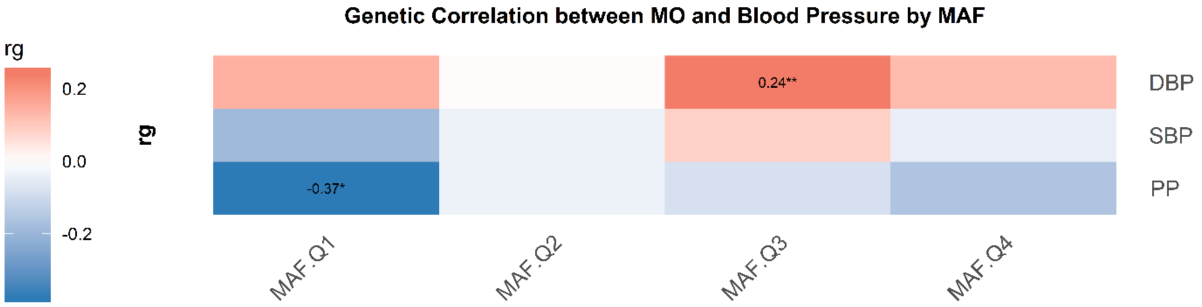

Supplementary Figure 19 **Partitioned genetic correlation between migraine without aura (MO) and blood pressure by minor allele frequency (MAF) quartiles.** Colors represent the estimated annotation specific genetic correlation between migraine without aura (MO) and BP traits (DBP, SBP, and PP) using GNOVA, red for positive genetic correlation and blue for negative genetic correlation at the corresponding annotation. The significant effects (P<0.05) are labeled with r_g. The nominally significant effects are labeled with "*" ($P < 0.05$), "**" ($P < 0.01$), and "***" ($P < 0.001$).

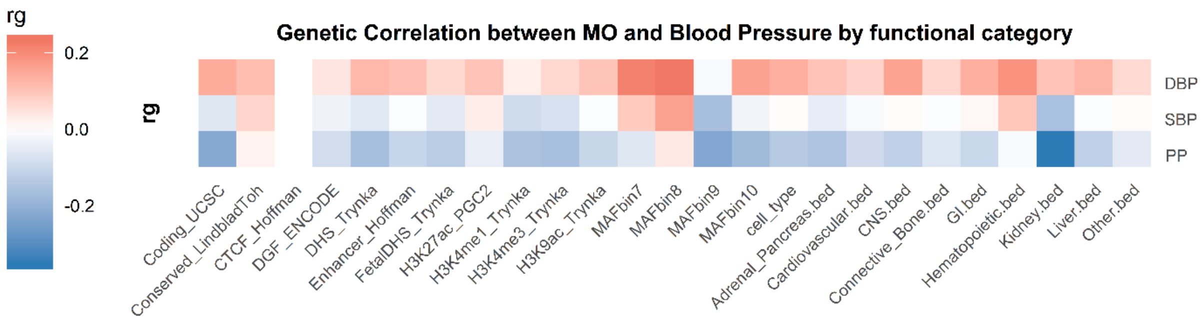

Supplementary Figure 20 Partitioned genetic correlation between migraine without aura (MO) and blood pressure by different functional categories. Colors represent the estimated annotation specific genetic correlation between migraine without aura (MO) and BP traits (DBP, SBP, and PP) using GNOVA, red for positive genetic correlation and blue for negative genetic correlation at the corresponding annotation. The significant effects (P<0.05) are labeled with r_g. The nominally significant effects are labeled with "*" ($P < 0.05$), "**" ($P < 0.01$), and "***" ($P < 0.001$).

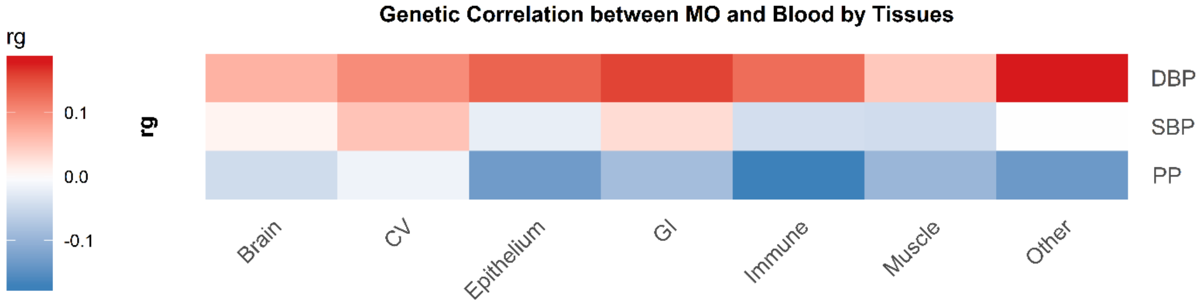

Supplementary Figure 21 Partitioned genetic correlation between migraine without aura (MO) and blood pressure by seven tissues based on GenoSkyline-Plus annotations. Colors represent the estimated annotation specific genetic correlation between migraine without aura (MO) and BP traits (DBP, SBP, and PP) using GNOVA, red for positive genetic correlation and blue for negative genetic correlation at the corresponding annotation. The significant effects (P<0.05) are labeled with r_g. The nominally significant effects are labeled with "*" ($P < 0.05$), "**" ($P < 0.01$), and "***" ($P < 0.001$).

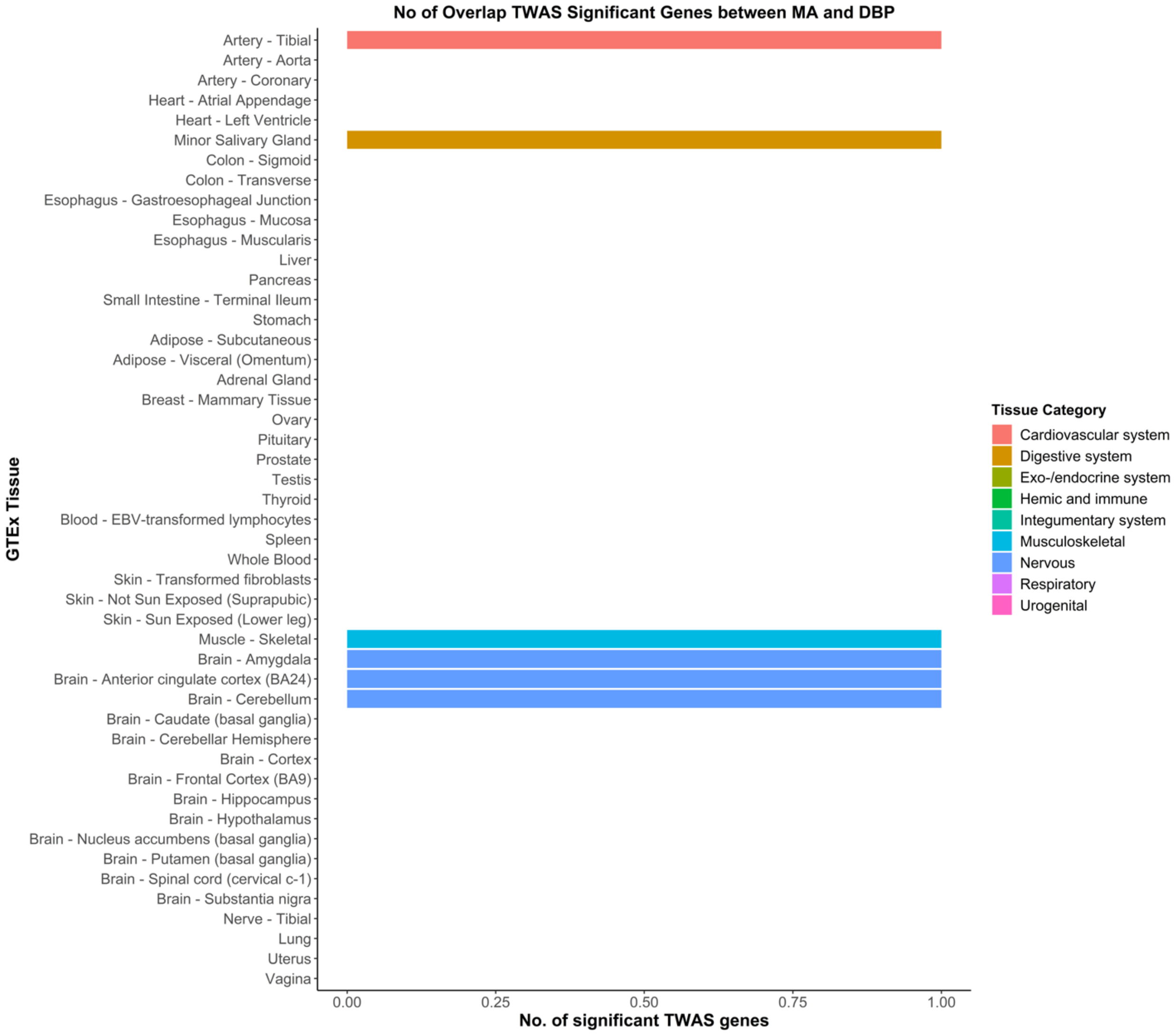

Supplementary Figure 22 The number of shared TWAS significant genes between migraine with aura (MA) and diastolic blood pressure (DBP) across 48 GTEx tissues. Colors represent different tissue categories.

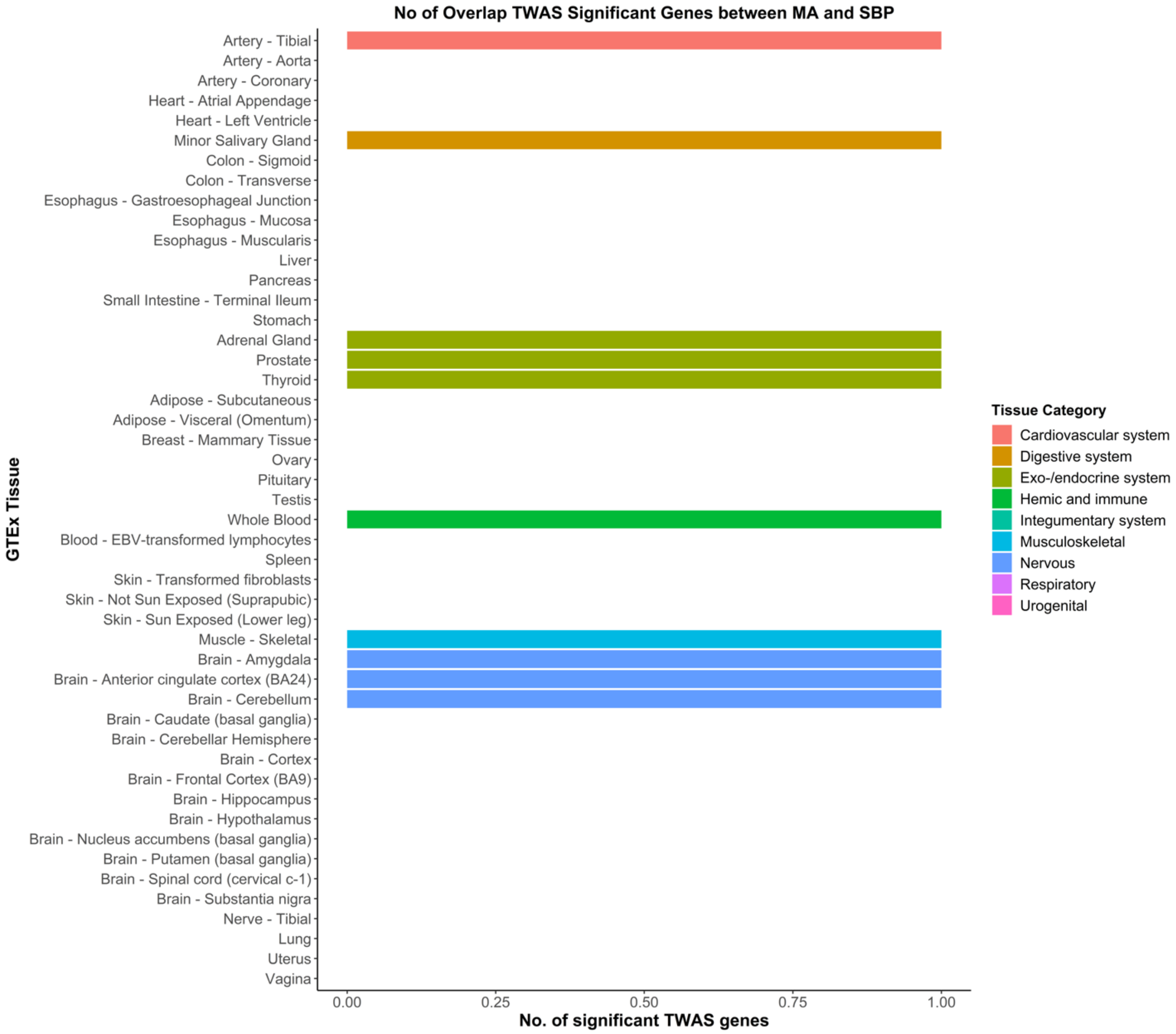

Supplementary Figure 23 **The number of shared TWAS significant genes between migraine with aura (MA) and systolic blood pressure (SBP) across 48 GTEx tissues.** Colors represent different tissue categories.

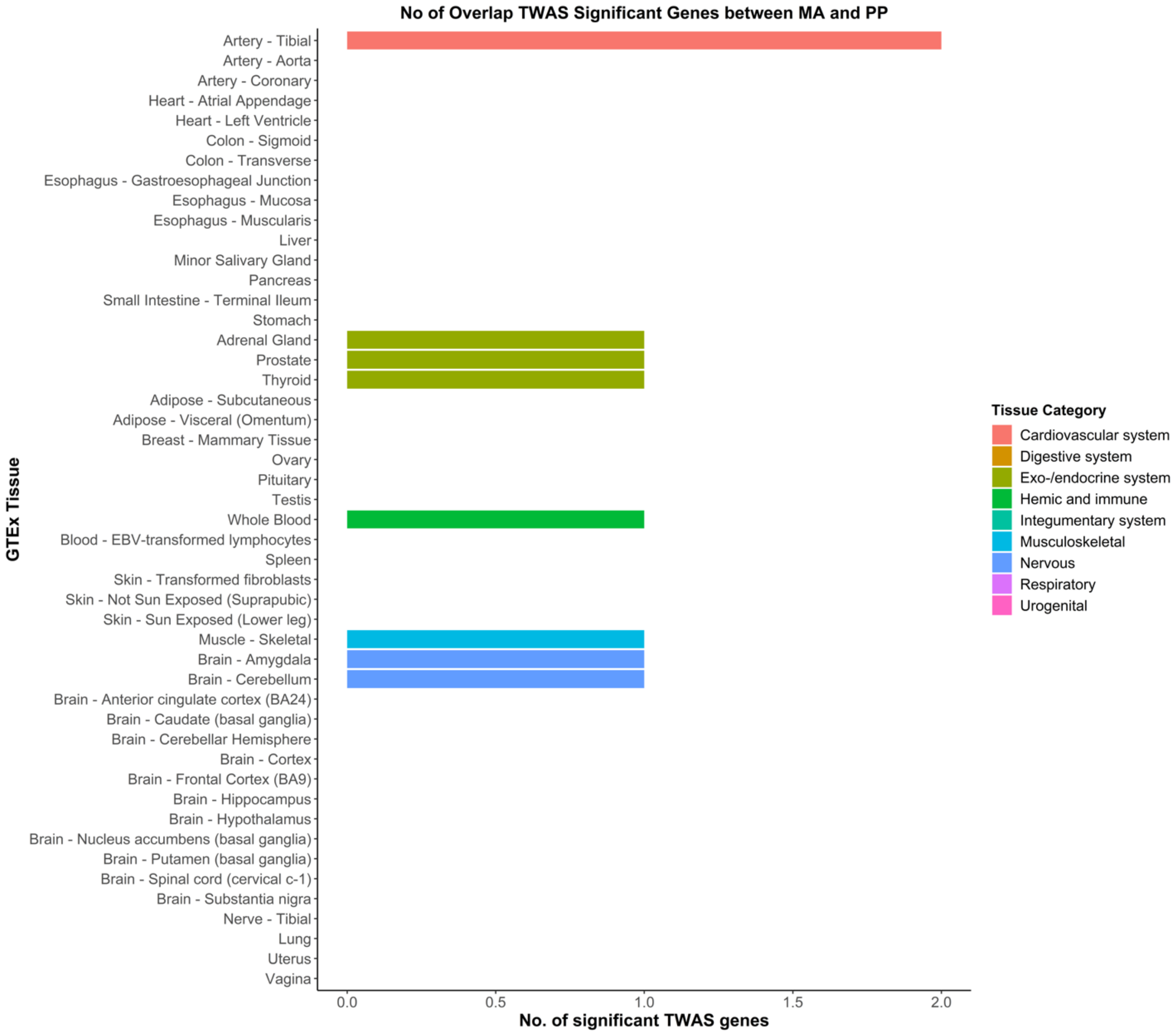

Supplementary Figure 24 The number of shared TWAS significant genes between migraine with aura (MA) and pulse pressure (PP) across 48 GTEx tissues. Colors represent different tissue categories.

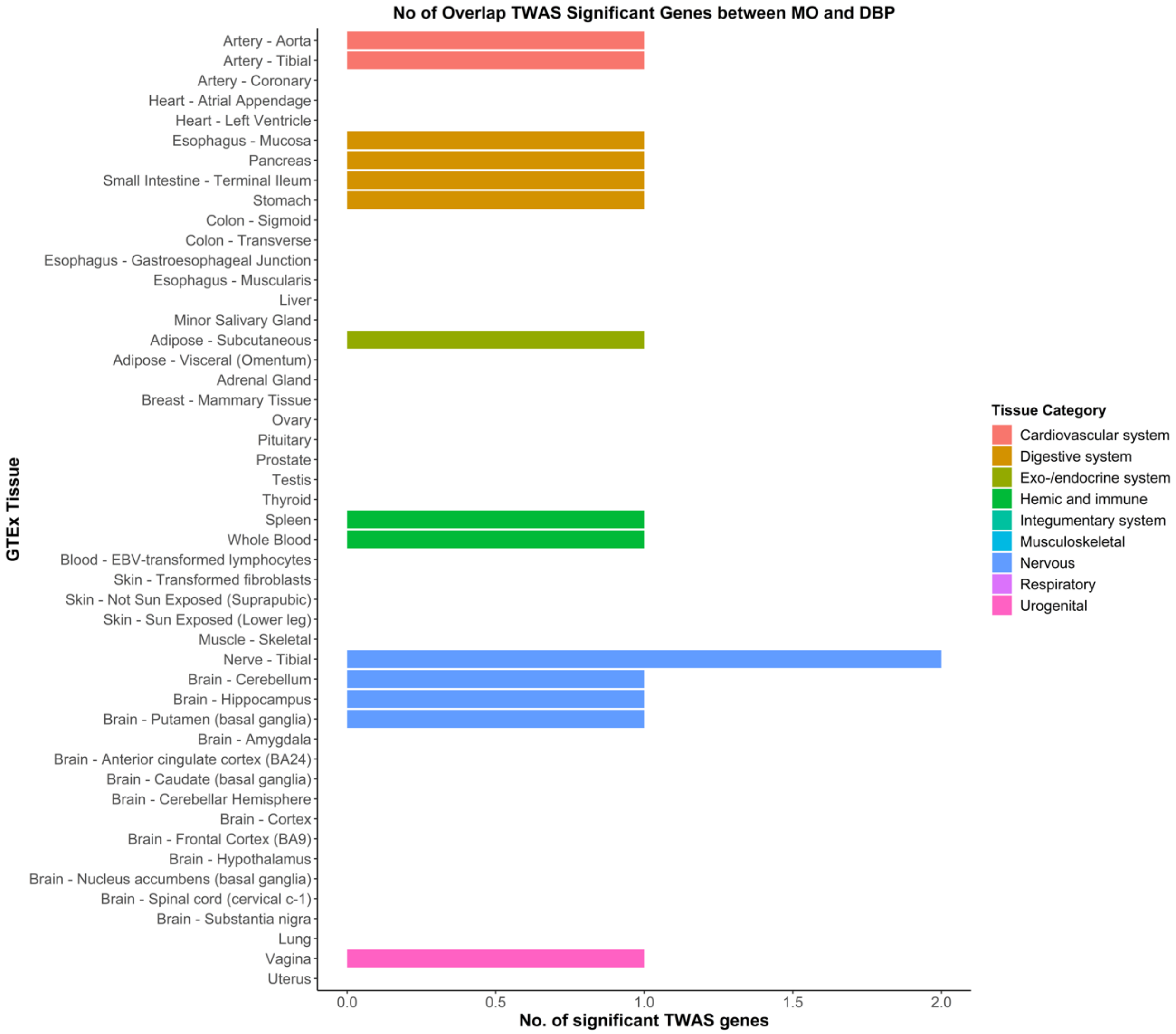

Supplementary Figure 25 The number of shared TWAS significant genes between migraine without aura (MO) and diastolic blood pressure (DBP) across 48 GTEx tissues. Colors represent different tissue categories.

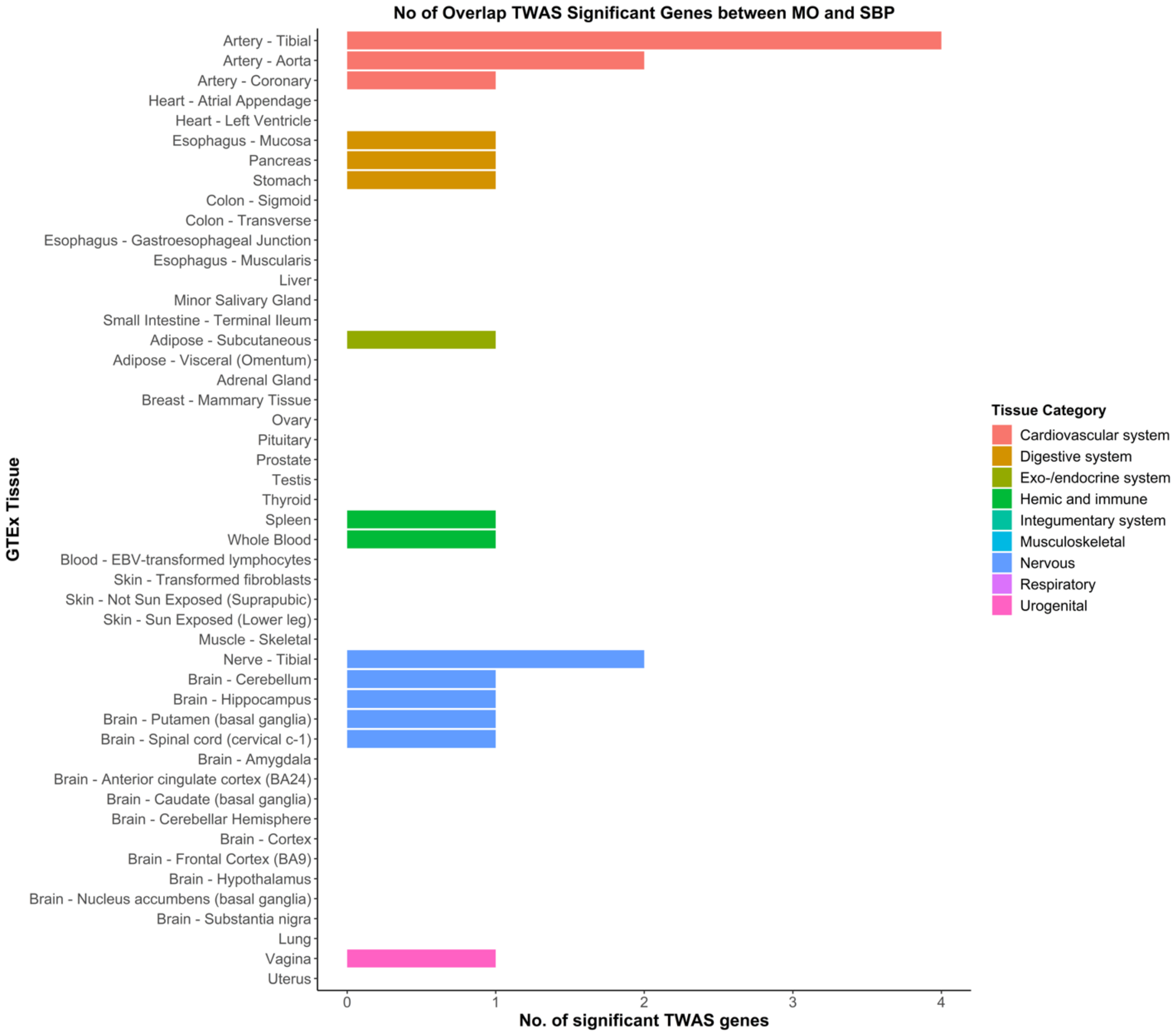

Supplementary Figure 26 The number of shared TWAS significant genes between migraine without aura (MO) and systolic blood pressure (SBP) across 48 GTEx tissues. Colors represent different tissue categories.

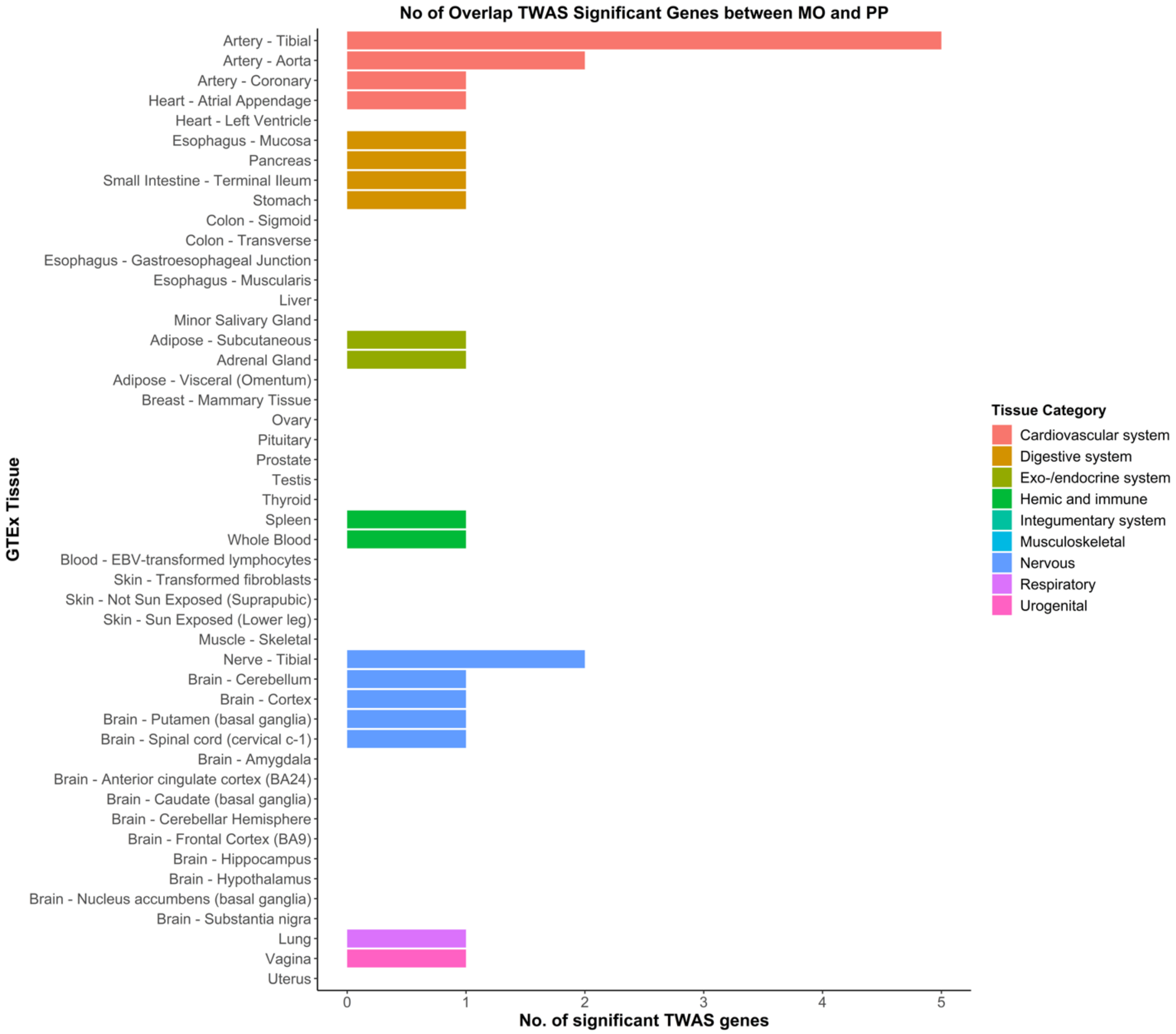

Supplementary Figure 27 The number of shared TWAS significant genes between migraine without aura (MO) and pulse pressure (PP) across 48 GTEx tissues. Colors represent different tissue categories.

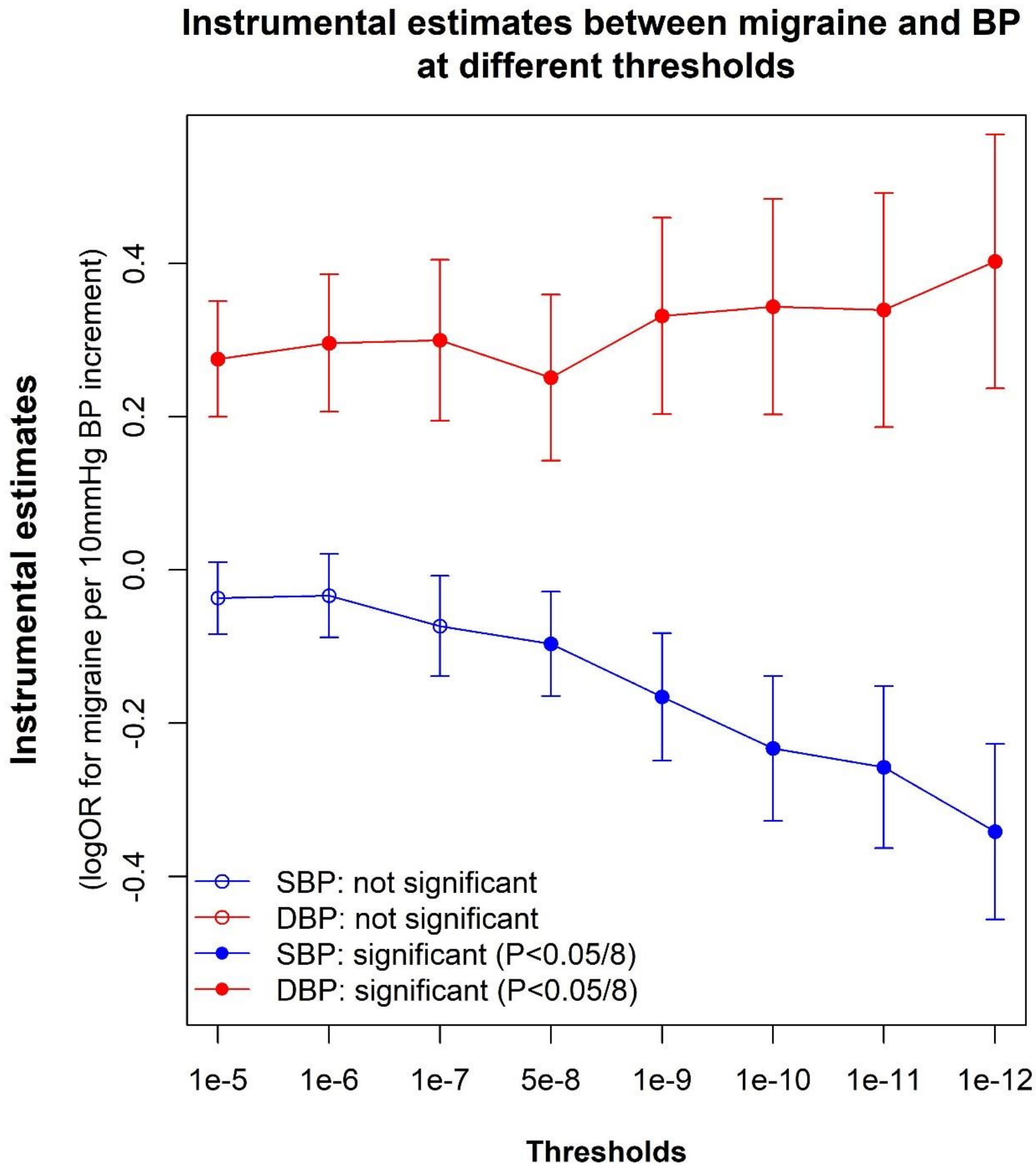

Supplementary Figure 28 Instrumental estimates for migraine per 10mmHg increment of SBP and DBP by restricting SNPs that are not associated with DBP and SBP (*P*>0.05). For SBP, SNPs that were related with DBP (P_{GWAS} for DBP≤0.05) were removed and then used the summary statistics for the remaining SNPs to run GSMR at eight different thresholds (1e-5, 1e-6, 1e-7, 5e-8, 1e-9, 1e-10, 1e-11, and 1e-12). For DBP, SNPs that were related with SBP (P_{GWAS} for SBP≤0.05) were removed and conducted GSMR for DBP.

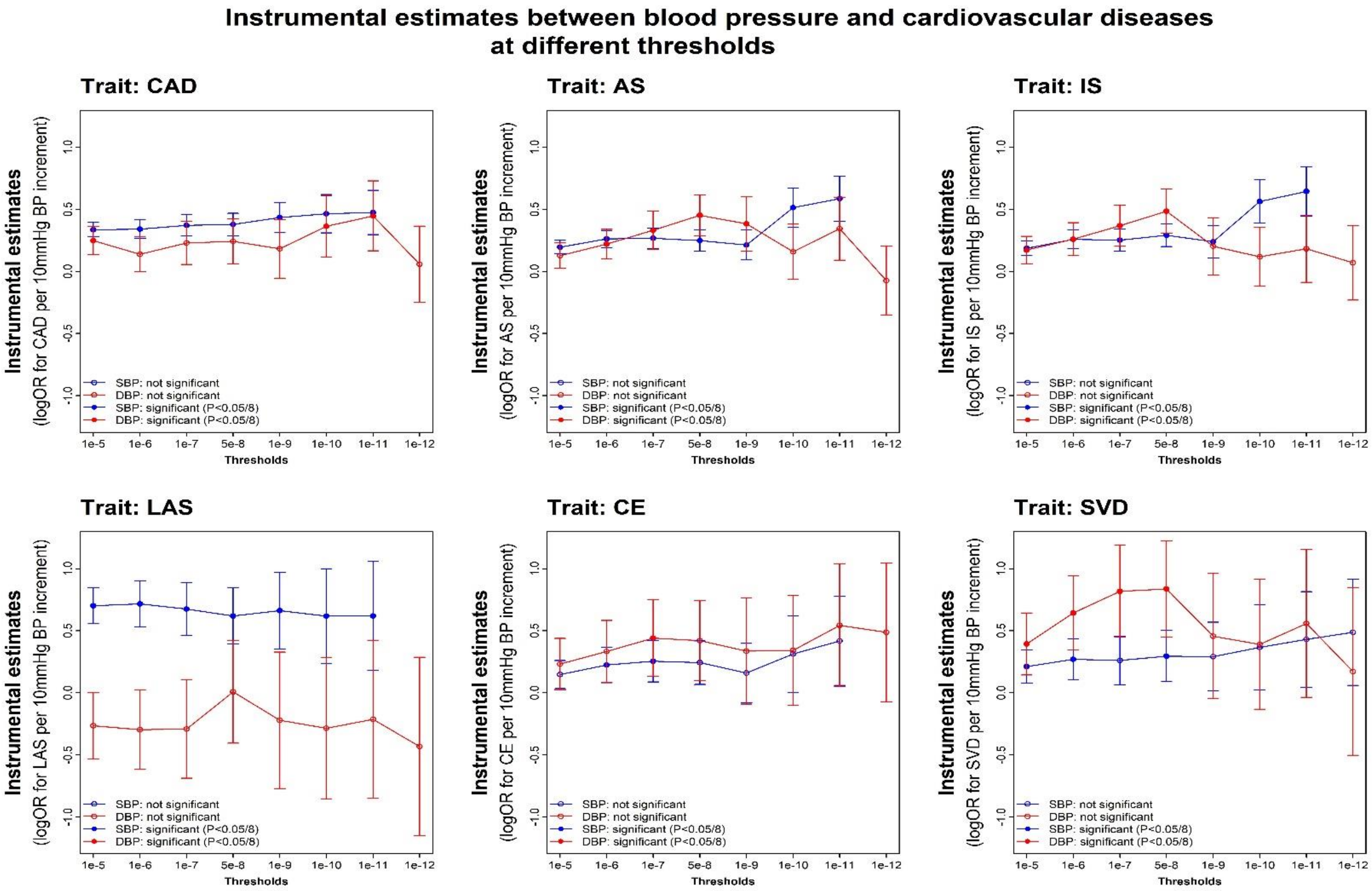

Supplementary Figure 29 Instrumental estimates for cardiovascular comorbidities of migraine per 10mmHg increment of SBP and DBP by restricting SNPs that are not associated with DBP and SBP (*P*>0.05). For SBP, SNPs that were related with DBP (P_{GWAS} for DBP≤0.05) were removed and then used the summary statistics for the remaining SNPs to run GSMR at eight different thresholds (1e-5, 1e-6, 1e-7, 5e-8, 1e-9, 1e-10, 1e-11, and 1e-12). For DBP, SNPs that were related with SBP (P_{GWAS} for SBP≤0.05) were removed and conducted GSMR for DBP. CAD: coronary artery disease; AS: any stroke; IS: ischemic stroke; LAS: large artery stroke; CE: cardioembolic stroke; SVD: small vessel stroke.

Author list for International Headache Genetics Consortium (IHGC) on paper "Meta-analysis of 375,000 individuals identifies 38 susceptibility loci for migraine", Nature Genetics, 2016, 48(8):856-66. doi: 10.1038/ng.3598. [PubMed ID: 27322543].

Padhraig Gormley*[,1,2,3,4], Verneri Anttila*[,2,3,5], Bendik S Winsvold[6,7,8], Priit Palta[9], Tonu Esko[2,10,11], Tune H. Pers[2,11,12,13], Kai-How Farh[2,5,14], Ester Cuenca-Leon[1,2,3,15], Mikko Muona[9,16,17,18], Nicholas A Furlotte[19], Tobias Kurth[20,21], Andres Ingason[22], George McMahon[23], Lannie Ligthart[24], Gisela M Terwindt[25], Mikko Kallela[26], Tobias M Freilinger[27,28], Caroline Ran[29], Scott G Gordon[30], Anine H Stam[25], Stacy Steinberg[22], Guntram Borck[31], Markku Koiranen[32], Lydia Quaye[33], Hieab HH Adams[34,35], Terho Lehtimäki[36], Antti-Pekka Sarin[9], Juho Wedenoja[37], David A Hinds[19], Julie E Buring[21,38], Markus Schürks[39], Paul M Ridker[21,38], Maria Gudlaug Hrafnsdottir[40], Hreinn Stefansson[22], Susan M Ring[23], Jouke-Jan Hottenga[24], Brenda WJH Penninx[41], Markus Färkkilä[26], Ville Artto[26], Mari Kaunisto[9], Salli Vepsäläinen[26], Rainer Malik[27], Andrew C Heath[42], Pamela A F Madden[42], Nicholas G Martin[30], Grant W Montgomery[30], Mitja Kurki[1,2,3], Mart Kals[10], Reedik Mägi[10], Kalle Pärn[10], Eija Hämäläinen[9], Hailiang Huang[2,3,5], Andrea E Byrnes[2,3,5], Lude Franke[43], Jie Huang[4], Evie Stergiakouli[23], Phil H Lee[1,2,3], Cynthia Sandor[44], Caleb Webber[44], Zameel Cader[45,46], Bertram Muller-Myhsok[47], Stefan Schreiber[48], Thomas Meitinger[49], Johan G Eriksson[50,51], Veikko Salomaa[51], Kauko Heikkilä[52], Elizabeth Loehrer[34,53], Andre G Uitterlinden[54], Albert Hofman[34], Cornelia M van Duijn[34], Lynn Cherkas[33], Linda M. Pedersen[6], Audun Stubhaug[55,56], Christopher S Nielsen[55,57], Minna Männikkö[32], Evelin Mihailov[10], Lili Milani[10], Hartmut Göbel[58], Ann-Louise Esserlind[59], Anne Francke Christensen[59], Thomas Folkmann Hansen[60], Thomas Werge[61,62,63], International Headache Genetics Consortium[64], Jaakko Kaprio[9,65,66], Arpo J Aromaa[51], Olli Raitakari[67,68], M Arfan Ikram[34,35,68], Tim Spector[33], Marjo-Riitta Järvelin[32,70,71,72], Andres Metspalu[10], Christian Kubisch[73], David P Strachan[74], Michel D Ferrari[25], Andrea C Belin[29], Martin Dichgans[27,75], Maija Wessman[9,16], Arn MJM van den Maagdenberg[25,76], John-Anker Zwart[6,7,8], Dorret I Boomsma[24], George Davey Smith[23], Kari Stefansson[22,77], Nicholas Eriksson[19], Mark J Daly[2,3,5], Benjamin M Neale[§,2,3,5], Jes Olesen[§,59], Daniel I Chasman[§,21,38], Dale R Nyholt[§,78], and Aarno Palotie[§,1,2,3,4,5,9,79].

Affiliations at the time the manuscript was published:

[1]Psychiatric and Neurodevelopmental Genetics Unit, Massachusetts General Hospital and Harvard Medical School, Boston, USA. [2]Medical and Population Genetics Program, Broad Institute of MIT and Harvard, Cambridge, USA. [3]Stanley Center for Psychiatric Research, Broad Institute of MIT and Harvard, Cambridge, USA. [4]Wellcome Trust Sanger Institute, Wellcome Trust Genome Campus, Hinxton, UK. [5]Analytic and Translational Genetics Unit, Massachusetts General Hospital and Harvard Medical School, Boston, USA. [6]FORMI, Oslo University Hospital, P.O. 4956 Nydalen, 0424 Oslo, Norway. [7]Department of Neurology, Oslo University Hospital, P.O. 4956 Nydalen, 0424 Oslo, Norway. [8]Institute of Clinical Medicine, University of Oslo, P.O. 1171 Blindern, 0318 Oslo, Norway. [9]Institute for Molecular Medicine Finland (FIMM), University of Helsinki, Helsinki, Finland. [10]Estonian Genome Center, University of Tartu, Tartu, Estonia. [11]Division of Endocrinology, Boston Children's Hospital, Boston, USA. [12]Statens Serum Institut, Dept of Epidemiology Research, Copenhagen, Denmark. [13]Novo Nordisk Foundation Center for Basic Metabolic Research, University of Copenhagen, Copenhagen, Denmark. [14]Illumina, 5200 Illumina Way, San Diego, USA. [15]Vall d'Hebron Research Institute, Pediatric Neurology, Barcelona, Spain. [16]Folkhälsan Institute of Genetics, Helsinki, Finland, FI-00290. [17]Neuroscience Center, University of Helsinki, Helsinki, Finland, FI-00014. [18]Research Programs Unit, Molecular Neurology, University of Helsinki, Helsinki, Finland, FI-00014. [19]23andMe, Inc., 899 W. Evelyn Avenue, Mountain View, CA, USA. [20]Inserm Research Center for Epidemiology and Biostatistics (U897), University of Bordeaux, 33076 Bordeaux, France. [21]Division of Preventive Medicine, Brigham and Women's Hospital, Boston MA 02215. [22]deCODE Genetics, 101 Reykjavik, Iceland. [23]Medical Research Council (MRC) Integrative Epidemiology Unit, University of Bristol, Bristol, UK. [24]VU University Amsterdam, Department of Biological Psychology, Amsterdam, the Netherlands, 1081 BT. [25]Leiden University Medical Centre, Department of Neurology, Leiden, The Netherlands, PO Box 9600, 2300 RC. [26]Department of Neurology, Helsinki University Central Hospital, Haartmaninkatu 4, 00290 Helsinki, Finland. [27]Institute for Stroke and Dementia Research, Klinikum der Universtität München, Ludwig-Maximilians-Universität München, Feodor-Lynen-Str. 17, 81377 Munich Germany. [28]Department of Neurology and Epileptology, Hertie Institute for Clincal Brain Research, University of Tuebingen. [29]Karolinska Institutet, Department of Neuroscience, 171 77 Stockholm, Sweden. [30]Department of Genetics and Computational Biology, QIMR Berghofer Medical Research Institute, 300 Herston Road, Brisbane, QLD 4006, Australia. [31]Ulm University, Institute of Human Genetics, 89081 Ulm, Germany. [32]University of Oulu, Center for Life Course Epidemiology and Systems Medicine, Oulu, Finland, Box 5000, Fin-90014 University of Oulu. [33]Department of Twin Research and Genetic Epidemiology, King's College London, London, UK. [34]Dept of Epidemiology, Erasmus University Medical Center, Rotterdam, the Netherlands, 3015 CN. [35]Dept of Radiology, Erasmus University Medical Center, Rotterdam, the Netherlands, 3015 CN. [36]Department of Clinical Chemistry, Fimlab Laboratories, and School of Medicine, University of Tampere, Tampere, Finland, 33520. [37]Department of Public Health, University of Helsinki, Helsinki, Finland. [38]Harvard Medical School, Boston MA 02115. [39]University Duisburg Essen, Essen, Germany. [40]Landspitali University Hospital, 101 Reykjavik, Iceland. [41]VU University Medical Centre, Department of Psychiatry, Amsterdam, the Netherlands, 1081 HL. [42]Department of Psychiatry, Washington University School of Medicine, 660 South Euclid, CB 8134, St. Louis, MO 63110, USA. [43]University Medical Center Groningen, University of Groningen, Groningen, The Netherlands, 9700RB. [44]MRC Functional Genomics Unit, Department of Physiology, Anatomy & Genetics, Oxford University, UK. [45]Nuffield Department of Clinical Neuroscience, University of Oxford, UK. [46]Oxford Headache Centre, John Radcliffe Hospital, Oxford, UK. [47]Max-Planck-Institute of Psychiatry, Munich, Germany. [48]Christian Albrechts University, Kiel, Germany. [49]Institute of Human Genetics, Helmholtz Center Munich, Neuherberg, Germany. [50]Department of General Practice and Primary Health Care, University of Helsinki and Helsinki University Hospital, Helsinki Finland. [51]National Institute for Health and Welfare, Helsinki, Finland. [52]Institute of Clinical Medicine, University of Helsinki, Helsinki, Finland. [53]Department of Environmental Health, Harvard T.H. Chan School of Public Health, Boston, USA 02115. [54]Dept of Internal Medicine, Erasmus University Medical Center, Rotterdam, the Netherlands, 3015 CN. [55]Dept of Pain Management and Research, Oslo University Hospital, Oslo, 0424 Oslo, Norway. [56]Medical Faculty, University of Oslo, Oslo, 0318 Oslo, Norway. [57]Division of Mental Health, Norwegian Institute of Public Health,P.O. Box 4404 Nydalen, Oslo, Norway, NO-0403. [58]Kiel Pain and Headache Center, 24149 Kiel, Germany. [59]Danish Headache Center, Department of Neurology, Rigshospitalet, Glostrup Hospital, University of Copenhagen, Denmark. [60]Institute of Biological Psychiatry, Mental Health Center Sct. Hans, University of Copenhagen, Roskilde, Denmark.

[61]Institute Of Biological Psychiatry, MHC Sct. Hans, Mental Health Services Copenhagen, DK-2100 Copenhagen, Denmark. [62]Institute of Clinical Sciences, Faculty of Medicine and Health Sciences, University of Copenhagen, DK-2100 Copenhagen, Denmark. [63]iPSYCH - The Lundbeck Foundation's Initiative for Integrative Psychiatric Research, DK-2100 Copenhagen, Denmark. [64]A list of members and affiliations appears in the Supplementary Note. [65]Department of Public Health, University of Helsinki, Helsinki, Finland. [66]Department of Health, National Institute for Health and Welfare, Helsinki, Finland. [67]Research Centre of Applied and Preventive Cardiovascular Medicine, University of Turku, Turku, Finland, 20521. [68]Department of Clinical Physiology and Nuclear Medicine, Turku University Hospital, Turku, Finland, 20521. [69]Dept of Neurology, Erasmus University Medical Center, Rotterdam, the Netherlands, 3015 CN. [70]Imperial College London, Department of Epidemiology and Biostatistics, MRC Health Protection Agency (HPE) Centre for Environment and Health, School of Public Health, UK, W2 1PG. [71]University of Oulu, Biocenter Oulu, Finland, Box 5000, Fin-90014 University of Oulu. [72]Oulu University Hospital, Unit of Primary Care, Oulu, Finland, Box 10, Fin-90029 OYS. [73]University Medical Center Hamburg Eppendorf, Institute of Human Genetics, 20246 Hamburg, Germany. [74]Population Health Research Institute, St George's, University of London, Cranmer Terrace, London SW17 0RE, UK. [75]Munich Cluster for Systems Neurology (SyNergy), Munich, Germany. [76]Leiden University Medical Centre, Department of Human Genetics, Leiden, The Netherlands, PO Box 9600, 2300 RC. [77]Faculty of Medicine, University of Iceland, 101 Reykjavik, Iceland. [78]Statistical and Genomic Epidemiology Laboratory, Institute of Health and Biomedical Innovation, Queensland University of Technology, 60 Musk Ave, Kelvin Grove, QLD 4059, Australia. [79]Department of Neurology, Massachusetts General Hospital, Boston, USA.

* These authors contributed equally to this work. [§] These authors jointly supervised this work.

Author list for MEGASTROKE Consortium on paper "Multiancestry genome-wide association study of 520,000 subjects identifies 32 loci associated with stroke and stroke subtypes", Nature Genetics, 2018, 50: 524-537. doi:10.1038/s41588-018-0058-3 [PubMed ID: 29531354].

MEGASTROKE CONSORTIUM

Rainer Malik [1], Ganesh Chauhan [2], Matthew Traylor [3], Muralidharan Sargurupremraj [4,5], Yukinori Okada [6,7,8], Aniket Mishra [4,5], Loes Rutten-Jacobs [3], Anne-Katrin Giese [9], Sander W van der Laan [10], Solveig Gretarsdottir [11], Christopher D Anderson [12,13,14,14], Michael Chong [15], Hieab HH Adams [16,17], Tetsuro Ago [18], Peter Almgren [19], Philippe Amouyel [20,21], Hakan Ay [22,13], Traci M Bartz [23], Oscar R Benavente [24], Steve Bevan [25], Giorgio B Boncoraglio [26], Robert D Brown, Jr. [27], Adam S Butterworth [28,29], Caty Carrera [30,31], Cara L Carty [32,33], Daniel I Chasman [34,35], Wei-Min Chen [36], John W Cole [37], Adolfo Correa [38], Ioana Cotlarciuc [39], Carlos Cruchaga [40,41], John Danesh [28,42,43,44], Paul IW de Bakker [45,46], Anita L DeStefano [47,48], Marcel den Hoed [49], Qing Duan [50], Stefan T Engelter [51,52], Guido J Falcone [53,54], Rebecca F Gottesman [55], Raji P Grewal [56], Vilmundur Gudnason [57,58], Stefan Gustafsson [59], Jeffrey Haessler [60], Tamara B Harris [61], Ahamad Hassan [62], Aki S Havulinna [63,64], Susan R Heckbert [65], Elizabeth G Holliday [66,67], George Howard [68], Fang-Chi Hsu [69], Hyacinth I Hyacinth [70], M Arfan Ikram [16], Erik Ingelsson [71,72], Marguerite R Irvin [73], Xueqiu Jian [74], Jordi Jiménez-Conde [75], Julie A Johnson [76,77], J Wouter Jukema [78], Masahiro Kanai [6,7,79], Keith L Keene [80,81], Brett M Kissela [82], Dawn O Kleindorfer [82], Charles Kooperberg [60], Michiaki Kubo [83], Leslie A Lange [84], Carl D Langefeld [85], Claudia Langenberg [86], Lenore J Launer [87], Jin-Moo Lee [88], Robin Lemmens [89,90], Didier Leys [91], Cathryn M Lewis [92,93], Wei-Yu Lin [28,94], Arne G Lindgren [95,96], Erik Lorentzen [97], Patrik K Magnusson [98], Jane Maguire [99], Ani Manichaikul [36], Patrick F McArdle [100], James F Meschia [101], Braxton D Mitchell [100,102], Thomas H Mosley [103,104], Michael A Nalls [105,106], Toshiharu Ninomiya [107], Martin J O'Donnell [15,108], Bruce M Psaty [109,110,111,112], Sara L Pulit [113,45], Kristiina Rannikmäe [114,115], Alexander P Reiner [65,116], Kathryn M Rexrode [117], Kenneth Rice [118], Stephen S Rich [36], Paul M Ridker [34,35], Natalia S Rost [9,13], Peter M Rothwell [119], Jerome I Rotter [120,121], Tatjana Rundek [122], Ralph L Sacco [122], Saori Sakaue [7,123], Michele M Sale [124], Veikko Salomaa [63], Bishwa R Sapkota [125], Reinhold Schmidt [126], Carsten O Schmidt [127], Ulf Schminke [128], Pankaj Sharma [39], Agnieszka Slowik [129], Cathie LM Sudlow [114,115], Christian Tanislav [130], Turgut Tatlisumak [131,132], Kent D Taylor [120,121], Vincent NS Thijs [133,134], Gudmar Thorleifsson [11], Unnur Thorsteinsdottir [11], Steffen Tiedt [1], Stella Trompet [135], Christophe Tzourio [5,136,137], Cornelia M van Duijn [138,139], Matthew Walters [140], Nicholas J Wareham [86], Sylvia Wassertheil-Smoller [141], James G Wilson [142], Kerri L Wiggins [109], Qiong Yang [47], Salim Yusuf [15], Najaf Amin [16], Hugo S Aparicio [185,48], Donna K Arnett [186], John Attia [187], Alexa S Beiser [47,48], Claudine Berr [188], Julie E Buring [34,35], Mariana Bustamante [189], Valeria Caso [190], Yu-Ching Cheng [191], Seung Hoan Choi [192,48], Ayesha Chowhan [185,48], Natalia Cullell [31], Jean-François Dartigues [193,194], Hossein Delavaran [95,96], Pilar Delgado [195], Marcus Dörr [196,197], Gunnar Engström [19], Ian Ford [198], Wander S Gurpreet [199], Anders Hamsten [200,201], Laura Heitsch [202], Atsushi Hozawa [203], Laura Ibanez [204], Andreea Ilinca [95,96], Martin Ingelsson [205], Motoki Iwasaki [206], Rebecca D Jackson [207], Katarina Jood [208], Pekka Jousilahti [63], Sara Kaffashian [4,5], Lalit Kalra [209], Masahiro Kamouchi [210], Takanari Kitazono [211], Olafur Kjartansson [212], Manja Kloss [213], Peter J Koudstaal [214], Jerzy Krupinski [215], Daniel L Labovitz [216], Cathy C Laurie [118], Christopher R Levi [217], Linxin Li [218], Lars Lind [219], Cecilia M Lindgren [220,221], Vasileios Lioutas [222,48], Yong Mei Liu [223], Oscar L Lopez [224], Hirata Makoto [225], Nicolas Martinez-Majander [172], Koichi Matsuda [225], Naoko Minegishi [203], Joan Montaner [226], Andrew P Morris [227,228], Elena Muiño [31], Martina Müller-Nurasyid [229,230,231], Bo Norrving [95,96], Soichi Ogishima [203], Eugenio A Parati [232], Leema Reddy Peddareddygari [56], Nancy L Pedersen [98,233], Joanna Pera [129], Markus Perola [63,234], Alessandro Pezzini [235], Silvana Pileggi [236], Raquel Rabionet [237], Iolanda Riba-Llena [30], Marta Ribasés [238], Jose R Romero [185,48], Jaume Roquer [239,240], Anthony G Rudd [241,242], Antti-Pekka Sarin [243,244], Ralhan Sarju [199], Chloe Sarnowski [47,48], Makoto Sasaki [245], Claudia L Satizabal [185,48], Mamoru Satoh [245], Naveed Sattar [246], Norie Sawada [206], Gerli Sibolt [172], Ásgeir Sigurdsson [247], Albert Smith [248], Kenji Sobue [245], Carolina Soriano-Tárraga [240], Tara Stanne [249], O Colin Stine [250], David J Stott [251], Konstantin Strauch [229,252], Takako Takai [203], Hideo Tanaka [253,254], Kozo Tanno [245], Alexander Teumer [255], Liisa Tomppo [172], Nuria P Torres-Aguila [31], Emmanuel Touze [256,257], Shoichiro Tsugane [206], Andre G Uitterlinden [258], Einar M Valdimarsson [259], Sven J van der Lee [16], Henry Völzke [255], Kenji Wakai [253], David Weir [260], Stephen R Williams [261], Charles DA Wolfe [241,242], Quenna Wong [118], Huichun Xu [191], Taiki Yamaji [206], Dharambir K Sanghera [125,169,170], Olle Melander [19], Christina Jern [171], Daniel Strbian [172,173], Israel Fernandez-Cadenas [31,30], W T Longstreth, Jr [174,65], Arndt Rolfs [175], Jun Hata [107], Daniel Woo [82], Jonathan Rosand [12,13,14], Guillaume Pare [15], Jemma C Hopewell [176], Danish Saleheen [177], Kari Stefansson [11,178], Bradford B Worrall [179], Steven J Kittner [37], Sudha Seshadri [180,48], Myriam Fornage [74,181], Hugh S Markus [3], Joanna MM Howson [28], Yoichiro Kamatani [6,182], Stephanie Debette [4,5], Martin Dichgans [1,183,184]

1 Institute for Stroke and Dementia Research (ISD), University Hospital, LMU Munich, Munich, Germany

2 Centre for Brain Research, Indian Institute of Science, Bangalore, India
3 Stroke Research Group, Division of Clinical Neurosciences, University of Cambridge, UK
4 INSERM U1219 Bordeaux Population Health Research Center, Bordeaux, France
5 University of Bordeaux, Bordeaux, France
6 Laboratory for Statistical Analysis, RIKEN Center for Integrative Medical Sciences, Yokohama, Japan
7 Department of Statistical Genetics, Osaka University Graduate School of Medicine, Osaka, Japan
8 Laboratory of Statistical Immunology, Immunology Frontier Research Center (WPI-IFReC), Osaka University, Suita, Japan.
9 Department of Neurology, Massachusetts General Hospital, Harvard Medical School, Boston, MA, USA
10 Laboratory of Experimental Cardiology, Division of Heart and Lungs, University Medical Center Utrecht, University of Utrecht, Utrecht,Netherlands
11 deCODE genetics/AMGEN inc, Reykjavik, Iceland
12 Center for Genomic Medicine, Massachusetts General Hospital (MGH), Boston, MA, USA
13 J. Philip Kistler Stroke Research Center, Department of Neurology, MGH, Boston, MA, USA
14 Program in Medical and Population Genetics, Broad Institute, Cambridge, MA, USA
15 Population Health Research Institute, McMaster University, Hamilton, Canada
16 Department of Epidemiology, Erasmus University Medical Center, Rotterdam, Netherlands
17 Department of Radiology and Nuclear Medicine, Erasmus University Medical Center, Rotterdam, Netherlands
18 Department of Medicine and Clinical Science, Graduate School of Medical Sciences, Kyushu University, Fukuoka, Japan
19 Department of Clinical Sciences, Lund University, Malmö, Sweden
20 Univ. Lille, Inserm, Institut Pasteur de Lille, LabEx DISTALZ-UMR1167, Risk factors and molecular determinants of aging-related diseases, F-59000 Lille, France
21 Centre Hosp. Univ Lille, Epidemiology and Public Health Department, F-59000 Lille, France
22 AA Martinos Center for Biomedical Imaging, Department of Radiology, Massachusetts General Hospital, Harvard Medical School, Boston, MA, USA
23 Cardiovascular Health Research Unit, Departments of Biostatistics and Medicine, University of Washington, Seattle, WA, USA
24 Division of Neurology, Faculty of Medicine, Brain Research Center, University of British Columbia, Vancouver, Canada
25 School of Life Science, University of Lincoln, Lincoln, UK
26 Department of Cerebrovascular Diseases, Fondazione IRCCS Istituto Neurologico "Carlo Besta", Milano, Italy
27 Department of Neurology, Mayo Clinic Rochester, Rochester, MN, USA
28 MRC/BHF Cardiovascular Epidemiology Unit, Department of Public Health and Primary Care, University of Cambridge, Cambridge, UK
29 The National Institute for Health Research Blood and Transplant Research Unit in Donor Health and Genomics, University of Cambridge, UK
30 Neurovascular Research Laboratory, Vall d'Hebron Institut of Research, Neurology and Medicine Departments-Universitat Autònoma de Barcelona, Vall d'Hebrón Hospital, Barcelona, Spain
31 Stroke Pharmacogenomics and Genetics, Fundacio Docència i Recerca MutuaTerrassa, Terrassa, Spain
32 Children's Research Institute, Children's National Medical Center, Washington, DC, USA
33 Center for Translational Science, George Washington University, Washington, DC, USA
34 Division of Preventive Medicine, Brigham and Women's Hospital, Boston, MA, USA
35 Harvard Medical School, Boston, MA, USA
36 Center for Public Health Genomics, Department of Public Health Sciences, University of Virginia, Charlottesville, VA, USA
37 Department of Neurology, University of Maryland School of Medicine and Baltimore VAMC, Baltimore, MD, USA
38 Departments of Medicine, Pediatrics and Population Health Science, University of Mississippi Medical Center, Jackson, MS, USA
39 Institute of Cardiovascular Research, Royal Holloway University of London, UK & Ashford and St Peters Hospital, Surrey UK
40 Department of Psychiatry,The Hope Center Program on Protein Aggregation and Neurodegeneration (HPAN),Washington University, School of Medicine, St. Louis, MO, USA
41 Department of Developmental Biology, Washington University School of Medicine, St. Louis, MO, USA

42 NIHR Blood and Transplant Research Unit in Donor Health and Genomics, Department of Public Health and Primary Care, University of Cambridge, Cambridge, UK
43 Wellcome Trust Sanger Institute, Wellcome Trust Genome Campus, Hinxton, Cambridge, UK
44 British Heart Foundation, Cambridge Centre of Excellence, Department of Medicine, University of Cambridge, Cambridge, UK
45 Department of Medical Genetics, University Medical Center Utrecht, Utrecht, Netherlands
46 Department of Epidemiology, Julius Center for Health Sciences and Primary Care, University Medical Center Utrecht, Utrecht, Netherlands
47 Boston University School of Public Health, Boston, MA, USA
48 Framingham Heart Study, Framingham, MA, USA
49 Department of Immunology, Genetics and Pathology and Science for Life Laboratory, Uppsala University, Uppsala, Sweden
50 Department of Genetics, University of North Carolina, Chapel Hill, NC, USA
51 Department of Neurology and Stroke Center, Basel University Hospital, Switzerland
52 Neurorehabilitation Unit, University and University Center for Medicine of Aging and Rehabilitation Basel, Felix Platter Hospital, Basel, Switzerland
53 Department of Neurology, Yale University School of Medicine, New Haven, CT, USA
54 Program in Medical and Population Genetics, The Broad Institute of Harvard and MIT, Cambridge, MA, USA
55 Department of Neurology, Johns Hopkins University School of Medicine, Baltimore, MD, USA
56 Neuroscience Institute, SF Medical Center, Trenton, NJ, USA
57 Icelandic Heart Association Research Institute, Kopavogur, Iceland
58 University of Iceland, Faculty of Medicine, Reykjavik, Iceland
59 Department of Medical Sciences, Molecular Epidemiology and Science for Life Laboratory, Uppsala University, Uppsala, Sweden
60 Division of Public Health Sciences, Fred Hutchinson Cancer Research Center, Seattle, WA, USA
61 Laboratory of Epidemiology and Population Science, National Institute on Aging, National Institutes of Health, Bethesda, MD, USA
62 Department of Neurology, Leeds General Infirmary, Leeds Teaching Hospitals NHS Trust, Leeds, UK
63 National Institute for Health and Welfare, Helsinki, Finland
64 FIMM - Institute for Molecular Medicine Finland, Helsinki, Finland
65 Department of Epidemiology, University of Washington, Seattle, WA, USA
66 Public Health Stream, Hunter Medical Research Institute, New Lambton, Australia
67 Faculty of Health and Medicine, University of Newcastle, Newcastle, Australia
68 School of Public Health, University of Alabama at Birmingham, Birmingham, AL, USA
69 Department of Biostatistical Sciences, Wake Forest School of Medicine, Winston-Salem, NC, USA
70 Aflac Cancer and Blood Disorder Center, Department of Pediatrics, Emory University School of Medicine, Atlanta, GA, USA
71 Department of Medicine, Division of Cardiovascular Medicine, Stanford University School of Medicine, CA, USA
72 Department of Medical Sciences, Molecular Epidemiology and Science for Life Laboratory, Uppsala University, Uppsala, Sweden
73 Epidemiology, School of Public Health, University of Alabama at Birmingham, USA
74 Brown Foundation Institute of Molecular Medicine, University of Texas Health Science Center at Houston, Houston, TX, USA
75 Neurovascular Research Group (NEUVAS), Neurology Department, Institut Hospital del Mar d'Investigació Mèdica, Universitat Autònoma de Barcelona, Barcelona, Spain
76 Department of Pharmacotherapy and Translational Research and Center for Pharmacogenomics, University of Florida, College of Pharmacy, Gainesville, FL, USA
77 Division of Cardiovascular Medicine, College of Medicine, University of Florida, Gainesville, FL, USA
78 Department of Cardiology, Leiden University Medical Center, Leiden, the Netherlands
79 Program in Bioinformatics and Integrative Genomics, Harvard Medical School, Boston, MA, USA
80 Department of Biology, East Carolina University, Greenville, NC, USA
81 Center for Health Disparities, East Carolina University, Greenville, NC, USA
82 University of Cincinnati College of Medicine, Cincinnati, OH, USA
83 RIKEN Center for Integrative Medical Sciences, Yokohama, Japan

84 Department of Medicine, University of Colorado Denver, Anschutz Medical Campus, Aurora, CO, USA
85 Center for Public Health Genomics and Department of Biostatistical Sciences, Wake Forest School of Medicine, Winston-Salem, NC, USA
86 MRC Epidemiology Unit, University of Cambridge School of Clinical Medicine, Institute of Metabolic Science, Cambridge Biomedical Campus, Cambridge, UK
87 Intramural Research Program, National Institute on Aging, National Institutes of Health, Bethesda, MD, USA
88 Department of Neurology, Radiology, and Biomedical Engineering, Washington University School of Medicine, St. Louis, MO, USA
89 KU Leuven – University of Leuven, Department of Neurosciences, Experimental Neurology, Leuven, Belgium
90 VIB Center for Brain & Disease Research, University Hospitals Leuven, Department of Neurology, Leuven, Belgium
91 Univ.-Lille, INSERM U 1171. CHU Lille. Lille, France
92 Department of Medical and Molecular Genetics, King's College London, London, UK
93 SGDP Centre, Institute of Psychiatry, Psychology & Neuroscience, King's College London, London, UK
94 Northern Institute for Cancer Research, Paul O'Gorman Building, Newcastle University, Newcastle, UK
95 Department of Clinical Sciences Lund, Neurology, Lund University, Lund, Sweden
96 Department of Neurology and Rehabilitation Medicine, Skåne University Hospital, Lund, Sweden
97 Bioinformatics Core Facility, University of Gothenburg, Gothenburg, Sweden
98 Department of Medical Epidemiology and Biostatistics, Karolinska Institutet, Stockholm, Sweden
99 University of Technology Sydney, Faculty of Health, Ultimo, Australia
100 Department of Medicine, University of Maryland School of Medicine, MD, USA
101 Department of Neurology, Mayo Clinic, Jacksonville, FL, USA
102 Geriatrics Research and Education Clinical Center, Baltimore Veterans Administration Medical Center, Baltimore, MD, USA
103 Division of Geriatrics, School of Medicine, University of Mississippi Medical Center, Jackson, MS, USA
104 Memory Impairment and Neurodegenerative Dementia Center, University of Mississippi Medical Center, Jackson, MS, USA
105 Laboratory of Neurogenetics, National Institute on Aging, National institutes of Health, Bethesda, MD, USA
106 Data Tecnica International, Glen Echo MD, USA
107 Department of Epidemiology and Public Health, Graduate School of Medical Sciences, Kyushu University, Fukuoka, Japan
108 Clinical Research Facility, Department of Medicine, NUI Galway, Galway, Ireland
109 Cardiovascular Health Research Unit, Department of Medicine, University of Washington, Seattle, WA, USA
110 Department of Epidemiology, University of Washington, Seattle, WA
111 Department of Health Services, University of Washington, Seattle, WA, USA
112 Kaiser Permanente Washington Health Research Institute, Seattle, WA, USA
113 Brain Center Rudolf Magnus, Department of Neurology, University Medical Center Utrecht, Utrecht, The Netherlands
114 Usher Institute of Population Health Sciences and Informatics, University of Edinburgh, Edinburgh, UK
115 Centre for Clinical Brain Sciences, University of Edinburgh, Edinburgh, UK
116 Fred Hutchinson Cancer Research Center, University of Washington, Seattle, WA, USA
117 Department of Medicine, Brigham and Women's Hospital, Boston, MA, USA
118 Department of Biostatistics, University of Washington, Seattle, WA, USA
119 Nuffield Department of Clinical Neurosciences, University of Oxford, UK
120 Institute for Translational Genomics and Population Sciences, Los Angeles Biomedical Research Institute at Harbor-UCLA Medical Center, Torrance, CA, USA
121 Division of Genomic Outcomes, Department of Pediatrics, Harbor-UCLA Medical Center, Torrance, CA, USA
122 Department of Neurology, Miller School of Medicine, University of Miami, Miami, FL, USA
123 Department of Allergy and Rheumatology, Graduate School of Medicine, the University of Tokyo, Tokyo, Japan
124 Center for Public Health Genomics, University of Virginia, Charlottesville, VA, USA
125 Department of Pediatrics, College of Medicine, University of Oklahoma Health Sciences Center, Oklahoma City, OK, USA
126 Department of Neurology, Medical University of Graz, Graz, Austria
127 University Medicine Greifswald, Institute for Community Medicine, SHIP-KEF, Greifswald, Germany
128 University Medicine Greifswald, Department of Neurology, Greifswald, Germany
129 Department of Neurology, Jagiellonian University, Krakow, Poland

130 Department of Neurology, Justus Liebig University, Giessen, Germany
131 Department of Clinical Neurosciences/Neurology, Institute of Neuroscience and Physiology, Sahlgrenska Academy at University of Gothenburg, Gothenburg, Sweden
132 Sahlgrenska University Hospital, Gothenburg, Sweden
133 Stroke Division, Florey Institute of Neuroscience and Mental Health, University of Melbourne, Heidelberg, Australia
134 Austin Health, Department of Neurology, Heidelberg, Australia
135 Department of Internal Medicine, Section Gerontology and Geriatrics, Leiden University Medical Center, Leiden, the Netherlands
136 INSERM U1219, Bordeaux, France
137 Department of Public Health, Bordeaux University Hospital, Bordeaux, France
138 Genetic Epidemiology Unit, Department of Epidemiology, Erasmus University Medical Center Rotterdam, Netherlands
139 Center for Medical Systems Biology, Leiden, Netherlands
140 School of Medicine, Dentistry and Nursing at the University of Glasgow, Glasgow, UK
141 Department of Epidemiology and Population Health, Albert Einstein College of Medicine, NY, USA
142 Department of Physiology and Biophysics, University of Mississippi Medical Center, Jackson, MS, USA
143 A full list of members and affiliations appears in the Supplementary Note
144 Department of Human Genetics, McGill University, Montreal, Canada
145 Department of Pathophysiology, Institute of Biomedicine and Translation Medicine, University of Tartu, Tartu, Estonia
146 Department of Cardiac Surgery, Tartu University Hospital, Tartu, Estonia
147 Clinical Gene Networks AB,Stockholm, Sweden
148 Department of Genetics and Genomic Sciences, The Icahn Institute for Genomics and Multiscale Biology Icahn School of Medicine at Mount Sinai, New York, NY , USA
149 Department of Pathophysiology, Institute of Biomedicine and Translation Medicine, University of Tartu, Biomeedikum, Tartu, Estonia
150 Integrated Cardio Metabolic Centre, Department of Medicine, Karolinska Institutet, Karolinska Universitetssjukhuset, Huddinge, Sweden.
151 Clinical Gene Networks AB, Stockholm, Sweden
152 Sorbonne Universités, UPMC Univ. Paris 06, INSERM, UMR_S 1166, Team Genomics & Pathophysiology of Cardiovascular Diseases, Paris, France
153 ICAN Institute for Cardiometabolism and Nutrition, Paris, France
154 Department of Biomedical Engineering, University of Virginia, Charlottesville, VA, USA
155 Group Health Research Institute, Group Health Cooperative, Seattle, WA, USA
156 Seattle Epidemiologic Research and Information Center, VA Office of Research and Development, Seattle, WA, USA
157 Cardiovascular Research Center, Massachusetts General Hospital, Boston, MA, USA
158 Department of Medical Research, Bærum Hospital, Vestre Viken Hospital Trust, Gjettum, Norway
159 Saw Swee Hock School of Public Health, National University of Singapore and National University Health System, Singapore
160 National Heart and Lung Institute, Imperial College London, London, UK
161 Department of Gene Diagnostics and Therapeutics, Research Institute, National Center for Global Health and Medicine, Tokyo, Japan
162 Department of Epidemiology, Tulane University School of Public Health and Tropical Medicine, New Orleans, LA, USA
163 Department of Cardiology,University Medical Center Groningen, University of Groningen, Netherlands
164 MRC-PHE Centre for Environment and Health, School of Public Health, Department of Epidemiology and Biostatistics, Imperial College London, London, UK
165 Department of Epidemiology and Biostatistics, Imperial College London, London, UK
166 Department of Cardiology, Ealing Hospital NHS Trust, Southall, UK
167 National Heart, Lung and Blood Research Institute, Division of Intramural Research, Population Sciences Branch, Framingham, MA, USA
168 A full list of members and affiliations appears at the end of the manuscript
169 Department of Phamaceutical Sciences, Collge of Pharmacy, University of Oklahoma Health Sciences Center, Oklahoma City, OK, USA
170 Oklahoma Center for Neuroscience, Oklahoma City, OK, USA

171 Department of Pathology and Genetics, Institute of Biomedicine, The Sahlgrenska Academy at University of Gothenburg, Gothenburg, Sweden
172 Department of Neurology, Helsinki University Hospital, Helsinki, Finland
173 Clinical Neurosciences, Neurology, University of Helsinki, Helsinki, Finland
174 Department of Neurology, University of Washington, Seattle, WA, USA
175 Albrecht Kossel Institute, University Clinic of Rostock, Rostock, Germany
176 Clinical Trial Service Unit and Epidemiological Studies Unit, Nuffield Department of Population Health, University of Oxford, Oxford, UK
177 Department of Genetics, Perelman School of Medicine, University of Pennsylvania, PA, USA
178 Faculty of Medicine, University of Iceland, Reykjavik, Iceland
179 Departments of Neurology and Public Health Sciences, University of Virginia School of Medicine, Charlottesville, VA, USA
180 Department of Neurology, Boston University School of Medicine, Boston, MA, USA
181 Human Genetics Center, University of Texas Health Science Center at Houston, Houston, TX, USA
182 Center for Genomic Medicine, Kyoto University Graduate School of Medicine, Kyoto, Japan
183 Munich Cluster for Systems Neurology (SyNergy), Munich, Germany
184 German Center for Neurodegenerative Diseases (DZNE), Munich, Germany
185 Boston University School of Medicine, Boston, MA, USA
186 University of Kentucky College of Public Health, Lexington, KY, USA
187 University of Newcastle and Hunter Medical Research Institute, New Lambton, Australia
188 Univ. Montpellier, Inserm, U1061, Montpellier, France
189 Centre for Research in Environmental Epidemiology, Barcelona, Spain
190 Department of Neurology, Università degli Studi di Perugia, Umbria, Italy
191 Department of Medicine, University of Maryland School of Medicine, Baltimore, MD, USA
192 Broad Institute, Cambridge, MA, USA
193 Univ. Bordeaux, Inserm, Bordeaux Population Health Research Center, UMR 1219, Bordeaux, France
194 Bordeaux University Hospital, Department of Neurology, Memory Clinic, Bordeaux, France
195 Neurovascular Research Laboratory. Vall d'Hebron Institut of Research, Neurology and Medicine Departments-Universitat Autònoma de Barcelona. Vall d'Hebrón Hospital, Barcelona, Spain
196 University Medicine Greifswald, Department of Internal Medicine B, Greifswald, Germany
197 DZHK, Greifswald, Germany
198 Robertson Center for Biostatistics, University of Glasgow, Glasgow, UK
199 Hero DMC Heart Institute, Dayanand Medical College & Hospital, Ludhiana, India
200 Atherosclerosis Research Unit, Department of Medicine Solna, Karolinska Institutet, Stockholm, Sweden
201 Karolinska Institutet, Stockholm, Sweden
202 Division of Emergency Medicine, and Department of Neurology, Washington University School of Medicine, St. Louis, MO, USA
203 Tohoku Medical Megabank Organization, Sendai, Japan
204 Department of Psychiatry, Washington University School of Medicine, St. Louis, MO, USA
205 Department of Public Health and Caring Sciences / Geriatrics, Uppsala University, Uppsala, Sweden
206 Epidemiology and Prevention Group, Center for Public Health Sciences, National Cancer Center, Tokyo, Japan
207 Department of Internal Medicine and the Center for Clinical and Translational Science, The Ohio State University, Columbus, OH, USA
208 Institute of Neuroscience and Physiology, the Sahlgrenska Academy at University of Gothenburg, Goteborg, Sweden
209 Department of Basic and Clinical Neurosciences, King's College London, London, UK
210 Department of Health Care Administration and Management, Graduate School of Medical Sciences, Kyushu University, Japan
211 Department of Medicine and Clinical Science, Graduate School of Medical Sciences, Kyushu University, Japan
212 Landspitali National University Hospital, Departments of Neurology & Radiology, Reykjavik, Iceland
213 Department of Neurology, Heidelberg University Hospital, Germany
214 Department of Neurology, Erasmus University Medical Center
215 Hospital Universitari Mutua Terrassa, Terrassa (Barcelona), Spain
216 Albert Einstein College of Medicine, Montefiore Medical Center, New York, NY, USA
217 John Hunter Hospital, Hunter Medical Research Institute and University of Newcastle, Newcastle, NSW, Australia

218 Centre for Prevention of Stroke and Dementia, Nuffield Department of Clinical Neurosciences, University of Oxford, UK
219 Department of Medical Sciences, Uppsala University, Uppsala, Sweden
220 Genetic and Genomic Epidemiology Unit, Wellcome Trust Centre for Human Genetics, University of Oxford, Oxford, UK
221 The Wellcome Trust Centre for Human Genetics, Oxford, UK
222 Beth Israel Deaconess Medical Center, Boston, MA, USA
223 Wake Forest School of Medicine, Wake Forest, NC, USA
224 Department of Neurology, University of Pittsburgh, Pittsburgh, PA, USA
225 BioBank Japan, Laboratory of Clinical Sequencing, Department of Computational biology and medical Sciences, Graduate school of Frontier Sciences, The University of Tokyo, Tokyo, Japan
226 Neurovascular Research Laboratory, Vall d'Hebron Institut of Research, Neurology and Medicine Departments-Universitat Autònoma de Barcelona. Vall d'Hebrón Hospital, Barcelona, Spain
227 Department of Biostatistics, University of Liverpool, Liverpool, UK
228 Wellcome Trust Centre for Human Genetics, University of Oxford, Oxford, UK
229 Institute of Genetic Epidemiology, Helmholtz Zentrum München - German Research Center for Environmental Health, Neuherberg, Germany
230 Department of Medicine I, Ludwig-Maximilians-Universität, Munich, Germany
231 DZHK (German Centre for Cardiovascular Research), partner site Munich Heart Alliance, Munich, Germany
232 Department of Cerebrovascular Diseases, Fondazione IRCCS Istituto Neurologico "Carlo Besta", Milano, Italy
233 Karolinska Institutet, MEB, Stockholm, Sweden
234 University of Tartu, Estonian Genome Center, Tartu, Estonia, Tartu, Estonia
235 Department of Clinical and Experimental Sciences, Neurology Clinic, University of Brescia, Italy
236 Translational Genomics Unit, Department of Oncology, IRCCS Istituto di Ricerche Farmacologiche Mario Negri, Milano, Italy
237 Department of Genetics, Microbiology and Statistics, University of Barcelona, Barcelona, Spain
238 Psychiatric Genetics Unit, Group of Psychiatry, Mental Health and Addictions, Vall d'Hebron Research Institute (VHIR), Universitat Autònoma de Barcelona, Biomedical Network Research Centre on Mental Health (CIBERSAM), Barcelona, Spain
239 Department of Neurology, IMIM-Hospital del Mar, and Universitat Autònoma de Barcelona, Spain
240 IMIM (Hospital del Mar Medical Research Institute), Barcelona, Spain
241 National Institute for Health Research Comprehensive Biomedical Research Centre, Guy's & St. Thomas' NHS Foundation Trust and King's College London, London, UK
242 Division of Health and Social Care Research, King's College London, London, UK
243 FIMM-Institute for Molecular Medicine Finland, Helsinki, Finland
244 THL-National Institute for Health and Welfare, Helsinki, Finland
245 Iwate Tohoku Medical Megabank Organization, Iwate Medical University, Iwate, Japan
246 BHF Glasgow Cardiovascular Research Centre, Faculty of Medicine, Glasgow, UK
247 deCODE Genetics/Amgen, Inc., Reykjavik, Iceland
248 Icelandic Heart Association, Reykjavik, Iceland
249 Institute of Biomedicine, the Sahlgrenska Academy at University of Gothenburg, Goteborg, Sweden
250 Department of Epidemiology, University of Maryland School of Medicine, Baltimore, MD, USA
251 Institute of Cardiovascular and Medical Sciences, Faculty of Medicine, University of Glasgow, Glasgow, UK
252 Chair of Genetic Epidemiology, IBE, Faculty of Medicine, LMU Munich, Germany
253 Division of Epidemiology and Prevention, Aichi Cancer Center Research Institute, Nagoya, Japan
254 Department of Epidemiology, Nagoya University Graduate School of Medicine, Nagoya, Japan
255 University Medicine Greifswald, Institute for Community Medicine, SHIP-KEF, Greifswald, Germany
256 Department of Neurology, Caen University Hospital, Caen, France
257 University of Caen Normandy, Caen, France
258 Department of Internal Medicine, Erasmus University Medical Center, Rotterdam, Netherlands
259 Landspitali University Hospital, Reykjavik, Iceland
260 Survey Research Center, University of Michigan, Ann Arbor, MI, USA
261 University of Virginia Department of Neurology, Charlottesville, VA, USA

Author list for Coronary ARtery DIsease Genome Wide Replication and Meta-analysis (CARDIoGRAM) on paper: **Association analyses based on false discovery rate implicate new loci for coronary artery disease. Nat Genet** 49**, 1385-1391 (2017)**

Christopher P Nelson [1,2,60], Anuj Goel [3,4,60] , Adam S Butterworth [5,6,60], Stavroula Kanoni [7,8,60] , Tom R Webb [1,2], Eirini Marouli [7,8], Lingyao Zeng [9], Ioanna Ntalla [7,8], Florence Y Lai [1,2] , Jemma C Hopewell [10], Olga Giannakopoulou [7,8], Tao Jiang [5], Stephen E Hamby [1,2] , Emanuele Di Angelantonio [5,6], Themistocles L Assimes [11], Erwin P Bottinger [12], John C Chambers [13–15] , Robert Clarke [10], Colin N A Palmer [16,17] , Richard M Cubbon [18], Patrick Ellinor [19] , Raili Ermel[20] , Evangelos Evangelou [13,21] , Paul W Franks [22–24], Christopher Grace [3,4], Dongfeng Gu [25], Aroon D Hingorani [26], Joanna M M Howson [5], Erik Ingelsson [27], Adnan Kastrati [9], Thorsten Kessler [9], Theodosios Kyriakou [3,4], Terho Lehtimäki [28], Xiangfeng Lu [27], Yingchang Lu [12,29], Winfried März [30–32], Ruth McPherson [33], Andres Metspalu [34], Mar Pujades-Rodriguez[35], Arno Ruusalepp [20,36], Eric E Schadt[37], Amand F Schmidt[38], Michael J Sweeting[5], Pierre A Zalloua [39,40], Kamal Al Ghalayini[41], Bernard D Keavney [42,43] , Jaspal S Kooner [14,15,44], Ruth J F Loos [12,45], Riyaz S Patel [46,47], Martin K Rutter [48,49], Maciej Tomaszewski [43,50], Ioanna Tzoulaki [13,21], Eleftheria Zeggini [51] , Jeanette Erdmann [52–54], George Dedoussis [55], Johan L M Björkegren [36,37,56], EPICCVD Consortium [57], CARDIoGRAMplusC4D [57], The UK Biobank CardioMetabolic Consortium CHD working group [57], Heribert Schunkert [9,60], Martin Farrall [3,4,60], John Danesh [5,6,58,60], Nilesh J Samani [1,2,60], Hugh Watkins [3,4,60] & Panos Deloukas [7,8,59,60]

Affiliations at the time the manuscript was published:

1Department of Cardiovascular Sciences, University of Leicester, Leicester, UK. 2National Institute for Health Research Leicester Biomedical Research Centre, Leicester, UK. 3Division of Cardiovascular Medicine, Radcliffe Department of Medicine, University of Oxford, Oxford, UK. 4Wellcome Trust Centre for Human Genetics, University of Oxford, Oxford, UK. 5MRC/BHF Cardiovascular Epidemiology Unit, Department of Public Health and Primary Care, University of Cambridge, Cambridge, UK. 6NIHR Blood and Transplant Research Unit in Donor Health and Genomics, Department of Public Health and Primary Care, University of Cambridge, Cambridge, UK. 7William Harvey Research Institute, Barts & the London Medical School, Queen Mary University of London, London, UK. 8Centre for Genomic Health, Queen Mary University of London, London, UK. 9German Heart Center Munich, Clinic at Technische Universität München and Deutsches Zentrum für Herz- und Kreislauferkrankungen (DZHK), partner site Munich Heart Alliance, Munich, Germany. 10CTSU, Nuffield Department of Population Health, University of Oxford, Oxford, UK. 11Department of Medicine, Stanford University School of Medicine, Stanford, California, USA. 12Charles Bronfman Institute for Personalized Medicine, Icahn School of Medicine at Mount Sinai, New York, New York, USA. 13Department of Epidemiology and Biostatistics, Imperial College London, London, UK. 14Department of Cardiology, Ealing Hospital, London North West Healthcare NHS Trust, Southall, UK. 15Imperial College Healthcare NHS Trust, London, UK. 16Molecular and Clinical Medicine, Biomedical Research Institute, University of Dundee, Ninewells Hospital, Dundee, UK. 17 Pharmacogenomics Centre, Biomedical Research Institute, University of Dundee, Ninewells Hospital, Dundee, UK. 18 Leeds Institute of Cardiovascular and Metabolic Medicine, University of Leeds, Leeds, UK. 19Cardiac Arrhythmia Service and Cardiovascular Research Center, Broad Institute of Harvard and Massachusetts Institute of Technology, Boston, Massachusetts, USA. 20Department of Cardiac Surgery, Tartu University Hospital, Tartu, Nature Genetics: doi:10.1038/ng.3913 Estonia. 21Department of Hygiene and Epidemiology, University of Ioannina Medical School, Ioannina, Greece. 22Department of Clinical Sciences, Genetic & Molecular Epidemiology Unit, Lund University Diabetes Center, Skåne University Hospital, Lund University, Malmö, Sweden. 23Department of Nutrition, Harvard T.H. Chan School of Public Health, Harvard University, Boston, Massachusetts, USA. 24Department of Public Health and Clinical Medicine, Unit of Medicine, Umeå University, Umeå, Sweden. 25State Key Laboratory of Cardiovascular Disease, Fuwai Hospital, National Center of Cardiovascular Diseases, Chinese Academy of Medical Sciences and Peking Union Medical College, Beijing, China. 26University College London, London, UK. 27Department of Medicine, Division of Cardiovascular Medicine, Stanford University School of Medicine, Stanford, California, USA. 28Department of Clinical Chemistry, Fimlab Laboratories and Faculty of Medicine and Life Sciences, University of Tampere, Tampere, Finland. 29Division of Epidemiology, Department of Medicine, Vanderbilt-Ingram Cancer Center, Vanderbilt Epidemiology Center, Vanderbilt University School of Medicine, Nashville, Tennessee, USA. 30Clinical Institute of Medical and Chemical Laboratory Diagnostics, Medical University of Graz, Graz, Austria. 31Medical Clinic V (Nephrology, Rheumatology, Hypertensiology, Endocrinology, Diabetology), Medical Faculty Mannheim, University of Heidelberg, Mannheim, Germany. 32Academy, Synlab Holding Deutschland GmbH, Mannheim, Germany. 33Ruddy Canadian Cardiovascular Genetics Centre, University of Ottawa Heart Institute, Ottawa, Ontario, Canada. 34Estonian Genome Center, University of Tartu, Tartu, Estonia. 35Leeds Institute of Biomedical and Clinical Sciences, University of Leeds, Leeds, UK. 36Clinical Gene Networks AB, Stockholm, Sweden. 37Department of Genetics and Genomic Sciences, Icahn Institute for Genomics and Multiscale Biology, Icahn School of Medicine at Mount Sinai, New York, New York, USA. 38Institute of Cardiovascular Science, UCL, London, UK. 39 Lebanese American University, School of Medicine, Beirut, Lebanon. 40Harvard T.H. Chan School of Public Health, Boston, Massachusetts, USA. 41Department of Medicine, King Abdulaziz University, Jeddah, Saudi Arabia. 42Division of Cardiovascular Sciences, Faculty of Biology, Medicine and Health, University of Manchester, Manchester, UK. 43Central Manchester University Hospitals NHS Foundation Trust, Manchester Academic Health Science Centre, Manchester, UK. 44Cardiovascular Science, National Heart and Lung Institute, Imperial College London, London, UK. 45Mindich Child Health Development Institute, Icahn School of Medicine at Mount Sinai, New York, New York, USA. 46Farr Institute of Health Informatics, UCL, London, UK. 47Bart's Heart Centre, St Bartholomew's Hospital, London, UK. 48Division of Diabetes, Endocrinology and Gastroenterology, Faculty of Biology, Medicine and Health, University of Manchester, Manchester, UK. 49Manchester Diabetes Centre, Central Manchester University Hospitals NHS Foundation Trust, Manchester Academic Health Science Centre, Manchester, UK. 50Division of Medicine, Central Manchester NHS Foundation Trust, Manchester Academic Health Science Centre, Manchester, UK. 51Wellcome Trust Sanger Institute, Hinxton, UK. 52Institute for Cardiogenetics, University of Lübeck, Lübeck, Germany. 53DZHK (German Research Centre for Cardiovascular Research), partner site Hamburg/Lübeck/Kiel, Lübeck, Germany. 54University Heart Center Lübeck, Lübeck, Germany. 55Department of Nutrition-Dietetics, Harokopio University, Athens, Greece. 56 Integrated Cardio Metabolic Centre, Department of Medicine, Karolinska Institutet,

Karolinska Universitetssjukhuset, Huddinge, Sweden. 57A list of members and affiliations appears in the Supplementary Note. 58Wellcome Trust Sanger Institute, Wellcome Trust Genome Campus, Hinxton, UK. 59Princess Al-Jawhara Al-Brahim Centre of Excellence in Research of Hereditary Disorders (PACER-HD), Nature Genetics: doi:10.1038/ng.3913 King Abdulaziz University, Jeddah, Saudi Arabia. 60These authors contributed equally to this work.

Author list for International Consortium of Blood Pressure-Genome Wide Association Studies (ICBP)on paper: Genetic analysis of over 1 million people identifies 535 new loci associated with blood pressure traits. Nat Genet 50**, 1412-1425 (2018).**

Evangelos Evangelou 1 2, Helen R Warren 3 4, David Mosen-Ansorena 1, Borbala Mifsud 3, Raha Pazoki 1, He Gao 1 5, Georgios Ntritsos 2, Niki Dimou 2, Claudia P Cabrera 3 4, Ibrahim Karaman 1, Fu Liang Ng 3, Marina Evangelou 1 6, Katarzyna Witkowska 3, Evan Tzanis 3, Jacklyn N Hellwege 7, Ayush Giri 8, Digna R Velez Edwards 8, Yan V Sun 9 10, Kelly Cho 11 12, J Michael Gaziano 11 12, Peter W F Wilson 13, Philip S Tsao 14, Csaba P Kovesdy 15, Tonu Esko 16 17, Reedik Mägi 16, Lili Milani 16, Peter Almgren 18, Thibaud Boutin 19, Stéphanie Debette 20 21, Jun Ding 22, Franco Giulianini 23, Elizabeth G Holliday 24, Anne U Jackson 25, Ruifang Li-Gao 26, Wei-Yu Lin 27, Jian'an Luan 28, Massimo Mangino 29 30, Christopher Oldmeadow 24, Bram Peter Prins 31, Yong Qian 22, Muralidharan Sargurupremraj 21, Nabi Shah 32 33, Praveen Surendran 27, Sébastien Thériault 34 35, Niek Verweij 17 36 37, Sara M Willems 28, Jing-Hua Zhao 28, Philippe Amouyel 38, John Connell 39, Renée de Mutsert 26, Alex S F Doney 32, Martin Farrall 40 41, Cristina Menni 29, Andrew D Morris 42, Raymond Noordam 43, Guillaume Paré 34, Neil R Poulter 44, Denis C Shields 45, Alice Stanton 46, Simon Thom 47, Gonçalo Abecasis 48, Najaf Amin 49, Dan E Arking 50, Kristin L Ayers 51 52, Caterina M Barbieri 53, Chiara Batini 54, Joshua C Bis 55, Tineka Blake 54, Murielle Bochud 56, Michael Boehnke 25, Eric Boerwinkle 57, Dorret I Boomsma 58, Erwin P Bottinger 59, Peter S Braund 60 61, Marco Brumat 62, Archie Campbell 63 64, Harry Campbell 65, Aravinda Chakravarti 50, John C Chambers 1 5 66 67 68, Ganesh Chauhan 69, Marina Ciullo 70 71, Massimiliano Cocca 72, Francis Collins 73, Heather J Cordell 51, Gail Davies 74 75, Martin H de Borst 76, Eco J de Geus 58, Ian J Deary 74 75, Joris Deelen 77, Fabiola Del Greco M 78, Cumhur Yusuf Demirkale 79, Marcus Dörr 80 81, Georg B Ehret 50 82, Roberto Elosua 83 84, Stefan Enroth 85, A Mesut Erzurumluoglu 54, Teresa Ferreira 86 87, Mattias Frånberg 88 89 90, Oscar H Franco 91, Ilaria Gandin 62, Paolo Gasparini 62 72, Vilmantas Giedraitis 92, Christian Gieger 93 94 95, Giorgia Girotto 62 72, Anuj Goel 40 41, Alan J Gow 74 96, Vilmundur Gudnason 97 98, Xiuqing Guo 99, Ulf Gyllensten 85, Anders Hamsten 88 89, Tamara B Harris 100, Sarah E Harris 63 74, Catharina A Hartman 101, Aki S Havulinna 102 103, Andrew A Hicks 78, Edith Hofer 104 105, Albert Hofman 91 106, Jouke-Jan Hottenga 58, Jennifer E Huffman 19 107 108, Shih-Jen Hwang 107 108, Erik Ingelsson 109 110, Alan James 111 112, Rick Jansen 113, Marjo-Riitta Jarvelin 1 5 114 115 116, Roby Joehanes 107 117, Åsa Johansson 85, Andrew D Johnson 107 118, Peter K Joshi 65, Pekka Jousilahti 102, J Wouter Jukema 119, Antti Jula 102, Mika Kähönen 120 121, Sekar Kathiresan 17 36 122, Bernard D Keavney 123 124, Kay-Tee Khaw 125, Paul Knekt 102, Joanne Knight 126, Ivana Kolcic 127, Jaspal S Kooner 5 67 68 128, Seppo Koskinen 102, Kati Kristiansson 102, Zoltan Kutalik 56 129, Maris Laan 130, Marty Larson 107, Lenore J Launer 100, Benjamin Lehne 1, Terho Lehtimäki 131 132, David C M Liewald 74 75, Li Lin 82, Lars Lind 133, Cecilia M Lindgren 40 87 134, YongMei Liu 135, Ruth J F Loos 28 59 136, Lorna M Lopez 74 137 138, Yingchang Lu 59, Leo-Pekka Lyytikäinen 131 132, Anubha Mahajan 40, Chrysovalanto Mamasoula 139, Jaume Marrugat 83, Jonathan Marten 19, Yuri Milaneschi 140, Anna Morgan 62, Andrew P Morris 40 141, Alanna C Morrison 142, Peter J Munson 79, Mike A Nalls 143 144, Priyanka Nandakumar 50, Christopher P Nelson 60 61, Teemu Niiranen 102 145, Ilja M Nolte 146, Teresa Nutile 70, Albertine J Oldehinkel 147, Ben A Oostra 49, Paul F O'Reilly 148, Elin Org 16, Sandosh Padmanabhan 64 149, Walter Palmas 150, Aarno Palotie 103 151 152, Alison Pattie 75, Brenda W J H Penninx 140, Markus Perola 102 103 153, Annette Peters 94 95 154, Ozren Polasek 127 155, Peter P Pramstaller 78 156 157, Quang Tri Nguyen 79, Olli T Raitakari 158 159, Meixia Ren 160, Rainer Rettig 161, Kenneth Rice 162, Paul M Ridker 23 163, Janina S Ried 94, Harriëtte Riese 147, Samuli Ripatti 103 164, Antonietta Robino 72, Lynda M Rose 23, Jerome I Rotter 99, Igor Rudan 65, Daniela Ruggiero 70 71, Yasaman Saba 165, Cinzia F Sala 53, Veikko Salomaa 102, Nilesh J Samani 60 61, Antti-Pekka Sarin 103, Reinhold Schmidt 104, Helena Schmidt 165, Nick Shrine 54, David Siscovick 166, Albert V Smith 97 98, Harold Snieder 146, Siim Sõber 130, Rossella Sorice 70, John M Starr 74 167, David J Stott 168, David P Strachan 169, Rona J Strawbridge 88 89, Johan Sundström 133, Morris A Swertz 170, Kent D Taylor 99, Alexander Teumer 81 171, Martin D Tobin 54, Maciej Tomaszewski 123 124, Daniela Toniolo 53, Michela Traglia 53, Stella Trompet 119 172, Jaakko Tuomilehto 173 174 175 176, Christophe Tzourio 21, André G Uitterlinden 91 177, Ahmad Vaez 146 178, Peter J van der Most 146, Cornelia M van Duijn 49, Anne-Claire Vergnaud 1, Germaine C Verwoert 91, Veronique Vitart 19, Uwe Völker 81 179, Peter Vollenweider 180, Dragana Vuckovic 62 181, Hugh Watkins 40 41, Sarah H Wild 182, Gonneke Willemsen 58, James F Wilson 19 65, Alan F Wright 19, Jie Yao 99, Tatijana Zemunik 183, Weihua Zhang 1 67, John R Attia 24, Adam S Butterworth 27 184, Daniel I Chasman 23 163, David Conen 185 186, Francesco Cucca 187 188, John Danesh 27 184, Caroline Hayward 19, Joanna M M Howson 27, Markku Laakso 189, Edward G Lakatta 190, Claudia Langenberg 28, Olle Melander 18, Dennis O Mook-Kanamori 26 191, Colin N A Palmer 32, Lorenz Risch 192 193 194, Robert A Scott 28, Rodney J Scott 24, Peter Sever 128, Tim D Spector 29, Pim van der Harst 195, Nicholas J Wareham 28, Eleftheria Zeggini 31, Daniel Levy 107 118, Patricia B Munroe 3 4, Christopher Newton-Cheh 134 196 197, Morris J Brown 3 4, Andres Metspalu 16, Adriana M Hung 198, Christopher J O'Donnell 199, Todd L Edwards 7, Bruce M Psaty 200 201, Ioanna Tzoulaki 1 2 5, Michael R Barnes 3 4, Louise V Wain 54 61, Paul Elliott 202 203 204 205 206, Mark J Caulfield 207 208, Million Veteran Program

Affiliations at the time the manuscript was published:

1Department of Epidemiology and Biostatistics, Imperial College London, London, UK.
2Department of Hygiene and Epidemiology, University of Ioannina Medical School, Ioannina, Greece.
3William Harvey Research Institute, Barts and The London School of Medicine and Dentistry, Queen Mary University of London, London, UK.
4National Institute for Health Research, Barts Cardiovascular Biomedical Research Center, Queen Mary University of London, London, UK.
5MRC-PHE Centre for Environment and Health, Imperial College London, London, UK.
6Department of Mathematics, Imperial College London, London, UK.
7Division of Epidemiology, Department of Medicine, Institute for Medicine and Public Health, Vanderbilt Genetics Institute, Vanderbilt University Medical Center, Tennessee Valley Healthcare System (626)/Vanderbilt University, Nashville, TN, USA.
8Vanderbilt Genetics Institute, Vanderbilt Epidemiology Center, Department of Obstetrics and Gynecology, Vanderbilt University Medical Center; Tennessee Valley Health Systems VA, Nashville, TN, USA.
9Department of Epidemiology, Emory University Rollins School of Public Health, Atlanta, GA, USA.

10Department of Biomedical Informatics, Emory University School of Medicine, Atlanta, GA, USA.
11Massachusetts Veterans Epidemiology Research and Information Center (MAVERIC), VA Boston Healthcare System, Boston, MA, USA.
12Division of Aging, Department of Medicine, Brigham and Women's Hospital; Department of Medicine, Harvard Medical School, Boston, MA, USA.
13Atlanta VAMC and Emory Clinical Cardiovascular Research Institute, Atlanta, GA, USA.
14VA Palo Alto Health Care System, Division of Cardiovascular Medicine, Stanford University School of Medicine, Stanford, CA, USA.
15Nephrology Section, Memphis VA Medical Center and University of Tennessee Health Science Center, Memphis, TN, USA.
16Estonian Genome Center, University of Tartu, Tartu, Estonia.
17Program in Medical and Population Genetics, Broad Institute of Harvard and MIT, Cambridge, MA, USA.
18Department Clinical Sciences, Malmö, Lund University, Malmö, Sweden.
19MRC Human Genetics Unit, MRC Institute of Genetics and Molecular Medicine, University of Edinburgh, Western General Hospital, Edinburgh, Scotland, UK.
20Department of Neurology, Bordeaux University Hospital, Bordeaux, France.
21Univ. Bordeaux, Inserm, Bordeaux Population Health Research Center, CHU Bordeaux, Bordeaux, France.
22Laboratory of Genetics and Genomics, NIA/NIH, Baltimore, MD, USA.
23Division of Preventive Medicine, Brigham and Women's Hospital, Boston, MA, USA.
24Hunter Medical Research Institute and Faculty of Health, University of Newcastle, New Lambton Heights, New South Wales, Australia.
25Department of Biostatistics and Center for Statistical Genetics, University of Michigan, Ann Arbor, MI, USA.
26Department of Clinical Epidemiology, Leiden University Medical Center, Leiden, the Netherlands.
27MRC/BHF Cardiovascular Epidemiology Unit, Department of Public Health and Primary Care, University of Cambridge, Cambridge, UK.
28MRC Epidemiology Unit, University of Cambridge School of Clinical Medicine, Cambridge, UK.
29Department of Twin Research and Genetic Epidemiology, Kings College London, London, UK.
30NIHR Biomedical Research Centre at Guy's and St Thomas' Foundation Trust, London, UK.
31Wellcome Trust Sanger Institute, Hinxton, UK.
32Division of Molecular and Clinical Medicine, School of Medicine, University of Dundee, Dundee, UK.
33Department of Pharmacy, COMSATS Institute of Information Technology, Abbottabad, Pakistan.
34Department of Pathology and Molecular Medicine, McMaster University, Hamilton, Ontario, Canada.
35Institut universitaire de cardiologie et de pneumologie de Québec-Université Laval, Quebec City, Quebec, Canada.
36Cardiovascular Research Center and Center for Human Genetic Research, Massachusetts General Hospital, Boston, MA, USA.
37University of Groningen, University Medical Center Groningen, Department of Cardiology, Groningen, the Netherlands.
38University of Lille, Inserm, Centre Hosp. Univ. Lille, Institut Pasteur de Lille, UMR1167 - RID-AGE - Risk factors and molecular determinants of aging-related diseases, Epidemiology and Public Health Department, Lille, France.
39University of Dundee, Ninewells Hospital & Medical School, Dundee, UK.
40Wellcome Trust Centre for Human Genetics, University of Oxford, Oxford, UK.
41Division of Cardiovascular Medicine, Radcliffe Department of Medicine, University of Oxford, Oxford, UK.
42Usher Institute of Population Health Sciences and Informatics, University of Edinburgh, Edinburgh, UK.
43Department of Internal Medicine, Section Gerontology and Geriatrics, Leiden University Medical Center, Leiden, the Netherlands.
44Imperial Clinical Trials Unit, London, UK.
45School of Medicine, University College Dublin, Dublin, Ireland.
46Molecular and Cellular Therapeutics, Royal College of Surgeons in Ireland, Dublin, Ireland.
47International Centre for Circulatory Health, Imperial College London, London, UK.
48Center for Statistical Genetics, Department of Biostatistics, SPH II, Washington Heights, Ann Arbor, MI, USA.
49Genetic Epidemiology Unit, Department of Epidemiology, Erasmus MC, Rotterdam, the Netherlands.
50Center for Complex Disease Genomics, McKusick-Nathans Institute of Genetic Medicine, Johns Hopkins University School of Medicine, Baltimore, MD, USA.
51Institute of Genetic Medicine, Newcastle University, Newcastle upon Tyne, UK.
52Sema4, a Mount Sinai venture, Stamford, CT, USA.

53Division of Genetics and Cell Biology, San Raffaele Scientific Institute, Milan, Italy.
54Department of Health Sciences, University of Leicester, Leicester, UK.
55Cardiovascular Health Research Unit, Department of Medicine, University of Washington, Seattle, WA, USA.
56Institute of Social and Preventive Medicine, University Hospital of Lausanne, Lausanne, Switzerland.
57Human Genetics Center, School of Public Health, The University of Texas Health Science Center at Houston and Human Genome Sequencing Center, Baylor College of Medicine, Houston, TX, USA.
58Department of Biological Psychology, Vrije Universiteit Amsterdam, EMGO+ Institute, VU University Medical Center, Amsterdam, the Netherlands.
59The Charles Bronfman Institute for Personalized Medicine, Icahn School of Medicine at Mount Sinai, New York, NY, USA.
60Department of Cardiovascular Sciences, University of Leicester, Leicester, UK.
61NIHR Leicester Biomedical Research Centre, Glenfield Hospital, Leicester, UK.
62Department of Medical, Surgical and Health Sciences, University of Trieste, Trieste, Italy.
63Medical Genetics Section, Centre for Genomic and Experimental Medicine, Institute of Genetics and Molecular Medicine, University of Edinburgh, Edinburgh, UK.
64Generation Scotland, Centre for Genomic and Experimental Medicine, University of Edinburgh, Edinburgh, UK.
65Centre for Global Health Research, Usher Institute of Population Health Sciences and Informatics, University of Edinburgh, Edinburgh, Scotland, UK.
66Lee Kong Chian School of Medicine, Nanyang Technological University, Singapore, Singapore.
67Department of Cardiology, Ealing Hospital, Middlesex, UK.
68Imperial College Healthcare NHS Trust, London, UK.
69Centre for Brain Research, Indian Institute of Science, Bangalore, India.
70Institute of Genetics and Biophysics "A. Buzzati-Traverso", CNR, Napoli, Italy.
71IRCCS Neuromed, Pozzilli, Isernia, Italy.
72Institute for Maternal and Child Health IRCCS Burlo Garofolo, Trieste, Italy.
73Medical Genomics and Metabolic Genetics Branch, National Human Genome Research Institute, NIH, Bethesda, MD, USA.
74Centre for Cognitive Ageing and Cognitive Epidemiology, University of Edinburgh, Edinburgh, UK.
75Department of Psychology, University of Edinburgh, Edinburgh, UK.
76Department of Internal Medicine, Division of Nephrology, University of Groningen, University Medical Center Groningen, Groningen, the Netherlands.
77Department of Molecular Epidemiology, Leiden University Medical Center, Leiden, the Netherlands.
78Institute for Biomedicine, Eurac Research, Bolzano, Italy - Affiliated Institute of the University of Lübeck, Lübeck, Germany.
79Mathematical and Statistical Computing Laboratory, Office of Intramural Research, Center for Information Technology, National Institutes of Health, Bethesda, MD, USA.
80Department of Internal Medicine B, University Medicine Greifswald, Greifswald, Germany.
81DZHK (German Centre for Cardiovascular Research), partner site Greifswald, Greifswald, Germany.
82Cardiology, Department of Medicine, Geneva University Hospital, Geneva, Switzerland.
83CIBERCV & Cardiovascular Epidemiology and Genetics, IMIM, Barcelona, Spain.
84Faculty of Medicine, Universitat de Vic-Central de Catalunya, Vic, Spain.
85Department of Immunology, Genetics and Pathology, Uppsala Universitet, Science for Life Laboratory, Uppsala, Sweden.
86Wellcome Centre for Human Genetics, University of Oxford, Oxford, UK.
87Big Data Institute, Li Ka Shing Center for Health for Health Information and Discovery, Oxford University, Oxford, UK.
88Cardiovascular Medicine Unit, Department of Medicine Solna, Karolinska Institutet, Stockholm, Sweden.
89Centre for Molecular Medicine, L8:03, Karolinska Universitetsjukhuset, Solna, Sweden.
90Department of Numerical Analysis and Computer Science, Stockholm University, Stockholm, Sweden.
91Department of Epidemiology, Erasmus MC, Rotterdam, the Netherlands.
92Department of Public Health and Caring Sciences, Geriatrics, Uppsala, Sweden.
93Research Unit of Molecular Epidemiology, Helmholtz Zentrum München, German Research Center for Environmental Health, Neuherberg, Germany.

94Institute of Epidemiology, Helmholtz Zentrum München, German Research Center for Environmental Health, Neuherberg, Germany.
95German Center for Diabetes Research (DZD e.V.), Neuherberg, Germany.
96Department of Psychology, School of Social Sciences, Heriot-Watt University, Edinburgh, UK.
97Faculty of Medicine, University of Iceland, Reykjavik, Iceland.
98Icelandic Heart Association, Kopavogur, Iceland.
99The Institute for Translational Genomics and Population Sciences, Department of Pediatrics, LABioMed at Harbor-UCLA Medical Center, Torrance, CA, USA.
100Intramural Research Program, Laboratory of Epidemiology, Demography, and Biometry, National Institute on Aging, Bethesda, MD, USA.
101Department of Psychiatry, University of Groningen, University Medical Center Groningen, Groningen, the Netherlands.
102Department of Public Health Solutions, National Institute for Health and Welfare (THL), Helsinki, Finland.
103Institute for Molecular Medicine Finland (FIMM), University of Helsinki, Helsinki, Finland.
104Clinical Division of Neurogeriatrics, Department of Neurology, Medical University of Graz, Graz, Austria.
105Institute for Medical Informatics, Statistics and Documentation, Medical University of Graz, Graz, Austria.
106Department of Epidemiology, Harvard T.H. Chan School of Public Health, Boston, MA, USA.
107National Heart, Lung and Blood Institute's Framingham Heart Study, Framingham, MA, USA.
108The Population Science Branch, Division of Intramural Research, National Heart, Lung, and Blood Institute, National Institutes of Health, Bethesda, MD, USA.
109Department of Medical Sciences, Molecular Epidemiology and Science for Life Laboratory, Uppsala University, Uppsala, Sweden.
110Division of Cardiovascular Medicine, Department of Medicine, Stanford University School of Medicine, Stanford, CA, USA.
111Department of Pulmonary Physiology and Sleep, Sir Charles Gairdner Hospital, Hospital Avenue, Nedlands, Western Australia, Australia.
112School of Medicine and Pharmacology, University of Western Australia, Perth, Western Australia, Australia.
113Department of Psychiatry, VU University Medical Center, Amsterdam Neuroscience, Amsterdam, the Netherlands.
114Biocenter Oulu, University of Oulu, Oulu, Finland.
115Center For Life-course Health Research, University of Oulu, Oulu, Finland.
116Unit of Primary Care, Oulu University Hospital, Oulu, Oulu, Finland.
117Hebrew SeniorLife, Harvard Medical School, Boston, MA, USA.
118Population Sciences Branch, National Heart, Lung, and Blood Institute, National Institutes of Health, Bethesda, MD, USA.
119Department of Cardiology, Leiden University Medical Center, Leiden, the Netherlands.
120Department of Clinical Physiology, Tampere University Hospital, Tampere, Finland.
121Department of Clinical Physiology, Finnish Cardiovascular Research Center - Tampere, Faculty of Medicine and Life Sciences, University of Tampere, Tampere, Finland.
122Broad Institute of the Massachusetts Institute of Technology and Harvard University, Cambridge, MA, USA.
123Division of Cardiovascular Sciences, Faculty of Biology, Medicine and Health, The University of Manchester, Manchester, UK.
124Division of Medicine, Manchester University NHS Foundation Trust, Manchester Academic Health Science Centre, Manchester, UK.
125Department of Public Health and Primary Care, Institute of Public Health, University of Cambridge, Cambridge, UK.
126Data Science Institute and Lancaster Medical School, Lancaster, UK.
127Department of Public Health, Faculty of Medicine, University of Split, Split, Croatia.
128National Heart and Lung Institute, Imperial College London, London, UK.
129Swiss Institute of Bioinformatics, Lausanne, Switzerland.
130Institute of Biomedicine and Translational Medicine, University of Tartu, Tartu, Estonia.
131Department of Clinical Chemistry, Fimlab Laboratories, Tampere, Finland.
132Department of Clinical Chemistry, Finnish Cardiovascular Research Center - Tampere, Faculty of Medicine and Life Sciences, University of Tampere, Tampere, Finland.
133Department of Medical Sciences, Cardiovascular Epidemiology, Uppsala University, Uppsala, Sweden.
134Program in Medical and Population Genetics, Broad Institute, Cambridge, MA, USA.
135Division of Public Health Sciences, Wake Forest School of Medicine, Winston-Salem, NC, USA.

136Mindich Child Health Development Institute, The Icahn School of Medicine at Mount Sinai, New York, NY, USA.
137Department of Psychiatry, Royal College of Surgeons in Ireland, Education and Research Centre, Beaumont Hospital, Dublin, Ireland.
138University College Dublin, UCD Conway Institute, Centre for Proteome Research, UCD, Belfield, Dublin, Ireland.
139Institute of Health and Society, Newcastle University, Newcastle upon Tyne, UK.
140Department of Psychiatry, Amsterdam Public Health and Amsterdam Neuroscience, VU University Medical Center/GGZ inGeest, Amsterdam, the Netherlands.
141Department of Biostatistics, University of Liverpool, Liverpool, UK.
142Department of Epidemiology, Human Genetics and Environmental Sciences, School of Public Health, University of Texas Health Science Center at Houston, Houston, TX, USA.
143Data Tecnica International, Glen Echo, MD, USA.
144Laboratory of Neurogenetics, National Institute on Aging, Bethesda, MD, USA.
145Department of Medicine, Turku University Hospital and University of Turku, Turku, Finland.
146Department of Epidemiology, University of Groningen, University Medical Center Groningen, Groningen, the Netherlands.
147Interdisciplinary Center Psychopathology and Emotion Regulation (ICPE), University of Groningen, University Medical Center Groningen, Groningen, the Netherlands.
148SGDP Centre, Institute of Psychiatry, Psychology and Neuroscience, King's College London, London, UK.
149British Heart Foundation Glasgow Cardiovascular Research Centre, Institute of Cardiovascular and Medical Sciences, College of Medical, Veterinary and Life Sciences, University of Glasgow, Glasgow, UK.
150Department of Medicine, Columbia University Medical Center, New York, NY, USA.
151Analytic and Translational Genetics Unit, Department of Medicine, Department of Neurology and Department of Psychiatry, Massachusetts General Hospital, Boston, MA, USA.
152The Stanley Center for Psychiatric Research and Program in Medical and Population Genetics, The Broad Institute of MIT and Harvard, Cambridge, MA, USA.
153University of Tartu, Tartu, Estonia.
154German Center for Cardiovascular Disease Research (DZHK), partner site Munich, Neuherberg, Germany.
155Psychiatric Hospital "Sveti Ivan", Zagreb, Croatia.
156Department of Neurology, General Central Hospital, Bolzano, Italy.
157Department of Neurology, University of Lübeck, Lübeck, Germany.
158Department of Clinical Physiology and Nuclear Medicine, Turku University Hospital, Turku, Finland.
159Research Centre of Applied and Preventive Cardiovascular Medicine, University of Turku, Turku, Finland.
160Fujian Key Laboratory of Geriatrics, Department of Geriatric Medicine, Fujian Provincial Hospital, Fujian Medical University, Fuzhou, China.
161Institute of Physiology, University Medicine Greifswald, Karlsburg, Germany.
162Department of Biostatistics, University of Washington, Seattle, WA, USA.
163Harvard Medical School, Boston, MA, USA.
164Public Health, Faculty of Medicine, University of Helsinki, Helsinki, Finland.
165Gottfried Schatz Research Center for Cell Signaling, Metabolism & Aging, Molecular Biology and Biochemistry, Medical University of Graz, Graz, Austria.
166The New York Academy of Medicine, New York, NY, USA.
167Alzheimer Scotland Dementia Research Centre, University of Edinburgh, Edinburgh, UK.
168Institute of Cardiovascular and Medical Sciences, Faculty of Medicine, University of Glasgow, Glasgow, UK.
169Population Health Research Institute, St George's, University of London, London, UK.
170Department of Genetics, University of Groningen, University Medical Center Groningen, Groningen, the Netherlands.
171Institute for Community Medicine, University Medicine Greifswald, Greifswald, Germany.
172Department of Gerontology and Geriatrics, Leiden University Medical Center, Leiden, the Netherlands.
173Dasman Diabetes Institute, Dasman, Kuwait.
174Chronic Disease Prevention Unit, National Institute for Health and Welfare, Helsinki, Finland.
175Department of Public Health, University of Helsinki, Helsinki, Finland.
176Saudi Diabetes Research Group, King Abdulaziz University, Jeddah, Saudi Arabia.
177Department of Internal Medicine, Erasmus MC, Rotterdam, the Netherlands.
178Research Institute for Primordial Prevention of Non-communicable Disease, Isfahan University of Medical Sciences, Isfahan, Iran.

179Interfaculty Institute for Genetics and Functional Genomics, University Medicine Greifswald, Greifswald, Germany.
180Department of Internal Medicine, University Hospital, CHUV, Lausanne, Switzerland.
181Experimental Genetics Division, Sidra Medical and Research Center, Doha, Qatar.
182Centre for Population Health Sciences, Usher Institute of Population Health Sciences and Informatics, University of Edinburgh, Edinburgh, Scotland, UK.
183Department of Biology, Faculty of Medicine, University of Split, Split, Croatia.
184The National Institute for Health Research Blood and Transplant Research Unit in Donor Health and Genomics, University of Cambridge, Cambridge, UK.
185Division of Cardiology, University Hospital, Basel, Switzerland.
186Division of Cardiology, Department of Medicine, McMaster University, Hamilton, Ontario, Canada.
187Institute of Genetic and Biomedical Research, National Research Council (CNR), Monserrato, Cagliari, Italy.
188Department of Biomedical Sciences, University of Sassari, Sassari, Italy.
189Institute of Clinical Medicine, Internal Medicine, University of Eastern Finland and Kuopio University Hospital, Kuopio, Finland.
190Laboratory of Cardiovascular Science, National Institute on Aging, National Institutes of Health, Baltimore, MD, USA.
191Department of Public Health and Primary Care, Leiden University Medical Center, Leiden, the Netherlands.
192Labormedizinisches Zentrum Dr. Risch, Schaan, Liechtenstein.
193Private University of the Principality of Liechtenstein, Triesen, Liechtenstein.
194University Institute of Clinical Chemistry, Inselspital, Bern University Hospital, University of Bern, Bern, Switzerland.
195Department of Cardiology, University of Groningen, University Medical Center Groningen, Groningen, the Netherlands.
196Center for Genomic Medicine, Massachusetts General Hospital, Boston, MA, USA.
197Cardiovascular Research Center, Massachusetts General Hospital, Boston, MA, USA.
198Tennessee Valley Healthcare System (Nashville VA) & Vanderbilt University, Nashville, TN, USA.
199VA Boston Healthcare, Section of Cardiology and Department of Medicine, Brigham and Women's Hospital, Harvard Medical School, Boston, MA, USA.
200Cardiovascular Health Research Unit, Departments of Medicine, Epidemiology and Health Services, University of Washington, Seattle, WA, USA.
201Kaiser Permanente Washington Health Research Institute, Seattle, WA, USA.
202Department of Epidemiology and Biostatistics, Imperial College London, London, UK. p.elliott@imperial.ac.uk.
203MRC-PHE Centre for Environment and Health, Imperial College London, London, UK. p.elliott@imperial.ac.uk.
204National Institute for Health Research Imperial Biomedical Research Centre, Imperial College Healthcare NHS Trust and Imperial College London, London, UK. p.elliott@imperial.ac.uk.
205UK Dementia Research Institute (UK DRI) at Imperial College London, London, UK. p.elliott@imperial.ac.uk.
206Health Data Research-UK London substantive site, London, UK. p.elliott@imperial.ac.uk.
207William Harvey Research Institute, Barts and The London School of Medicine and Dentistry, Queen Mary University of London, London, UK. m.j.caulfield@qmul.ac.uk.
208National Institute for Health Research, Barts Cardiovascular Biomedical Research Center, Queen Mary University of London, London, UK. m.j.caulfield@qmul.ac.uk.

References

1. Mancuso N, Shi H, Goddard P, Kichaev G, Gusev A, Pasaniuc B. Integrating Gene Expression with Summary Association Statistics to Identify Genes Associated with 30 Complex Traits. *Am J Hum Genet* 2017; **100**(3): 473-87.
2. GTex Consortium. The Genotype-Tissue Expression (GTEx) project. *Nat Genet* 2013; **45**(6): 580-5.
3. Gusev A, Ko A, Shi H, et al. Integrative approaches for large-scale transcriptome-wide association studies. *Nat Genet* 2016; **48**(3): 245-52.
4. Gusev A, Mancuso N, Won H, et al. Transcriptome-wide association study of schizophrenia and chromatin activity yields mechanistic disease insights. *Nat Genet* 2018; **50**(4): 538-48.
5. Yang J, Ferreira T, Morris AP, et al. Conditional and joint multiple-SNP analysis of GWAS summary statistics identifies additional variants influencing complex traits. *Nat Genet* 2012; **44**(4): 369-75, S1-3.
6. Yavorska OO, Burgess S. MendelianRandomization: an R package for performing Mendelian randomization analyses using summarized data. *Int J Epidemiol* 2017; **46**(6): 1734-9.
7. Hemani G, Tilling K, Davey Smith G. Orienting the causal relationship between imprecisely measured traits using GWAS summary data. *PLoS Genet* 2017; **13**(11): e1007081.
