# Supplementary material for: A genome-wide cross-phenotype meta-analysis of the association of blood pressure with migraine
Source: Nat Commun. 2020 Jul 6;11:3368. doi: 10.1038/s41467-020-17002-0 (PMC7338361; doi:10.1038/s41467-020-17002-0)
Supplement: Supplementary file 2 — Peer Review File [file 41467_2020_17002_MOESM2_ESM.docx]

Peer Review Information

**Journal:** Nature Communications

**Manuscript title:** Genetic basis for blood pressure associations with migraine: a genome-wide cross-phenotype meta-analysis study

**Corresponding author name(s):** Dr Daniel Chasman

**Editorial notes:**

**Transferred manuscripts**

This manuscript has been previously reviewed at another journal that is not operating a transparent peer review scheme. This document only contains reviewer comments and rebuttal letters for versions considered at *Nature Communications*.

**Reviewer comments & decisions:**

**Reviewer comments, first version:**

Reviewer #1 (Remarks to the Author):

The manuscript by Guo et al provides interesting and important new information about shared genetic risks between blood pressure and migraine.

The manuscript is well written and uses state of the art methodology. I do not have much to comment, only one item that would benefit of clarification.

On line 114 the authors state that there were 39 000 hypertonia and migraine cases combined, overlapping samples. This overlap might result in some bias in the association analyses. It would be good to clarify how this overlap was handled. The authors should clarify this.


Reviewer #2 (Remarks to the Author):

This group around Daniel Chasman describes an epidemiolgic and genetic analysis on the relationship of blood pressure (BP) with migraine.
A very large sample size from several very large studies is used, including close to 60K cases of migrane. The authors find that DBP and (less so) SBP is associated with migrane. A few loci overlap. Additional loci are identified using a cross-trait meta-analysis. All SBP, DBP, and PP-genes are predictors of migraine with an OR of around 1.1-1.2 (inverse relationship for PP-genes). The DBP effect appears to explain the SBP effect on migraine, in conditional MR analyses.
The methods used for this work are clearly described, I have several comments below. The text is well written and the subject matter is interesting, of relevance for a general audience. I disagree with the conclusions.
MAJOR
1) The genetic correlation of the blood pressure and migraine phenotypes is weak at least. Only 1% (or less) of the variability of migraine genetics is explained by BP genetics. The authors could explain more on this. The differences between SBP and DBP are interesting, the authors do not mention which co-variates should be used and have been used in the parent studies. May there be residual confounding due to age?
2) The authors confound migraine vs. headache in the references of the introduction. Clinically, BP lowering is not used to avoid headaches; headache and hypertension: a myth (e.g. dx.doi.org/10.1136/jnnp.72.4.431), it should be mentioned that BP is higher when individuals have pain; but should hypertension and migraine occur at the same time, betablockers are a valid method of treating both. A few phrases in the introduction are contrary to these quite well established clinical findings. The phenotypes used need to be explained much more carefully as does the genotyping.
3) The cross-train meta-analysis is very difficult to interpret. What do these loci mean, given the very weak association between migraine and BP?
4) It is unclear how the p-values of the genetic-overlap results were derived and what is significant. The central figure is difficult to interpret in this sense.
5) The TWAS results are expressed as counts in Figure 2. The null distribution is not shown and it is unclear from the figure what is significantly different from the null.
6) The results contain many speculative comments on potential mechanisms that are unproven. The supplement is >100 pages and should be summarized much more.
7) Would it be appropriate to apply a multiple-testing correction to the MR-analyses?
MINOR
- Line 76: should state what type of "dissection"


Reviewer #3 (Remarks to the Author):

This report from Guo and Chasman explores the genetic relationships between blood pressure traits and migraine. This is an important question as it could help establish new treatment modalities for migraine treatment/prevention using blood pressure lowering medications. The analyses are thorough and methodologically sound. I do have some suggestions for the authors:

1) Have the authors tried analyzing MAP in addition to SBP, DBP and PP? This would nicely complement current analyses.

2) The gene-level genetic correlation is interesting as it appears some genes disproportionally contribute genetic correlation. Would results of the MR analysis hold if these genes were removed? This is relevant as bi-directional MR associations could potentially be explained by some loci affecting both traits (rather than one trait affecting the other and vice versa).

3) The opposing associations of SBP and DBP on migraine is intriguing. Could this explain the inconsistent epidemiological associations? If possible, attempting to do so would add to the insightfulness of the paper.

Guillaume Pare

**Author rebuttal, first version:**

Reviewer #1 (Remarks to the Author):

The manuscript by Guo et al provides interesting and important new information about shared genetic risks between blood pressure and migraine.

The manuscript is well written and uses state of the art methodology. I do not have much to comment, only one item that would benefit of clarification.

On line 114 the authors state that there were 39 000 hypertonia and migraine cases combined, overlapping samples. This overlap might result in some bias in the association analyses. It would be good to clarify how this overlap was handled. The authors should clarify this.

**Response:**

Thanks for the reviewer’s positive remarks about our manuscript.

We agree that the sample overlap might result in potential bias for the association analyses. However, firstly, the sample overlap (N=39,199) was relatively small comparing to the total sample size of migraine (N=375,752, proportion of overlap is ~10%) and BP (N=757,601, proportion of overlap is ~5%) summary statistics; secondly, LD score regression analysis controls for the sample overlap when the regression intercept is not constrained as in our analyses; thirdly, GNOVA also corrects for sample overlap and other potential sources of bias, and we report these corrected results; fourthly, the results from LD score regression and GNOVA are quite consistent. Therefore, in the current analysis, we believe the small proportion of sample overlap does not seriously affect the genetic correlation estimates. For transparency in the revised manuscript, we included the proportion of overlapping samples and the note the correction of sample overlap in LD Score regression and GNOVA in Methods on lines 454 and 475.

Reviewer #2 (Remarks to the Author):

This group around Daniel Chasman describes an epidemiologic and genetic analysis on the relationship of blood pressure (BP) with migraine.

A very large sample size from several very large studies is used, including close to 60K cases of migraine. The authors find that DBP and (less so) SBP is associated with migraine. A few loci overlap. Additional loci are identified using a cross-trait meta-analysis. All SBP, DBP, and PP-genes are predictors of migraine with an OR of around 1.1-1.2 (inverse relationship for PP-genes). The DBP effect appears to explain the SBP effect on migraine, in conditional MR analyses.

The methods used for this work are clearly described, I have several comments below. The text is well written and the subject matter is interesting, of relevance for a general audience. I disagree with the conclusions.

**Response:**

Regarding this last point, we hope that we have addressed each of the reviewer’s itemized criticisms so as to strengthen and clarify the presentation. In addition, we submit the following general comments about our approach and its intrinsic potential to support our conclusions. A key driver of our conclusions is the idea in MR that even if the genetic variants instrument have relatively small effects (e.g. on BP) but are nonetheless precise (i.e. relatively small standard errors, highly significant), the existence of consistent downstream effects on migraine across instruments may be taken as support for a causal mechanism. As described by developers of MR (see e.g. ref 21, 40, and 50), this framework derives from unique properties of genetic variants in epidemiology including the random allocation of alleles at conception and the irreversible nature of their inheritance. Indeed, the effects in this investigation are highly consistent across SNPs leading to significant MR estimates, and therefore strengthening our conclusions about an etiology of migraine closely related to BP, particularly DBP. Nevertheless, since the assumptions of MR cannot typically be confirmed unequivocally, findings in MR should always be interpreted as support or refutation of causal mechanism rather than proof. Accordingly, in the Discussion section, we are careful to qualify our conclusions.

We also show by genetic correlation analysis and cross-trait meta-analysis that particular loci appear to contribute to both BP and migraine susceptibility. These findings lend further support to the idea of shared vascular mechanisms related to BP and migraine, and the separate findings for DPB, SBP, and PP are further consistent with our conclusions from MR.

MAJOR

1) The genetic correlation of the blood pressure and migraine phenotypes is weak at least. Only 1% (or less) of the variability of migraine genetics is explained by BP genetics. The authors could explain more on this. The differences between SBP and DBP are interesting, the authors do not mention which co-variates should be used and have been used in the parent studies. May there be residual confounding due to age?

**Response:**

While we note that the largest genetic correlation is r_g_=0.11, *p*=3.56×10^-06^ between migraine and DBP (Table 1) rather than 1% as the reviewer states, we agree that the genetic correlations are relatively small compared to some other phenotypes, e.g. ~0.6 between psychiatric disorders and ~0.25 between lipids and coronary artery disease. However, the genetic correlations we observe are significant, and when we looked at the local genetic correlations, we observed strong effects at certain chromosomes, for example at chr22 (*r_g_*=0.47, *P*=1.37×10^-04^) between migraine and DBP and at chr19 (*r_g_*=-0.32, *P*=1.28×10^-03^) between migraine and PP. All these results suggest that BP and migraine have potential shared genetic etiologies worth investigating. In Results of the revised manuscript, we have added one sentence to further explain this (lines 234 to 239). We write: “Taken together, although the overall genetic correlations between BP traits and migraine were relatively modest compared to more closely related phenotypes, e.g. among psychiatric disorders (rg ~0.6) or between lipids and coronary artery disease (rg ~0.25), they nevertheless indicate potential shared genetic etiologies, especially at certain chromosomes or regions, and therefore worthy of additional investigation into potential mechanisms using cross-trait analysis and expression-trait analysis.”

Regarding the comparison of SBP and DBP, the summary level data for SBP and DBP in the current analysis were from the same study (and essentially the same cohorts), all analyses were adjusted for age, age[^2^](https://www.nature.com/articles/ng.3667#ref-CR2), sex, and body mass index (BMI) at the cohort level, and all cohorts corrected for hypertension treatment (+15/10 mm Hg in the presence of any hypertensive medication). Therefore, the differences between SBP and DBP are relatively unlikely due to potential differential residual confounding. In the revised manuscript, we provide information about the covariate adjustment in the parent studies (Methods lines 449 to 454).

2) The authors confound migraine vs. headache in the references of the introduction. Clinically, BP lowering is not used to avoid headaches; headache and hypertension: a myth (e.g. [dx.doi.org/10.1136/jnnp.72.4.431](http://dx.doi.org/10.1136/jnnp.72.4.431)), it should be mentioned that BP is higher when individuals have pain; but should hypertension and migraine occur at the same time, beta blockers are a valid method of treating both. A few phrases in the introduction are contrary to these quite well established clinical findings. The phenotypes used need to be explained much more carefully as does the genotyping.

**Response:**

We thank the reviewer for this comment. In the revision, we address this relationships between migraine, headache, and BP more comprehensively than in the original manuscript. In the Introduction we now write: “Blood pressure (BP) has been associated not only with vascular disease but also with migraine. In contrast to highly consistent associations of increased BP with increased susceptibility to vascular disease, associations of BP with migraine are not consistent. For example, some studies have found associations between elevated SBP and DBP and lower prevalence of migraine while some have found inverse associations only for SBP. One suggested that migraine was associated with higher DBP but lower SBP compared to individuals without migraine. Still other reports focused on pulse pressure (PP), defined as the difference between systolic BP (SBP) and diastolic BP (DBP), consistently showed an inverse relationship between PP and migraine. The relationship is further complicated by longitudinal studies suggesting that migraine may increase the risk of incident hypertension while blood pressure has been found to be inversely related to onset of headache and migraine. Regardless, BP lowering medications notably provide prophylactic benefit for many migraineurs, and the choice of antihypertensive appears to be related to comorbidities, cost, availability, or side effect profile rather than the physiologic mechanism”, and we return to the comparison of the observational studies to our findings in the last sentence of the first paragraph of the Discussion. We write: “In fact, the findings from genetics are concordant with at least one of the prior observational studies” in lines 390 to 391.

As for the phenotypes and genotypes, we used GWAS summary level data for both migraine and BP traits for all the analysis in the current study, and almost exclusively did not use individual phenotype or genotype data. The exception was for replication of 14 novel migraine loci identified from cross-trait analysis through individual-level data for 14 SNPs in UK Biobank data. We have reviewed the entire text and supplementary notes to make sure the phenotype and genotype data are properly described.

3) The cross-train meta-analysis is very difficult to interpret. What do these loci mean, given the very weak association between migraine and BP?

**Response:**

We agree that the genetic correlation between BP and migraine is modest (largest genetic correlation is r_g_=0.11). However, this circumstance does not preclude the possibility that some loci are strongly associated with both and may reflect shared etiologies. We were applied high standards for interpretation of loci with shared signals as follows. First, we observed that previously identified migraine loci (*PHACTR1*, *LRP1,* *FHL5*, *C7orf10*, *MPPED2*, *CFDP1*, and *SLC24A3*) involved in vascular functions, including vascular development, endothelial structure, and smooth muscle function also revealed shared signals with BP. This result indicates that cross-trait meta analyses are sensible for migraine and BP. Second, we required that all loci reaching genome-wide significance in joint analysis (*P*< 5×10^-8^) and also had suggestive significant association (*P*< 10^-5^) separately for migraine and the BP phenotype. Finally, we focused on those loci that were identified from not only at genome-wide significance in cross-trait meta-analyses but also in transcriptome wide association analysis (TWAS) thereby requiring that the identified loci have both SNP-level shared signals and gene/expression-level shared signals for BP and migraine. Therefore, the concordance of findings from cross-trait meta-analysis and TWAS provides robust support for the involvement of the specific loci we highlight in both migraine liability and BP regulation, and therefore also points to shared etiology.

4) It is unclear how the p-values of the genetic-overlap results were derived and what is significant. The central figure is difficult to interpret in this sense.

**Response:**

Thank you for pointing this out. We used two methods to assess genetic overlap. ρ-HESS and GWAS-PW estimate local genetic correlation and its significance between BP traits and migraine at 1703 pre-specified LD independent segments across the genome. We write: “ρ-HESS quantifies the local genetic covariance (and correlation) and P values (P ρ-HESS) between pairs of traits at local regions, while GWAS-PW uses a Bayesian framework to estimate the posterior probability (PPA_3) that genetic associations with the two traits co-localize using priors that are learned from the data. ”. Detailed results for these two analyses were presented in Supplementary table 1 and 2. For both methods, we use standard significance thresholds that account for multiple testing across the whole genome. We now provide these significance thresholds in lines 485 to 487 as “BP and migraine were considered to have genetic correlation at local region if P ρ-HESS was significant after correcting for multiple testing (P ρ-HESS<0.05/1703) and PPA_3 from GWAS-PW was larger than 0.9.”. In addition, to make the figures easier to interpret, we have added a clarifying sentence in the Figure 1 legend.

5) The TWAS results are expressed as counts in Figure 2. The null distribution is not shown and it is unclear from the figure what is significantly different from the null.

**Response:**

As shown in the report describing the TWAS method, under the null hypothesis where expression is completely independent of phenotype (with either being heritable), the TWAS false positive rate is well controlled (see below for details).

| **GWAS size:** | **50,000** | **100,000** | **150,000** | **200,000** | **250,000** | **300,000** |
| --- | --- | --- | --- | --- | --- | --- |
| GE independent of cis-heritable trait | | | | | | |
| % PV < 0.05 | 1.8% | 2.5% | 3.3% | 3.9% | 4.4% | 4.5% |
| # simulations | 5952 | 5952 | 5952 | 5951 | 5951 | 5951 |
| Trait independent of cis-heritable GE | | | | | | |
| % PV < 0.05 | 1.2% | 2.2% | 2.7% | 2.6% | 2.4% | 2.4% |
| # simulations | 3000 | 3000 | 3000 | 3000 | 3000 | 3000 |

Fraction of TWAS simulation instances with P-value < 0.05 is shown for two simulated nulls over increasing GWAS sample sizes. Top: Expression is independent of SNPs and but trait/GWAS statistics are heritable. Bottom: Expression is heritable but trait/GWAS statistics are independent of SNPs (quoted from the TWAS paper published in Nature Genetics: https://www.nature.com/articles/ng.3506#MOESM262).

In the current investigation, a permutation test was run 1,000 times for each TWAS gene as implemented in TWAS procedure, and a P value was computed by Z test against this null of no expression-trait association (or genetic correlation between expression and a trait) conditional on the observed GWAS statistics at the locus. We have added this information in Methods to clarify (lines 505 to 509). We write “The null hypothesis of TWAS is no expression-trait association (or genetic correlation between expression and a trait) conditional on the observed GWAS statistics at the locus. In practice, a permutation test based on 1,000 resampling iterations was run for each TWAS gene to ensure that the TWAS false positive rate was well controlled”. Finally, we applied Bonferroni correction to identify significant expression-trait associations adjusted for multiple comparisons for all gene-tissue pairs tested for each trait (~230,000 gene-tissue pairs in total across 48 tissues, significant expression-trait associations were defined as *P*_Bonferroni_ <0.05).

6) The results contain many speculative comments on potential mechanisms that are unproven. The supplement is >100 pages and should be summarized much more.

**Response:**

We agree with the reviewer that the Supplement was too long, and we have shortened it to 65 pages of supplementary results (with additional 8 pages acknowledging authors from consortia) by removing some preliminary TWAS results. As for the unproven speculative comments on potential mechanisms in the results part, while our original intention was to present potential clues of shared etiologies between BP and migraine via the cross-trait and TWAS identified overlapped genes, we have now streamlined this to minimize speculation and only retained comments about known functions of the candidate genes in the results section.

7) Would it be appropriate to apply a multiple-testing correction to the MR-analyses?

**Response:**

Thank you raising this critical issue. In the revised manuscript, we now corrected for multiple testing (Bonferroni) in the MR analysis. All findings remain significant with the exception of the instrumental effect of DBP on MA and SBP on MO, both of which are nominal significant without correction (*P* =0.02 and 0.04, respectively). The results section and corresponding table have been updated accordingly.

MINOR
- Line 76: should state what type of "dissection"

**Response:**

We now specify “cervical artery dissection”.

Reviewer #3 (Remarks to the Author):

This report from Guo and Chasman explores the genetic relationships between blood pressure traits and migraine. This is an important question as it could help establish new treatment modalities for migraine treatment/prevention using blood pressure lowering medications. The analyses are thorough and methodologically sound. I do have some suggestions for the authors:

1) Have the authors tried analyzing MAP in addition to SBP, DBP and PP? This would nicely complement current analyses.

**Response:**

The investigation used the latest summary level data from the International Consortium of Blood Pressure-Genome Wide Association Studies (ICBP) GWAS of DBP, SBP, and PP but not MAP among 757,601 participants. At the same time, the clinical epidemiology of migraine has focused on DBP, SBP, and PP but not MAP. This is why MAP was not included. However, we agree that reviewer raises a valid issue. Using smaller scale GWAS data sets for BP (N~450,000) and the GWAS for migraine that is described in the current manuscript, we separately estimated the genetic correlation between MAP and migraine. This value is 0.09 (p=4.89e-5), which is intermediate between the genetic correlation we report for DBP and SBP in our current analysis.

2) The gene-level genetic correlation is interesting as it appears some genes disproportionally contribute genetic correlation. Would results of the MR analysis hold if these genes were removed? This is relevant as bi-directional MR associations could potentially be explained by some loci affecting both traits (rather than one trait affecting the other and vice versa).

**Response:**

We agree that instruments with pleiotropic effects (i.e. affecting both traits simultaneously rather than affecting one trait then the other) would bias the MR estimates, and in fact, violate the assumptions of MR. The issue raised by the reviewer is a concern for all but the most simple MR applications and the state-of-the art is to exclude SNPs with statistical properties consistent with potential pleiotropy. For our MR analyses, we first used the GSMR package that includes such a procedure (termed HEIDI for heterogeneity in dependent instruments) to exclude SNPs with apparent pleiotropic effects from the MR estimate (PHEIDI<0.01). We further conducted sensitivity analyses by MR-egger (Egger regression) that will return an unbiased estimate in the presence of “directional” pleiotropy, and the results were quite consistent. We provide details about these measures to address pleiotropy in the last paragraph of the Methods.

3) The opposing associations of SBP and DBP on migraine is intriguing. Could this explain the inconsistent epidemiological associations? If possible, attempting to do so would add to the insightfulness of the paper.

**Response:**

Thanks for these comments. We only observed frankly opposite associations of SBP and DBP on migraine in conditional MR analyses, i.e., when controlling for SBP, higher DBP is positively associated with migraine, while when controlling for DBP, lower SBP is positively associated with migraine. Both of these findings are consistent with the instrumental results of PP, a positive association with migraine.

Reviewer 2 encourages us to revise the context for the investigation as described in the Introduction. In doing so, we identified at least one, robust, large scale investigation that supports the predominance of DBP in migraine and even the potential inverse association with SBP. The Introduction of the revised manuscript now refers to this observation. Similarly, the Discussion raises possible explanations for the inconsistent epidemiology but tries to avoid too much speculation. In the Discussion, we also reiterate well-known reasons for relatively lower risk of (residual) confounding and reverse causality in inference using genetics, while again noting the consistency with the newly identified reference.

Guillaume Pare

**Reviewer comments, second version:**

Reviewer #1 (Remarks to the Author):

The authors have adequately responded to this reviewer's critique. No further comments.


Reviewer #2 (Remarks to the Author):

This is a careful revision and a lot of work. The estimate for genetic correlation between the two traits remains small (r2=1%), but this is expressed in a satisfactory way now. Good luck to the authors.


Reviewer #3 (Remarks to the Author):

N/A

**Author rebuttal, second version:**

Reviewer #1 (Remarks to the Author):

The authors have adequately responded to this reviewer's critique. No further comments.
**Response:** Thank you so much for all your comments and positive remarks on our manuscript.

Reviewer #2 (Remarks to the Author):

This is a careful revision and a lot of work. The estimate for genetic correlation between the two traits remains small (r2=1%), but this is expressed in a satisfactory way now. Good luck to the authors.
**Response:** Thank you so much for your positive remarks on our manuscript and all your comments which help us making great improvements on our manuscript.

Reviewer #3 (Remarks to the Author):
**Response:** Thank you so much for all your comments and positive remarks on our manuscript.

**Reviewer comments, third version:**

**Author rebuttal, third version:**
